# Novel C3-Methylene-Bridged Indole Derivatives with and without Substituents at N1: The Influence of Substituents on Their Hemolytic, Cytoprotective, and Antimicrobial Activity

**DOI:** 10.3390/ijms25105364

**Published:** 2024-05-14

**Authors:** Karolina Babijczuk, Natalia Berdzik, Damian Nowak, Beata Warżajtis, Urszula Rychlewska, Justyna Starzyk, Lucyna Mrówczyńska, Beata Jasiewicz

**Affiliations:** 1 Department of Bioactive Products, Faculty of Chemistry, Adam Mickiewicz University, Uniwersytetu Poznańskiego 8, 61-614 Poznań, Poland; babijczukk@gmail.com (K.B.); natalia.berdizk@amu.edu.pl (N.B.); 2 Department of Quantum Chemistry, Faculty of Chemistry, Adam Mickiewicz University, Uniwersytetu Poznańskiego 8, 61-614 Poznań, Poland; damian.nowak@amu.edu.pl; 3 Department of Crystallography, Faculty of Chemistry, Adam Mickiewicz University, Uniwersytetu Poznańskiego 8, 61-614 Poznań, Poland; beata.warzajtis@amu.edu.pl (B.W.); urszula.rychlewska@amu.edu.pl (U.R.); 4 Department of Soil Science and Microbiology, Faculty of Agronomy, Horticulture, and Bioengineering, University of Life Science, Szydłowska 50, 60-656 Poznań, Poland; justyna.starzyk@up.poznan.pl; 5 Department of Cell Biology, Faculty of Biology, Adam Mickiewicz University, Uniwersytetu Poznańskiego 6, 61-614 Poznań, Poland; lumro@amu.edu.pl

**Keywords:** gramine, indole derivatives, thione derivatives, anti-oxidant properties, oxidative hemolysis, docking study, crystal structures

## Abstract

Alkaloids are natural compounds useful as scaffolds for discovering new bioactive molecules. This study utilized alkaloid gramine to synthesize two groups of C3-substituted indole derivatives, which were either functionalized at N1 or not. The compounds were characterized by spectroscopic methods. The protective effects of the new compounds against in vitro oxidative hemolysis induced by standard oxidant 2,2′-azobis(2-amidinopropane dihydro chloride (AAPH) on human erythrocytes as a cell model were investigated. Additionally, the compounds were screened for antimicrobial activity. The results indicated that most of the indole derivatives devoid of the N1 substitution exhibited strong cytoprotective properties. The docking studies supported the affinities of selected indole-based ligands as potential antioxidants. Furthermore, the derivatives obtained exhibited potent fungicidal properties. The structures of the eight derivatives possessing indole moiety bridged to the imidazole-, benzimidazole-, thiazole-, benzothiazole-, and 5-methylbenzothiazoline-2-thiones were determined by X-ray diffraction. The C=S bond lengths in the thioamide fragment pointed to the involvement of zwitterionic structures of varying contribution. The predominance of zwitterionic mesomers may explain the lack of cytoprotective properties, while steric effects, which limit multiple the hydrogen-bond acceptor properties of a thione sulfur, seem to be responsible for the high hemolytic activity.

## 1. Introduction

Alkaloids are a large group of naturally occurring compounds used as precursors for synthesizing new drugs [1,2,3,4]. Among them, the indole alkaloids, such as vinca alkaloids (vincristine and vinblastine), reserpine, or ergot alkaloids, are of significant pharmacological interest [5,6,7,8,9,10]. Another indole alkaloid that has been constantly receiving increasing attention in sustainable chemistry is gramine. This earth-abundant natural compound is used as a pharmaceutical lead scaffold for synthesizing indole-based compounds with different biological activities [11,12,13,14,15,16]. The dimethylamine group at the C3 position of the indole ring in gramine can undergo substitution reactions [17,18]. Therefore, functionalizing this position is convenient for obtaining more intricate structures or starting materials for further functionalization and reactions. Many C3-substituted indole analogs are effective as anticancer, antitubercular, antimicrobial, and antioxidant agents [5,9,19,20]. Another modification in the structure of gramine involves substituting the nitrogen atom in the indole group. *N*-substituted indole derivatives have shown anti-inflammatory, antimicrobial, antipsychotic, antifungal, and antioxidant effects [14,21,22,23,24]. Indole derivatives with tertiary amino and phenyl groups at the N1 nitrogen atom exhibit significant activity against the *Staphylococcus aureus* pathogen [24].

**Figure 1:**
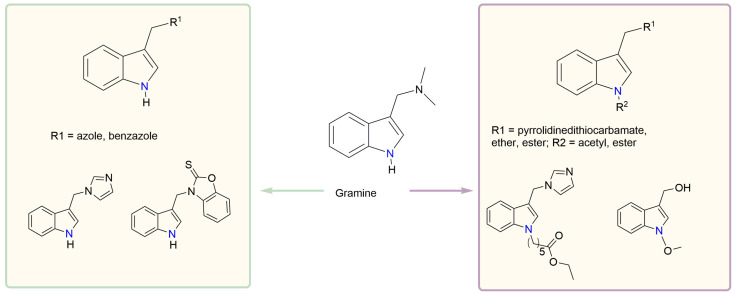
Design of the new gramine derivatives. Examples of bioactive indole derivatives aubstituted at the C3 position (left) or at the C3 and N1 positions (right).

We synthesized two groups of new indole-based derivatives based on the literature data (Figure 1) to evaluate their selected biological activity. One group of molecules consisted of indole derivatives, featuring substituents at both the C3 and N1 positions. Another group of gramine derivatives contained indole moieties attached by a methylene linker at the C3 position to azoles or benzazoles. 

Azole-based compounds are essential building blocks in many pharmaceutical agents, and they have various effects, including antimicrobial properties [25,26,27,28,29]. Their activities are explained by the existence of a tautomeric equilibrium. Tautomers differ in their molecular shapes and proton donor–acceptor properties; therefore, depending on the tautomeric form, they can be involved in different molecular interactions between themselves or with other targets. In the investigated series that comprise the azole-2-thione moieties, the molecules can theoretically exist in two tautomeric forms, viz., thione and thiol. The thione form provides the only ‘classical’ hydrogen bond acceptor site, the other possibility being the engagement as a hydrogen bond acceptor site of π-electron systems or solvation. 

In the studies of membrane-active compounds for any biomedical application, human red blood cells (RBCs) are commonly used as a cell model [30,31,32]. Due to their availability and lack of organelles, they are used to evaluate the cytotoxicity of newly synthesized compounds. 

RBCs are an excellent model cell for antioxidant studies due to their membrane, which has a high polyunsaturated fatty acid content. Furthermore, RBCs with hemoglobin containing heme iron are continuously exposed to oxidation during oxygen transport [33,34]. ROS (reactive oxygen species) are both radical (superoxide O_2_^•−^, hydroxyl OH^•^, peroxyl RO_2_^•^, hydroperoxyl HO_2_^•^) and nonradical (hydrogen peroxide H_2_O_2_, hypochlorous acid HOCl, and ozone O_3_) forms of oxygen. They are formed through enzymatic and non-enzymatic processes, and they can be acquired from external sources, such as food, UV radiation, or environmental pollution [35]. Although ROS play a significant role in signal transduction [36], excess can lead to an oxidative stress that causes cancer, diabetes, and neurodegenerative diseases [37,38].

Exposure of RBCs to oxidative stress can consequently result in changes in the molecular structure of their cell membrane, thus leading to hemolysis. Therefore, one of the main criteria limiting the in vitro use of new bioactive compounds is the evaluation of their hemocompatibility. Compounds considered hemocompatible [32] are not toxic to all cells. 

Cells have developed a series of antioxidant defense systems to scavenge or minimize the formation of oxygen-derived radicals, thereby protecting themselves from oxidative damage. These antioxidant systems include dietary antioxidants and endogenous enzymatic and non-enzymatic constituents. Enzymatic antioxidants, which act as catalysts, are responsible for removing ROS from biological systems. Superoxide dismutase catalyzes the conversion of O_2_^•−^ to H_2_O_2_, while H_2_O_2_ can be reduced to water by catalase or glutathione peroxidase through two distinct mechanisms. Hydroxyl radicals are generated in the Fenton reaction from hydrogen peroxide in the presence of Fe (II) or Cu (I), and they are neutralized by glutathione peroxidase [38,39]. The most common dietary antioxidants include vitamins A, C, and E, as well as flavonoids and alkaloids [40,41].

This study aimed to utilize alkaloid gramine in the synthesis of two new groups of indole derivatives with altering substituents at C3 and N1, determine their structures and spectroscopic characteristics, and in vitro evaluate their hemocompatibility and cytoprotective activity under oxidative stress conditions. In addition, an in silico docking study was conducted to estimate the affinity of the obtained compounds for three protein domains: myeloperoxidase (MPO), xanthine dehydrogenase, and cyclooxygenase-2 (COX-2). These enzymes are involved in generating ROS and contributing to oxidative stress [42,43,44,45,46,47]. Since gramine and its derivatives have demonstrated the potential to inhibit the growth of certain bacterial and fungal species [12], the compounds obtained were also preliminarily screened for in vitro antimicrobial activity.

## 2. Results and Discussion

### 2.1. Synthesis and Spectroscopic Characterization of New Indole Derivatives

**Figure 2:**
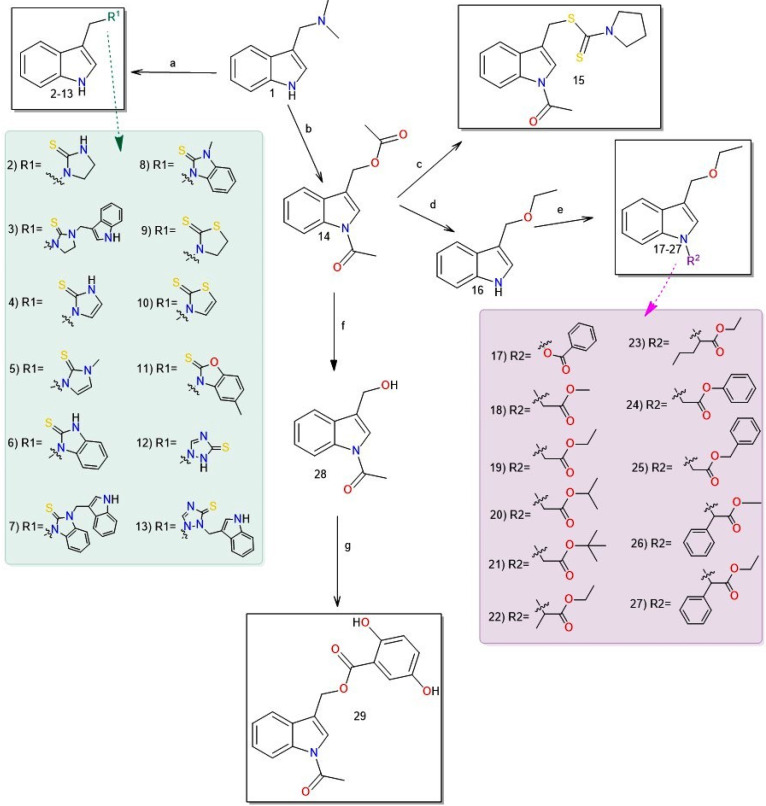
Synthesis of Compounds **2**–**29**: (a) H-R^1^, EtOH, NaOH, reflux (**2**–**9**) or H-R^1^, EtOH, and reflux (**10**–**13**); (b) Ac_2_O and reflux; (c) sodium pyrrolidinedithiocarbamate, H_2_O, and reflux; (d) EtOH, base, and r.t.; (e) CHCl_2_, benzoic acid, PPh_3_, NBS, Et_3_N, and 0 °C; (**17**) Br-R^2^, DMF, base, and 0 °C (**18**–**27**); (f) H_2_O and reflux; or (g) THF, 2,5-dihydroxybenzoic acid, DCC, and r.t.

Gramine (**1**), a commercial indole alkaloid, was used as the substrate for the synthetic routes, as shown in Figure 2.

Compounds **2**–**13** were synthesized by heating gramine with the corresponding substrate in ethanol. Derivatives **2**–**9** were synthesized in an alkaline medium, which was achieved by adding NaOH to the substrate in ethanol before adding gramine and heating. Dimers **3** and **7** crystallized first, and, after adding water to the filtrate, Monomers **2** and **6** appeared as solids, which were then filtered. In the reaction with 1,2-dihydro-1,2,4-triazole-3-thione (i.e., a synthesis of **12** and **13)**, column chromatography was necessary to separate the dimer and monomer forms. Heating gramine in acetic anhydride produced *N*-acetyl-3-acetoxymethylindole (**14**), which can be converted to Compound **15**. Compound **14** was reacted with anhydrous ethyl alcohol in an alkaline medium to produce 3-ethoxymethylindole (**16**). The resulting ether derivative (**16**) was then treated with benzoic acid to yield Compound **17**. Compounds **18**–**27** were obtained by reaction of Derivative **16** with a series of bromoesters. Derivative **29** was synthesized through a two-step process involving the hydrolysis of *N*-acetyl-3-acetoxymethylindole (**14**), which was followed by a reaction with 2,5-dihydroxybenzoic acid. 

**Figure 3:**
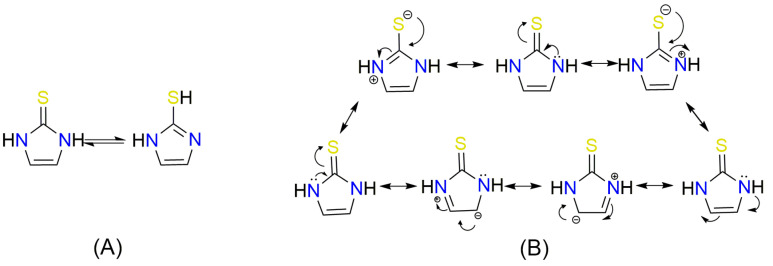
(**A**) The tautomeric forms of imidazole-2-thione. (**B**) The mesomeric effect in imidazole-2-thione.

A crucial structural feature of azoles and benzazoles is the existence of tautomeric forms. Figure 3 shows the following: (**A**) two tautomeric forms of imidazole-2-thiones (thione and thiol) and (**B**) their mesomeric effect. According to the literature data, the thione form is predominant in polar solvents and the solid state [16,48,49,50,51,52,53,54,55].

The NMR spectra analysis confirmed the presence of the thione forms of the newly obtained gramine derivatives (**2**, **4**, **6**, **12**, and **13**) in the DMSO-*d*_6_ solution. 

The newly synthesized compounds (**2**–**13**, **15**, **17**–**27**, and **29**) exhibited characteristic signals for the aromatic indole system in the 8.50–6.20 ppm range, as observed in their ^1^H NMR spectra. Additionally, the signals from the azole rings and phenyl (17, 24–27, and 29) substituents were visible in the aromatic region (7.00–8.50 ppm). The singlets at 11–14 ppm were assigned to the NH protons of the gramine moiety (**2**–**13**) and the imidazole-, benzimidazole-2-thione or triazole-3-thione rings (**4**, **6**, **12**). The protons of the –C(10)H_2_– groups of all the new compounds showed singlets in the range of about 4.65–5.80 ppm. The singlets from the -CH_3_ group present at the nitrogen atoms in 5 and 8 were at 3.48 and 3.75 ppm, respectively. The singlet from the methyl group at position C5 in **11** was at 2.33 ppm. A singlet near 2.60 ppm was observed for three protons from the acetyl group (15, 29). 

The ^13^C NMR spectra of the new compounds showed signals from the carbon atoms of the indole rings at 109–162 ppm. Compounds **17**, **24**–**27**, and **29** also exhibited signals in the 109–158 ppm range, which originated from the phenyl substituents. Additionally, the spectra of Compounds **17**–**27** displayed signals from the carbonyl carbon atoms at about 166–171 ppm. The acetyl group signal for Compounds **15** and **29** was observed at approximately 168 ppm. The thione group signal was located at approximately 160 ppm (**4**–**8**, **12**, and **13**) or 180–195 ppm (**2**, **3**, and **9**–**11**). Additionally, the signals from the azole (**4**, **5**, **10**, **12**, and **13**) and benzazole (**6**–**8** and **11**) rings were present in the range of 111–152 ppm. The signals corresponding to the carbon atoms at the C10 position of all compounds occurred in the 33–69 ppm region. The carbon signal from the methyl group was connected to the nitrogen atom in **5** and **8**, and was approximately 33 ppm, while the signal from the methyl group in Position C5 in **11** was at 20.92 ppm. The carbon signal from the -CH_3_ in the acetyl group (**15** and **29**) was present at 24.23 ppm and 29.92 ppm, respectively.

The structures of all the new indole derivatives were also confirmed by EI-MS and IR spectroscopy, as well as elemental analysis.

The FT-IR spectra of all the compounds in the KBr tablets exhibited characteristic absorption bands of 3050–2800 cm^−1^, which corresponded to the C-H bonds of the aromatic rings. Furthermore, in the spectra of Compounds **2**–**13**, a wide band at 3500–3200 cm^−1^ was present, thereby corresponding to the stretching vibrations of N-H in the indole ring. The carbonyl group exhibited an intense stretching vibration peak at approximately 1700 cm^−1^ (**15**, **17**–**27**, and **29**). Stretching vibrations of C=S were observed from 1000 cm^−1^ to 1300 cm^−1^. The FT-IR spectrum of Compound **29** showed a broad absorption band with a maximum of 3215 cm^−1^, thereby indicating the O-H bond vibrations of the hydroxyl groups.

The EI-MS spectra of all the newly synthesized compounds showed signals corresponding to molecular ions, with relative abundances ranging from 2 to 100%. For derivatives **2**–**13** and **15**, ions with an intensity of 100% were identified at *m*/*z* = 130 (C_9_H_8_N)^+^. 

The NMR (^1^H and ^13^C), EI-MS, and FT-IR spectra of the investigated compounds are provided in the Supplementary Materials (Figures S1–S25).

### 2.2. X-ray Analysis

**Figure 4:**
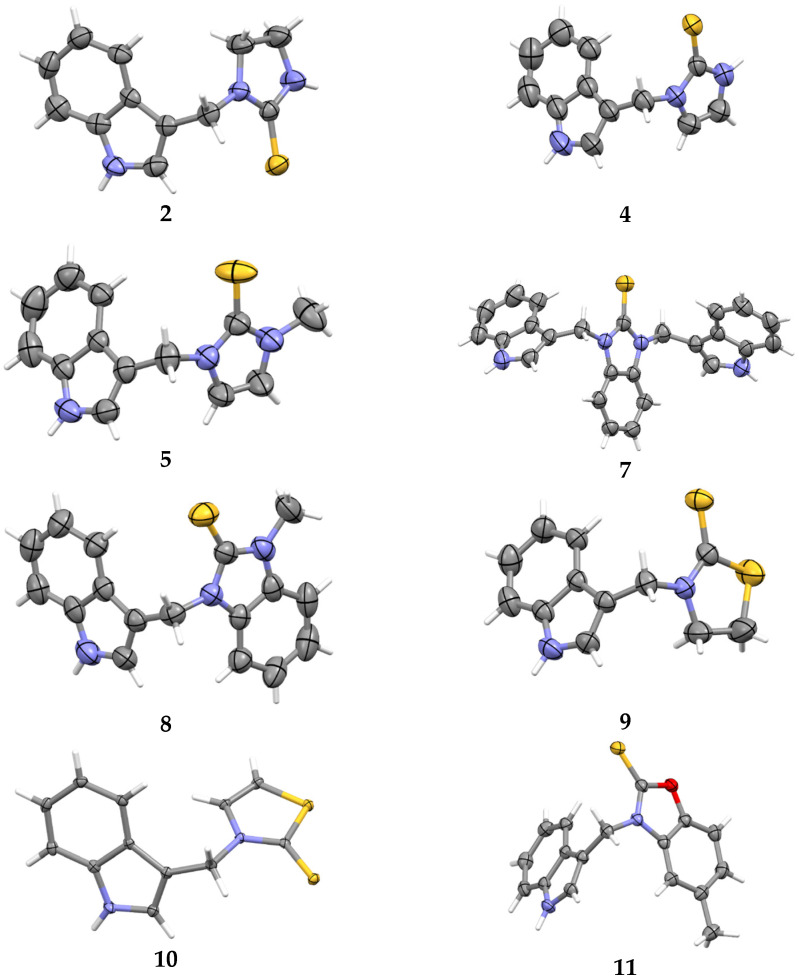
Perspective views of the molecules as present in the crystals of Compounds **2**, **4**, **5**, **7**–**9** (room temperature structures), and **10**, **11** (100 and 130 K structures, respectively). The thermal ellipsoids were all drawn at the 50% probability level; hence, they are smaller for the Low-Temperature structures **10** and **11**, and the H-atoms are represented as sticks.

We investigated, by X-ray diffraction, a series of eight compounds (**2**, **4**–**5**, and **7**–**11**), in which indole moiety was bridged by the –CH_2_– group to the five-membered heterocyclic fragments containing altered imidazole-, benzimidazole-, thiazole-, benzothiazole-, and 5-methylbenzothiazoline-2-thiones (Figure 2). The structures of the molecules, as seen in the crystals, are presented in Figure 4. The hydrogen bond geometrical parameters with intramolecular interactions are presented in Table S1. The crystal data, together with the experimental and refinement details, are shown in Table S2.

The molecules in all of the investigated crystals appeared in a thione form. The C=S bonds in the thioamide fragment in Compounds **2**, **4**, **5**, **7**, and **8** measured at room temperature varied from 1.679(3) to 1.697(2) Å, with the mean value of 1.685(7) Å. The value was between that which is typical for single and double bonds. This was rationalized in terms of a substantial involvement of zwitterionic structures, as presented in Figure 3B [55]. In particular, the thione tautomer in **4** had a more significant contribution of the zwitterionic forms that involve single C^+^-S^−^ covalent bonds than any other structure. The main skeleton can be described as consisting of two methylene-bridged subunits, each containing aromatic rings, which are inclined with respect to each other at angles varying from 66.6 to 87.9°. One of the fragments was always a C3-substituted planar indole moiety, while the others were N-substituted 2-thione derivatives of imidazole (**2**, **4**, and **5**), benzimidazole (**7** and **8**), thiazole (**9** and **10**), and 5-methylbenzoxazole (**11**). A description of the molecular conformation was provided by a pair of torsion angles (φ_1_ and φ_2_) measured along the C-C-C-N and C-C-N-C methylene bonds, which are listed in Table 1. 

To enable an easier comparison, Table 1 also provides chemical diagrams and a capped stick representation of the molecules, all of which were seen in the same orientation, i.e., along the indole plane. This allowed us to combine the values of the torsion angles with a particular molecular shape.

**Figure 5:**
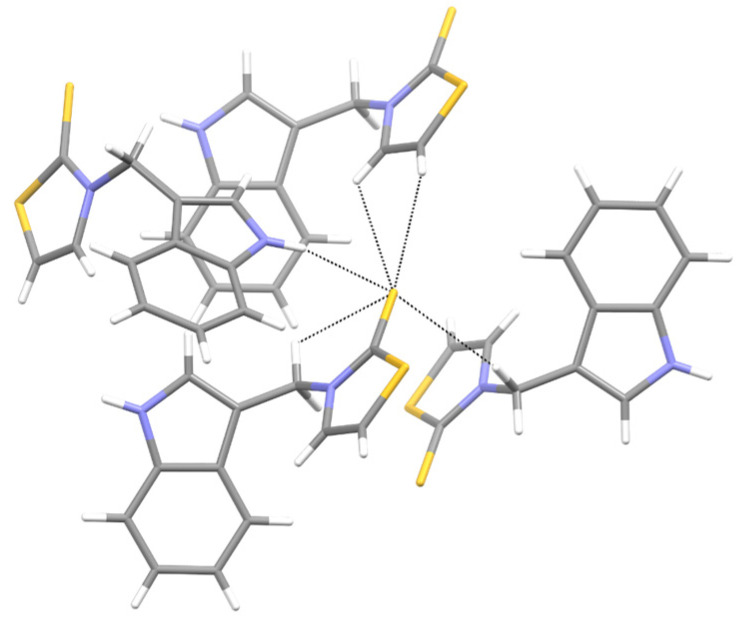
An example from the thiocarbonyl group acting as a quintupole hydrogen-bond acceptor in the crystal structure of **10**. The formed supramolecular motifs extended in three dimensions.

Except for **10**, the investigated molecules adopted similar, propeller-shaped conformation. The exceptional conformation of **10** could be due to the involvement of its thiocarbonyl group as a quintuple hydrogen bond acceptor (Figure 5, Table S1). The ability of the sulfur atom to simultaneously engage in a greater number of interactions than conventional acceptors such as O and N was evidenced by Bogdanovic and colleagues [56]. 

Moreover, a comparison of **10** with its benzothiazole analog [16] revealed that the latter analog totally excluded the C=S group from its involvement in intermolecular interactions. Although the alteration took place in the crystals, we were tempted to combine it with the finding that the compound with the benzothiazole-2-thione moiety neither displayed a cytoprotective or chelating ability, nor did it protect the RBCs from the oxidative stress-induced hemolysis [16]. Meanwhile, its homolog, **10**, with the thiazole-2-thione scaffold, showed significant cytoprotective activity and was hemocompatible (vide infra). 

**Figure 6:**
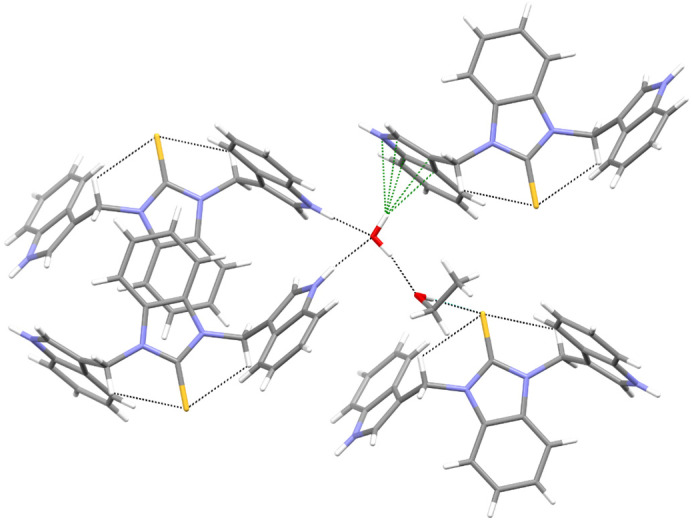
The whole palette of intermolecular interactions in the solvated crystals of **7**.

**Figure 7:**
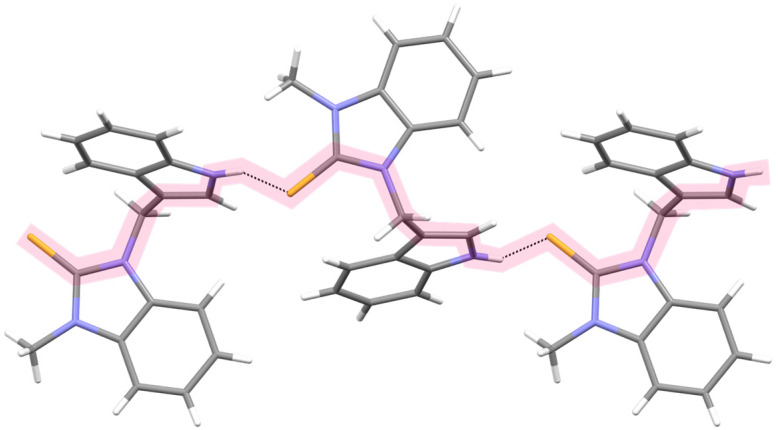
The helical arrangement of the hydrogen-bonded molecules of **8**. The bulky methyl groups and benzene rings were directed away from the HB-chain. The hydrogen bond was significantly bent due to steric hindrance (Table S1).

Unlike the thiazole-2-thione derivatives, the imidazole-2-thiones were less prone to the structural changes caused by chemical modifications. The isostructuralism of **5** and **9** (Table S2) indicated that the N-methyl group and sulfur atom are structural isosters, supposedly because neither of these fragments are involved in hydrogen bonding. Molecules **2**, **4**, and **7**, which contained two N-H hydrogen-bond donor groups, formed three-dimensional associates, either by taking advantage of the relatively easy approach of these groups to the thione sulfur (**2** and **4**), or by including solvent molecules to overcome the steric hindrance in an approach to the sulfur acceptor (**7**, Figure 6). The remaining derivatives (**5**, **8**, **9**, and **11**), having only one N-H donor group, associated into 1D chains or tapes (Figure 7). A detailed description of the molecular conformation and intermolecular interactions in the crystals of Compounds **2**, **4**–**5**, and **7**–**11** (Figures S26 and S27) is provided in the Supplementary Materials.

### 2.3. Biological Activity

#### 2.3.1. Antibacterial and Fungicidal Activity

A preliminary screening of the in vitro antimicrobial activity of gramine and its derivatives against pathogens microorganisms was studied using the well diffusion technique. Analysis of the interactions of the selected bacterial species with the tested compounds showed no antagonistic effects in most cases, except for Compounds **13** and **15**. Compound **13** exhibited antagonistic effects, as evidenced by the growth inhibition zones of *M. luteus* (7.3 mm), *B. subtilis* (9.4 mm), and *P. fluorescens* (10.5 mm). Derivative **15** was the most potent in inhibiting the growth of *M. luteus* and *E. coli*, thereby resulting in a zone of inhibition of 11 and 7.7 mm, respectively (see Table S3).

An analysis of the effect of gramine and its derivatives on the development of the tested mold species revealed that Compound **10** exhibited the strongest antagonistic reaction toward *B. cinerea*, with a growth inhibition zone of 23 mm (Table 2). This fungal species was also effectively inhibited by gramine and Compounds 3, 11–13, 21, 24, and 25, thus resulting in growth inhibition zones ranging from 11 to 19 mm. 

Most of the analyzed compounds (**1**, **3**, **6**–**8**, **17**–**20**, **22**, **23**, **26**, and **29**) significantly inhibited the growth of *T. atroviride*. The most-effective compounds were 6, 17, and 26, with growth inhibition zones of ≥20 mm. Derivatives **6** and **26** also had a clear impact on the growth of *Trichoderma* fungi, thus causing growth inhibition zones of 13 and 20 mm in *T. harzianum*, respectively. Compounds **5**, **12**, **13**, and **21** significantly limited the growth of *A. alternata*, with at least 10 mm inhibition zones. Compound **13** was also important in inhibiting the growth of *F. culmorum*, thereby causing the formation of a 13.2 mm growth inhibition zone.

#### 2.3.2. Cytoprotective Activity against Free Radicals

The ability of all compounds to inhibit 2,2′-azobis(2-amidinopropane dihydro- chloride (AAPH)-induced oxidative hemolysis was used to determine their cytoprotective activity. AAPH was widely used as a standard free radical inducer. During a temperature-dependent homolysis of AAPH, peroxyl and alkoxyl radicals were generated [57], thus leading to lipid peroxydation in the cell membranes [58]. In an AAPH assay, Trolox (TX), a water-soluble vitamin E, was used as a standard antioxidant [59].

**Figure 8:**
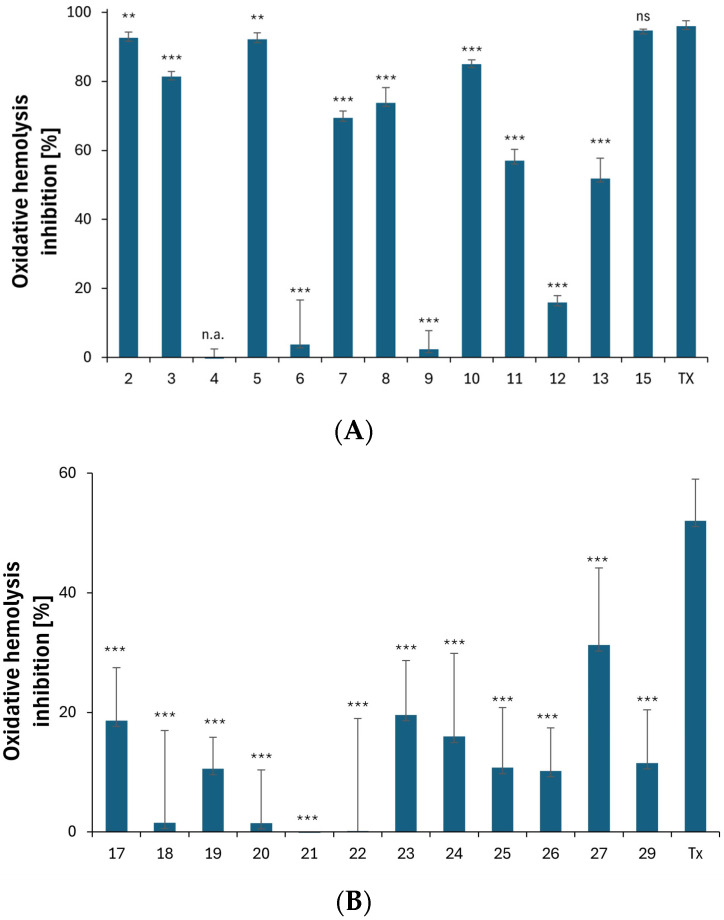
(**A**) Cytoprotective activity of Compounds **2**–**15** and the standard antioxidant Trolox at a concentration of 0.1 mg/mL against the oxidative hemolysis induced by free radicals generated from AAPH. The results (n = 9) are presented as the mean value ± standard deviation (*** *p* < 0.001, ** *p* < 0.01) in comparison with the standard antioxidant Trolox. The non statistically significant difference (*p* > 0.05) is indicated as ns. Inactive compounds are indicated as n.a. (**B**) Cytoprotective activity of Compounds **17**–**29** and the standard antioxidant Trolox at a concentration of 0.01 mg/mL against the oxidative hemolysis induced by free radicals generated from AAPH. The results (n = 10) are presented as the mean value ± standard deviation (*** *p* < 0.001) in comparison with the standard antioxidant Trolox. The non statistically significant difference (*p* > 0.05) is indicated as ns.

As shown in Figure 8A, most derivatives containing azole, benzazole, or pyrrolidine rings (**2**–**13**, **16**), in a concentration of 0.1 mg/mL, exhibited cytoprotective activities against oxidative stress in the range of 57.0% ± 3.20–94.7% ± 0.4. The most-effective derivatives were **2**, **5**, and **15**, with activity values of 92.7% ± 1.6, 92.2% ± 1.8, and 94.7% ± 0.4, respectively. These values are comparable to the standard antioxidant Trolox (96.0% ± 1.5). Compounds **2** and **5** were found to be hydrophilic, with logP values of 1.80 and 1.94, respectively. They contained polar substituents in Position C3 of the indole ring, resulting in a “polar head-non-polar tail” structure, which enhanced the stability of the RBC membrane by interacting in the lipid bilayer of the cell membrane [14,60]. The high cytoprotective activity also characterized Derivative **10** (84.9% ± 1.3), especially in comparison to its benzothiazole analog [16].

Derivatives **2**–**3**, **5**, **7**–**8**, and **10**–**11** had a substituent at the C3 position, which stabilized the resulting indolyl radical. Additionally, these compounds had an unsubstituted nitrogen atom N1, which further promoted the radical stabilization and enhanced their cytoprotective activity. However, Compound **15** showed high cytoprotective activity despite having a substituted N1 nitrogen atom. This result was likely due to the pyrrolidinedithiocarbamate moiety at the C3 position. 

Only four compounds showed a cytoprotective activity lower than 20%: **4**, **6**, **9**, and **12**. It was suggested that Derivatives **4**, **6**, and **12**, like 4-mercaptoimidazole [61], are predominantly in the zwitterionic form at a physiological pH, with a thiolate group that converts to a thiyl radical (RS^•^) in the presence of the free radicals generated by AAPH.

Thiyl radicals can cause the excessive generation of oxidants in erythrocytes, thereby leading to an imbalance in pro- and antioxidant levels. In addition, the thiyl radical can interfere with the lipid bilayer of RBCs by a direct addition to the double bonds in unsaturated fatty acids or by initiating the lipid peroxidation process by removing hydrogen from lipids [62,63]. It is noteworthy that Derivative **9** showed low cytoprotective activity (2.4% ± 5.3), which was attributed to the substituent in Position C3 of the indole ring. The thiazole-2-thione moiety cannot form resonance structures, which results in a lack of stabilization in the free radicals formed. 

Since most of the derivatives in the second group (**17**–**27** and **29**) were hemolytic at 0.1 mg/mL, antioxidant studies were performed at a 10-fold lower concentration of 0.01 mg/mL. The results are shown in Figure 8B.

Among all the derivatives, Derivative **27**, with a phenylacetate substituent in the N1 position, demonstrated the highest cytoprotective activity at 31.3% ± 12.9. The cytoprotective activity of the standard Trolox (Tx) was 52.1% ± 7.0. Compound **29** had an acetyl group in the N1 position and a dihydroxybenzoic substituent in the C3 position. Its cytoprotective activity value was surprisingly low at 11.5% ± 8.9, despite the high antiradical activity exhibited by the derivatives of benzoic acid, particularly with respect to its hydroxy derivatives [64,65]. 

The HAT (Hydrogen Atom Transfer) mechanism is one of the primary antioxidant mechanisms of indoles. The key to this mechanism is the hydrogen atom located on the nitrogen atom of the pyrrole ring [66,67]. Derivatives **17**–**27** and **29**, which have substitutions at the N1 position, may have low cytoprotective activity due their prevention of the formation of the indolyl radical.

#### 2.3.3. Chelating Activity

**Figure 9:**
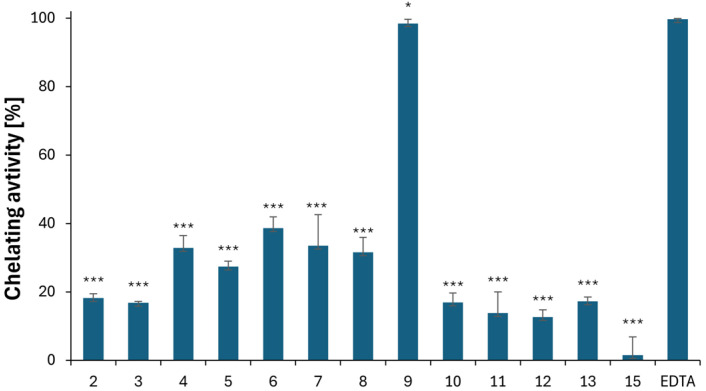
Ferrous ion chelating activity of the iron ions of Compounds **2**–**15** and the standard chelating agent EDTA. The results (n = 6) are presented as the mean value ± standard deviation (* *p* < 0.05, *** *p* < 0.001) in comparison with EDTA.

The hydroxyl radical ^•^OH is considered the most harmful free radical and is primarily responsible for the cytotoxic effects on aerobic organisms. It is formed in the presence of iron by the Haber–Weiss and Fenton reactions, where ferrous ions (Fe^2+^) are oxidized to ferric ions (Fe^3+^). Therefore, the ability of compounds to chelate Fe^2+^ ions can be used to evaluate their antioxidant properties. Compounds **2**–**13** and **15**, which contain heteroatoms with a lone electron pair (N and S), were investigated for their complexing activity. Figure 9 shows that most of the derivatives had ferrous chelating properties within the range of 1.4% ± 5.4 to 38.6% ± 3.2. However, only Compound **9** (98.5% ± 1.5) complexed Fe^2+^ ions more effectively than gramine, and it was found to be comparable to the standard chelator EDTA (99.7% ± 0.2). This derivative differed from all others because the electrons in its thiazoline-2-thione moiety were not involved in resonance, as in **2** and **4**, or in the aromatic system (as in Compound **10**), thus allowing them to be used for ferrous ion complexation. 

#### 2.3.4. Hemolytic Properties

The hemolytic activity of all derivatives has been assessed in vitro using human RBCs as a cell model. In general, a bioactive compound is considered hemolytic at a given concentration if it causes hemolysis of 5% or more of RBCs in a given sample [14,15,16]. Bioactive compounds that do not induce hemolysis of more than 5% of exposed RBCs are considered hemocompatible [68]. 

**Figure 10:**
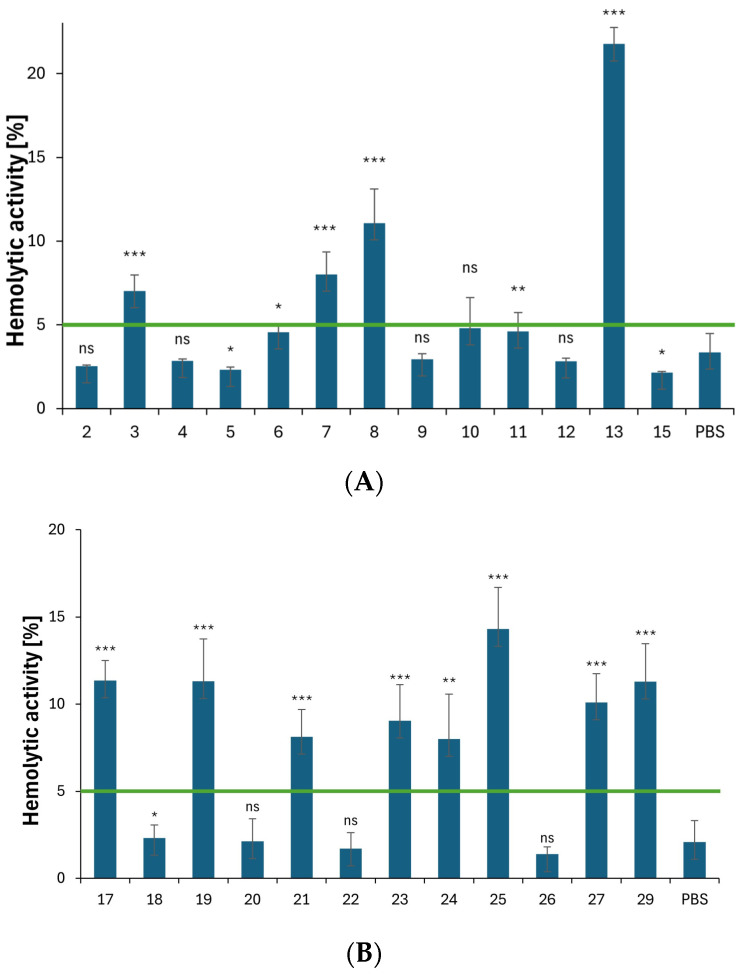
(**A**) Hemolytic activity of Compounds **2**–**15** at a concentration of 0.1 mg/mL. Results (n = 9) are presented as the mean value ± standard deviation (* *p* < 0.05, ** *p* < 0.01, and *** *p* < 0.001) in comparison with the standard buffer PBS. Non statistically significant difference (*p* > 0.05) is indicated as ns. The green line indicates a hemolysis threshold of 5%. (**B**) Hemolytic activity of Compounds **17**–**29** at a concentration of 0.1 mg/mL. Results (n = 9) are presented as the mean value ± standard deviation. (* *p* < 0.05, ** *p* < 0.01, and *** *p* < 0.001) in comparison with the standard buffer PBS. Non statistically significant difference (*p* > 0.05) is indicated as ns. The green line indicates a hemolysis threshold of 5%.

As shown in Figure 10A, the majority of derivatives with an azole or benzazole substituent at the C3 position of the indole ring are not hemolytic (hemolysis from 2.1% ± 0.1 to 4.8% ± 0.1). Compound **15**, which contains a pyrrolidinedithiocarbamate moiety, is also hemocompatible (2.1% ± 0.1). Compounds **3**, **7**, **8**, and **13** demonstrated high hemolytic activity, with values of 7.0% ± 0.9, 8.0% ± 1.3, 11.1% ± 2.0, and 21.8% ± 1.0, respectively. The high hemolytic activity of these compounds may be attributed to steric reasons, particularly the presence of two indole moieties. The impact of having two indole groups on the increase in hemolytic activity is evident when comparing the hemolytic activity values for Compounds 2 (2.5% ± 0.1) versus 3 (7.0% ± 0.9), 6 (4.6% ± 0.4) versus 7 (8.0% ± 1.3), and—especially for triazole derivatives—12 (2.8% ± 0.1) and 13 (21.8% ± 1.0). The hemolytic activity of Derivative **8**, which contains an *N*-methylated benzimidazole ring, increased from 4.6% ± 0.4 in the parent Molecule **6** to 11.1% ± 2.0. The increase in hemolytic activity can be attributed to changes in the molecular conformation of Compound **8** compared to Compound **6**, which resulted in different interactions with the lipid bilayer of the RBCs. No significant effects were observed when comparing the hemolytic activity values of Derivative **11** with those of the parent molecule (non-methylated at C5) [16], as well as the non-methylated Compound 4 with the N-methylated Compound **5**. In both cases, the hemolytic activity values were similar.

The hemolytic activity of Compounds **18**–**27** (Figure 10B) was dependent on the hydrophobicity of the ester substituents at the N1 position. It can be stated that the presence of these substituents enables incorporation into the phospholipid bilayer of RBCs. Among all the ester derivatives examined, Compounds **18**, **20**, **22**, and **26** exhibited no hemolytic activity (ranging from 1.4% ± 0.4 to 2.3% ± 0.7) and demonstrated hemocompatibility at the tested concentration.

### 2.4. In Silico Study

Lipinski’s and Verber’s rules provide criteria for determining whether new derivatives meet the requirements for a drug. According to Lipinski [69], a drug-like compound should have a molecular mass (MW) of less than 500 g/mol, an octanol/water partition coefficient (logP) of under 5, no more than 5 hydrogen bond donors (HBD), and 10 hydrogen bond acceptors (HBA). Veber’s rule [70] considers rotatable bonds (RTB) to be less than 10, and a polar surface area (TPSA) should not be greater than 140Å^2^. In addition to the drug-likeness parameters mentioned above, it is important to consider water solubility, gastrointestinal absorption (GI absorption), and blood–brain barrier penetration (BBB permeability). The physicochemical properties of the derivatives were evaluated using the SwissADME website [70]. Table 3 shows that all the derivatives met Lipinski’s and Verber’s rules and had high gastrointestinal absorption. Most could penetrate the blood–brain barrier (except for Compounds **3**, **10**, **12**, **15**, and **29**), which means they may act in the central nervous system. All compounds, except Compound **7**, were either soluble or moderately soluble in water. The low solubility of Compound **7** was due to its high lipophilicity, as indicated by its logP value of 4.78, the highest among all the compounds studied. 

### 2.5. Molecular Docking

Compounds **2**, **5**, and **15** were selected for molecular docking due to their cytoprotective activity above 90%. These compounds were found to be non-hemolytic. The selection of protein domains was guided by their specific biological functions within the physiological system. The chosen proteins, Myeloperoxidase (MPO), Xanthine dehydrogenase, and Cyclooxygenase-2 (COX-2), play crucial roles in cellular processes, and targeting them can have significant implications for modulating oxidative stress. These proteins can generate reactive oxygen spices (ROS) as part of the body’s defense mechanism against pathogens, or as a by-product of their enzymatic activity. Inhibition of their activity may reduce the generation of ROS associated with their function, thereby reducing oxidative stress [42,43,44,45,46,47]. 

The molecular docking data revealed that the newly acquired indole-based derivatives indeed exhibited affinity for the investigated protein domains. In Table 4, their affinity to the 1DNU protein domain is notably comparable to the reference ligand, melatonin. The ProteinsPlus algorithms, namely PoseView [71,72] and PoseEdit [71,73], were unable to produce 2D maps of the interactions. The following error was raised: “No interactions found by the PoseView interaction model”. This indicated that Compounds **15** did not have 2D depictions of the interactions between the protein domain and them. This raised the issue of whether it can be connected with the different settings and then used in UCSF Chimera 1.16 software [74]. Moving on to the 1N5X protein domain, the affinity of the indole-based derivatives was similar to the reference ligand, febuxostat. However, their affinity remained quite similar, indicating comparable binding tendencies. Similarly, for the 4COX protein domain, the affinity of the indole-based derivatives was slightly lower than that of the native ligand, indomethacin. Nevertheless, this disparity still suggests promising opportunities for its binding to the protein domain. 

**Figure 11:**
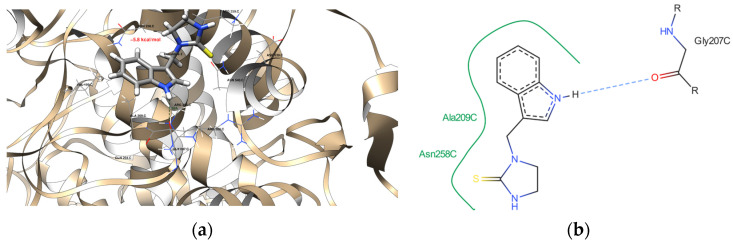
(**a**) The interactions between Derivative **2** and the 1DNU protein domain. The dark green dashed line suggests a potential hydrogen bond formation between the GLY 207 C residue and one of the hydrogen atoms bonded to the pyrrolic nitrogen atom of the ligand, with a length of 2.59 Å. (**b**) A detailed view of the interactions proposed by the molecular docking between the 1DNU protein domain’s binding site and Derivative **2**. One hydrogen bond was expected (blue dashed line), and the hydrophobic contacts are indicated by the green solid lines.

**Figure 12:**
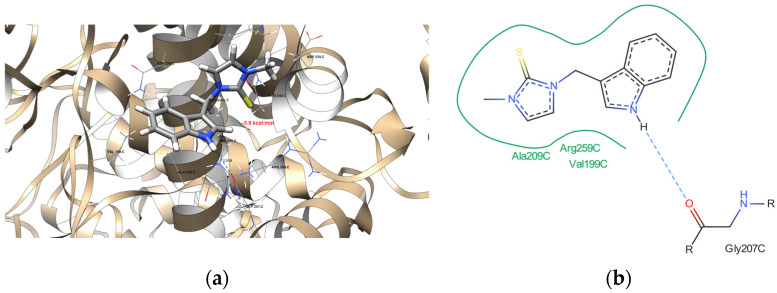
(**a**) The interactions between Derivative **5** and the 1DNU protein domain. The dark green dashed line suggests a potential hydrogen bond formation between the GLY 207 C residue and the pyrrolic hydrogen atom of the ligand, with a length of 2.29 Å. (**b**) A detailed view of the interactions proposed by the molecular docking between the 1DNU protein domain’s binding site and Derivative **5**. One hydrogen bond was expected (blue dashed line), and the hydrophobic contacts are indicated by the green solid lines.

**Figure 13:**
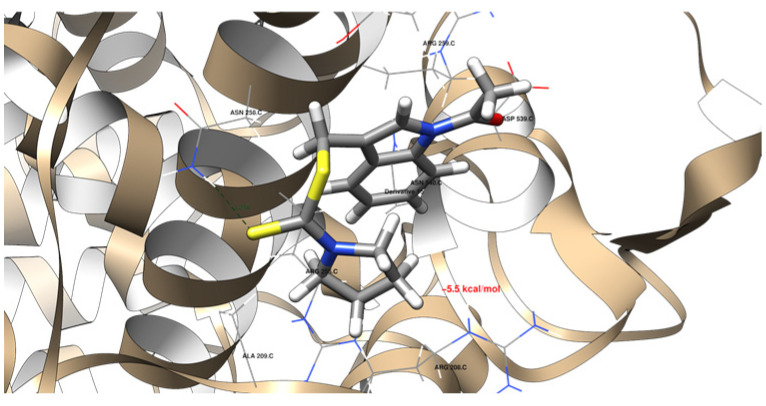
The interactions between Derivative **15** and the 1DNU protein domain. A dark green dashed line indicates a potential hydrogen bond formation between the protein residues ASN 258 C (3.29 Å length) and the sulfur of the ligand.

**Figure 14:**
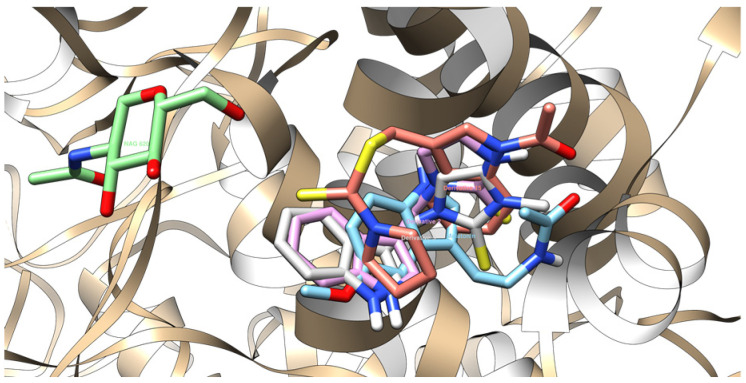
The binding site of the 1DNU protein domain features the reference ligand (melatonin), the native ligand (NAG620), and the investigated Compounds **2**, **5**, and **15** simultaneously.

Figure 11, Figure 12, Figure 13 and Figure 14 provide visual representations of the interactions between the indole-based derivatives and the 1DNU protein domain (PDB ID). Correspondingly, Figures S28–S31 (Supplementary part) depict the interactions between indole-based derivatives and the 1N5X protein domain (PDB ID). It is noteworthy that the recreation of the native ligand’s initial pose in the latter case has an acceptable accuracy, with a Root Mean Square Deviation (RMSD) of 2.635 Å [75]. Figures S32–S35 (Supplementary Materials), illustrate the interactions between indole-based derivatives and the 4COX protein domain (PDB ID). In this case, the recreation of the native ligand’s initial pose exhibits good accuracy, with an RMSD of 0.953 Å, which is considered satisfactory in the recreation of the initial pose [75]. These visualizations provide insights into the intricate molecular interactions underlying the binding of indole-based derivatives to the respective protein domains, thus reinforcing their potential as candidates for further exploration and development. All the molecular docking results are stored in Table 4.

The conducted studies indicate that the analyzed indole-based ligands exhibited affinity profiles comparable to the reference ligands (melatonin for 1DNU and febuxostat for 1N5X), thus suggesting a similar strength of binding to these domains. However, a notable distinction emerged concerning the 4COX protein domain. In this particular case, all of the ligands exhibited lower binding energies than the reference ligand (indomethacin), thus implying a lower affinity to this protein domain. Consequently, these ligands may potentially demonstrate inferior antioxidant properties compared to the reference ligand in the context of the 4COX domain.

## 3. Materials and Methods

### 3.1. Instrumentation and Chemicals

The synthesis reagents and solvents used in this study were commercially available. The IR spectra were obtained using FT/IR Nicolet iS5 (Thermo Scientific, Walthmam, MA, USA) (KBr pellet, cm^−1^). The ^1^H and ^13^C NMR spectra were obtained using Varian (Palo, Alto, CA, USA) VNMR-S 400 MHz (DMSO-*d*_6_ as the solvent and TMS as the internal standard). The melting points were measured using the SMP-20 apparatus (Büchi Labortechnik AG, Flawil, Switzerland). The EI mass spectra were obtained using the 320MS/450GC mass spectrometer (Bruker, Billerica, MA, USA). The nitrogen, carbon, hydrogen, and sulfur percentage content was determined through elemental analysis using the Elemental Analyzer Vario EL III apparatus (Shimadzu, Kyoto, Japan). TLC analysis was conducted using silica gel 60 plates with a fluorescent indicator (254 nm) and was then visualized under UV light (Sigma-Aldrich, Poznan, Poland).

### 3.2. Synthesis of Gramine Derivatives

A typical procedure for the synthesis of Compounds **2**–**9**

We used 2 mmol of the appropriate azole or benzazole (1,5-Dihydro-*2H*-imidazole-2-thione for **2** and **3**; imidazole-2-thione for **4**; 1-methyl-*1H*-imidazole-2-thione for **5**; 1,2-dihydro-*2H*-1,3-benzimidazole-2-thione for **6** and **7**; 3-methyl-*1H*-benzimidazole -2-thione for **8**; and 1,3-thiazolidine-2-thione for **9**. These were then diluted in 10 mL of EtOH and cooled to 0–5 °C. Then, a solution of NaOH (1.5 mmol) in 4 mL of H_2_O was added, and the mixture was stirred for 1 hour. After that, a solution of gramine (1 mmol) in 4 mL of EtOH was added, and the mixture was heated under reflux for 3–12 hours. The products obtained were filtered under a reduced pressure and washed with distilled water. Compounds **4**, **5**, and **9** were recrystallized from H_2_O (**4**) or toluene (**5**, **9**).

1-((1H-indol-3-yl)methyl)imidazolidine-2-thione (**2**)

White sold (99 mg, 43%); m.p 144–147 °C; ^1^H NMR (400 MHz, DMSO-*d*_6_): δ 11.02 (s, 1H), 8.06 (s, 1H), 7.76 (d, J = 7.9 Hz, 1H), 7.40–7.31 (m, 2H), 7.09 (ddd, J = 8.1, 6.9, 1.2 Hz, 1H), 6.98 (ddd, J = 8.0, 7.0, 1.1 Hz, 1H), 4.84 (s, 2H), 3.39 (ddd, J = 9.2, 6.8, 2.2 Hz, 2H), and 3.37–3.27 (m, 2H); ^13^C NMR (101 MHz, DMSO-*d*_6_): δ 181.83, 136.36, 126.62, 124.99, 121.28, 119.21, 118.68, 111.47, 109.71, 47.00, 41.35, and 40.58; IR (KBr, cm^−1^) ν_max_: 3317, 3210, 2887, 1502, 1454, 1251, 1225, 1071, 753, 644, and 599; and EI-MS (m/z, % int.): 231 (34). Analysis was calculated for C_12_H_13_N_3_S (MW = 231.32) with the following: C, 62.31; H, 5.66; N, 18.17; and S, 13.86; and found: C, 62.02; H, 5.67; N, 18.12; and S, 13.54.

1,3-bis((1H-indol-3-yl)methyl)imidazolidine-2-thione (**3**)

White solid (14 mg, 6%); m.p 157–160 °C; ^1^H NMR (400 MHz, DMSO-*d*_6_): δ 11.00 (d, J = 2.5 Hz, 2H), 7.78 (d, J = 7.9 Hz, 2H), 7.34 (dd, J = 5.4, 2.9 Hz, 4H), 7.07 (ddd, J = 8.1, 6.9, 1.2 Hz, 2H), 7.03–6.91 (m, 2H), 4.95 (s, 4H), and 3.22 (s, 4H); ^13^C NMR (101 MHz, DMSO-*d*_6_): 180.49, 136.36, 126.59, 125.00, 121.24, 119.26, 118.69, 111.42, 109.65, 44.56, and 42.44; IR (KBr, cm^−1^) ν_max_: 3397, 3057, 2910, 2881, 1502, 1455, 1328, 1254, 1094, 753, 635, and 593; and EI-MS (*m*/*z*, % int.): 360 (2). Analysis was calculated for C_21_H_20_N_4_S (MW = 360.48) with the following: C, 69.97; H, 5.59; N, 15.54; and S, 8.89; and found: C, 69.23; H, 5.55; N, 15.91; and S, 9.01.

1-((1H-indol-3-yl)methyl)-1,3-dihydro-2H-imidazole-2-thione (**4**)

Light brown crystals (124 mg, 54%); m.p 206–209 °C; ^1^H NMR (400 MHz, DMSO-*d*_6_): δ 12.07 (s, 1H), 11.09 (d, J = 7.9 Hz, 1H), 7.79–7.71 (m, 1H), 7.50 (dd, J = 6.0, 2.5 Hz, 1H), 7.37 (dt, J = 8.1, 0.9 Hz, 1H), 7.08 (dtd, J = 7.7, 6.7, 1.2 Hz, 1H), 6.96 (dddd, J = 9.1, 8.0, 7.0, 1.1 Hz, 1H), 6.93–6.80 (m, 2H), and 5.26 (s, 2H); ^13^C NMR (101 MHz, DMSO-*d*_6_): δ 160.40, 136.19, 126.20, 125.45, 121.41, 118.99, 118.85, 117.80, 114.35, 111.52, 110.24, and 40.96; IR (KBr, cm^−1^) ν_max_: 3221, 3116, 3029, 2916, 2713, 1551, 1470, 1263, 1138, 747, 616, and 576; and EI-MS (*m*/*z*, % int.): 229 (10). Analysis was calculated for C_12_H_11_N_3_S (MW = 229.30) with the following: C, 62.86; H, 4.84; N, 13.33; and S, 13.98; and found: C, 63,32; H, 5.12; N, 17.43; and S, 13.21.

1-((1H-indol-3-yl)methyl)-3-methyl-1,3-dihydro-2H-imidazole-2-thione (**5**)

White crystals (159 mg, 65%); m.p 166–168 °C; ^1^H NMR (400 MHz, DMSO-*d*_6_): δ 11.13–11.08 (m, 1H), 7.76–7.69 (m, 1H), 7.51 (d, J = 2.4 Hz, 1H), 7.37 (dt, J = 8.1, 0.9 Hz, 1H), 7.08 (ddd, J = 8.2, 7.0, 1.2 Hz, 1H), 7.04 (d, J = 2.4 Hz, 1H), 7.00–6.95 (m, 2H), 5.31–5.27 (m, 2H), and 3.48 (s, 3H); ^13^C NMR (101 MHz, DMSO-*d*_6_): δ 161.12, 136.17, 126.18, 125.59, 121.41, 118.91, 118.88, 118.39, 116.61, 111.54, 109.98, 42.09, and 34.38; IR (KBr, cm^−1^) ν_max_: 3427, 3212, 3162, 3132, 2914, 1554, 1459, 1223, 1139, 756, 664, and 599; and EI-MS (m/z, % int.): 245 (8). Analysis was calculated for C_13_H_15_N_3_S (MW = 245.34) with the following: C, 63.64; H, 6.16; N, 17.13; and S, 13.07; and found: C, 64.30; H, 6.18; N, 17.32; and S, 13.25.

1-((1H-indol-3-yl)methyl)-1,3-dihydro-2H-benzo[d]imidazole-2-thione (**6**)

Beige crystals (142 mg, 51%); m.p 244–242 °C; ^1^H NMR (400 MHz, DMSO-*d*_6_): δ 12.81 (s, 1H), 11.09 (d, J = 2.6 Hz, 1H), 7.95 (dd, J = 8.0, 1.1 Hz, 1H), 7.65 (d, J = 2.5 Hz, 1H), 7.46–7.37 (m, 1H), 7.33 (dt, J = 8.2, 1.0 Hz, 1H), 7.20–7.01 (m, 4H), 6.94 (ddd, J = 8.0, 7.0, 1.1 Hz, 1H), and 5.66 (s, 2H); ^13^C NMR (101 MHz, DMSO-*d*_6_): δ 168.10, 136.21, 132.26, 130.79, 126.11, 125.79, 122.74, 121.95, 121.36, 119.37, 118.77, 111.52, 110.31, 109.58, and 109.37; IR (KBr, cm^−1^) ν_max_: 3374, 3183, 3056, 1623, 1556, 1456, 1370, 1130, 737, 615, and 593; and EI-MS (*m*/*z*, % int.): 279 (39). Analysis was calculated for C_16_H_13_N_3_S (MW = 279.36) with the following: C, 68.79; H, 4.69; N, 15.04; and S, 11.48; and found: C, 68.62; H, 4.32; N, 15.91; and S, 10.57.

1,3-bis((1H-indol-3-yl)methyl)-1,3-dihydro-2H-benzo[d]imidazole-2-thione (**7**)

White crystals (53 mg, 13%); m.p 232–235 °C; ^1^H NMR (400 MHz, DMSO-*d*_6_): δ 11.10–11.05 (m, 2H), 7.98 (d, J = 8.0 Hz, 2H), 7.66 (d, J = 2.5 Hz, 2H), 7.43 (dq, J = 7.0, 4.0 Hz, 2H), 7.30 (d, J = 8.2 Hz, 2H), 7.04 (ddd, J = 11.2, 7.0, 2.3 Hz, 4H), 6.92 (ddd, J = 8.0, 7.0, 1.0 Hz, 2H), and 5.80 (s, 4H); ^13^C NMR (101 MHz, DMSO-*d*_6_): δ 168.43, 136.24, 131.39, 126.02, 125.79, 122.36, 121.35, 119.51, 118.81, 111.46, 110.33, 109.38, and 40.66; IR (KBr, cm^−1^) ν_max_: 3569, 3409, 2972, 2931, 1647, 1556, 1409, 1370, 1046, 777, 622, and 582; and EI-MS (m/z, % int.): 408 (4). Analysis was calculated for C_25_H_20_N_4_S (MW = 408.52) with the following: C, 73.50; H, 4.93; N, 13.71; and S, 7.85; and found: C, 73.90; H, 4.34; N, 13.55; and S, 7.64.

1-((1H-indol-3-yl)methyl)-3-methyl-1,3-dihydro-2H-benzo[d]imidazole-2-thione (**8**)

Beige crystals (132 mg, 50%); m.p 180–182 °C; ^1^H NMR (400 MHz, DMSO-*d*_6_): δ 11.12–11.07 (m, 1H), 7.92 (ddt, J = 7.9, 1.4, 0.7 Hz, 1H), 7.65 (d, J = 2.5 Hz, 1H), 7.54–7.47 (m, 1H), 7.43–7.37 (m, 1H), 7.33 (dt, J = 8.2, 0.9 Hz, 1H), 7.17 (pd, J = 7.4, 1.4 Hz, 2H), 7.05 (ddd, J = 8.2, 7.0, 1.2 Hz, 1H), 6.94 (ddd, J = 8.0, 7.0, 1.1 Hz, 1H), 5.75–5.68 (m, 2H), and 3.75 (s, 3H); ^13^C NMR (101 MHz, DMSO-*d*_6_): δ 168.83, 136.17, 132.15, 131.18, 126.10, 125.83, 122.65, 122.52, 121.35, 119.34, 118.79, 111.51, 110.22, 109.45, 109.34, 40.29, and 31.16; IR (KBr): IR (KBr, cm^−1^) ν_max_: 3454, 3228, 3062, 2930, 1549, 1486, 1408, 1338, 1129, 741, 623, and 598; and EI-MS (*m*/*z*, % int.): 293 (50). Analysis was calculated for C_17_H_15_N_3_S (MW = 293.39) with the following: C, 69,60; H, 5.15; N, 14.32; and S, 10.93; and found: C, 69.43; H, 5.16; N, 14.28; and S, 11.13.

3-((1H-indol-3-yl)methyl)thiazolidine-2-thione (**9**)

White solid (112 mg, 45%); m.p > 360 °C; ^1^H NMR (400 MHz, DMSO-*d*_6_): δ 11.16 (s, 1H), 7.69 (dt, J = 7.9, 1.0 Hz, 1H), 7.46 (s, 1H), 7.39 (dt, J = 8.1, 1.0 Hz, 1H), 7.11 (ddd, J = 8.2, 7.0, 1.2 Hz, 1H), 7.01 (ddd, J = 8.0, 7.0, 1.1 Hz, 1H), 5.07 (s, 2H), 3.93 (dd, J = 8.5, 7.5 Hz, 2H), and 3.20 (dd, J = 8.6, 7.6 Hz, 2H); ^13^C NMR (101 MHz, DMSO-*d*_6_): δ 194.00, 136.26, 126.32, 125.79, 121.49, 119.04, 118.90, 111.66, 108.45, 55.71, 43.73, and 26.33; IR (KBr, cm^−1^) ν_max_: 3257, 3113, 3040, 2918, 1550, 1490, 1429, 1313, 1216, 1125, 758, 679, 645, and 591; and EI-MS (m/z, % int.): 248 (36). Analysis was calculated for C_12_H_12_N_2_S_2_ (MW = 248.36) with the following: C,58.03; H, 4.87; N, 11.28; S, and 25.82; and found: C, 57.72; H, 4.76; N, 11.67; and S, 25.72.

A typical procedure for the synthesis of Compounds **10**–**13**

The gramine solution (1 mmol) and the corresponding substrate (1,3-thiazolidine-2-thione for **10**; 5-methyl-3H-1,3-benzoxazole-2-thione for **11**; and 1,2-dihydro-1,2,4-triazole-3-thione for **12** and **13**) (1 mmol) were heated under reflux for 5–10 hours in 8–10 mL of EtOH. Compounds **10** and **11** were crystallized, while **12** and **13** required purification through column chromatography (using CHCl_3_).

3-((1H-indol-3-yl)methyl)thiazole-2(3H)-thione (**10**)

Brown crystals (91 mg, 37%); m.p 149–151 °C; ^1^H NMR (400 MHz, DMSO-*d*_6_): δ 11.20 (s, 1H), 7.72 (dt, J = 7.9, 1.0 Hz, 1H), 7.57 (d, J = 2.6 Hz, 1H), 7.46 (d, J = 4.6 Hz, 1H), 7.39 (dt, J = 8.1, 0.9 Hz, 1H), 7.11 (ddd, J = 8.2, 7.0, 1.2 Hz, 1H), 7.01 (ddd, J = 8.1, 7.0, 1.1 Hz, 1H), 6.94 (d, J = 4.6 Hz, 1H), and 5.48 (s, 2H); ^13^C NMR (101 MHz, DMSO-*d*_6_): δ 185.59, 136.15, 132.55, 126.18, 126.00, 121.57, 119.14, 118.69, 111.68, 111.53, 109.14, and 44.06; IR (KBr, cm^−1^) ν_max_: 3260, 3132, 3103, 3058, 2917, 1543, 1456, 1258, 1193, 1128, 1042, 746, 631, and 580; and EI-MS (m/z, % int.): 246 (25). Analysis was calculated for C_12_H_10_N_2_S_2_ (MW = 246.35): C, 58.51; H, 4.09; N, 11.37; and S, 26.03; and found: C, 58.69; H, 3.56; N, 11.46; and S, 26.30.

3-((1H-indol-3-yl)methyl)-5-methylbenzo[d]oxazole-2(3H)-thione (**11**)

White crystals (227 mg, 77%); m.p 231–234 °C; ^1^H NMR (400 MHz, DMSO-*d*_6_): δ 11.22–11.16 (m, 1H), 7.85 (dq, J = 7.9, 0.9 Hz, 1H), 7.75 (d, J = 2.6 Hz, 1H), 7.43–7.31 (m, 3H), 7.12–7.04 (m, 2H), 6.99 (ddd, J = 8.0, 7.0, 1.1 Hz, 1H), 5.61 (s, 2H), and 2.33 (s, 3H); ^13^C NMR (101 MHz, DMSO-*d*_6_): δ 179.16, 144.60, 136.24, 134.58, 131.16, 126.43, 125.91, 124.84, 121.53, 119.04, 118.99, 111.69, 111.33, 109.60, 107.56, 41.72, and 20.92; IR (KBr, cm^−1^) ν_max_: 3316, 3057, 2926, 1556, 1407, 1357, 1219, 1131, 796, 620, and 597; and EI-MS (m/z, % int.): 294 (15). Analysis was calculated for C_17_H_14_N_2_OS (MW = 294.37) with the following: C, 69.36; H, 4.79; N, 9.52; and S, 10.89; and found: C, 69.14; H, 4.46; N, 9.50; and S, 10.96.

1-((1H-indol-3-yl)methyl)-1,2-dihydro-3H-1,2,4-triazole-3-thione (**12**)

Colorless oil (60 mg, 26%); ^1^H NMR (400 MHz, DMSO-*d*_6_): δ 12. 13.70 (s, 1H), 11.18 (s, 1H), 8.37 (s, 1H), 7.76–7.71 (m, 1H), 7.54 (d, J = 2.6 Hz, 1H), 7.39 (dt, J = 8.1, 0.9 Hz, 1H), 7.14–7.08 (m, 1H), 7.01 (ddd, J = 8.0, 7.0, 1.1 Hz, 1H), and 5.28 (s, 2H); ^13^C NMR (101 MHz, DMSO-*d*_6_): δ 165.67, 141.83, 136.20, 125.91, 121.58, 119.11, 118.58, 111.71, and 109.00; IR (KBr, cm^−1^) ν_max_: 3406, 3127, 3009, 2925, 1548, 1481, 1458, 1341, 1210, 1096, 745, 665, and 580; and EI-MS (m/z, % int.): 230 (25). Analysis was calculated for C_11_H_10_N_4_S (MW = 230.29) with the following: C, 57.37; H, 4.38; N, 24.33; and S, 13.92; and found: C, 57.39; H, 4.65; N, 24.42; and S, 13.72.

1,2-bis((1H-indol-3-yl)methyl)-1,2-dihydro-3H-1,2,4-triazole-3-thione (**13**)

Brown oil (36 mg, 10%); ^1^H NMR (400 MHz, DMSO-*d*_6_): δ 11.15 (s, 1H), 11.08–11.04 (m, 1H), 8.36 (s, 1H), 7.75 (dt, J = 7.9, 0.9 Hz, 1H), 7.71 (dq, J = 8.0, 0.8 Hz, 1H), 7.51 (d, J = 2.5 Hz, 1H), 7.43 (d, J = 2.5 Hz, 1H), 7.36 (dt, J = 8.1, 0.9 Hz, 1H), 7.33 (dt, J = 8.2, 0.9 Hz, 1H), 7.07 (dddd, J = 15.1, 8.2, 7.0, 1.2 Hz, 2H), 6.96 (dddd, J = 14.8, 7.9, 7.0, 1.0 Hz, 2H), 5.46 (s, 2H), and 5.32 (s, 2H); ^13^C NMR (101 MHz, DMSO-*d*_6_): δ 164.36, 140.51, 136.17, 136.08, 126.19, 125.88, 125.84, 125.53, 121.51, 121.29, 119.07, 119.00, 118.81, 118.54, 111.64, 111.46, 109.19, 108.79, 79.15, 43.90, and 40.55.; IR (KBr, cm^−1^) ν_max_: 3412, 2925, 1636, 1537, 1457, 1421, 1342, 1208, 1093, 746, and 578; and EI-MS (m/z, % int.): 359 (4). Analysis was calculated for C_20_H_17_N_5_S (MW = 359.45) with the following: C, 66.83; H, 4.77; N, 19.48; and S, 8.92; and found: C, 66.86; H, 4.53; N, 19.97; and S, 8.63.

Synthesis of (1-acetyl-1H-indol-3-yl)methyl pyrrolidine-1-carbodithioate (**15**) 

N-acetyl-3-acetoxymethylindole (0.5 mmol) and sodium pyrrolidinedithiocarbamate (1 mmol) were dissolved in water (10 mL) and heated under reflux for 6 h. The resulting mixture was then extracted with diethyl ether, washed with water and brine, dried over anhydrous KOH, and then evaporated to give a brown oil.

Brown oil (248 mg, 78%); ^1^H NMR (400 MHz, CDCl_3_): δ 7.69 (dd, J = 7.9, 1.2 Hz, 1H), 7.37–7.33 (m, 1H), 7.29 (d, J = 2.4 Hz, 1H), 7.20 (ddd, J = 8.2, 7.0, 1.3 Hz, 1H), 7.14 (ddd, J = 8.1, 7.0, 1.2 Hz, 1H), 4.78 (s, 2H), 3.95 (t, J = 6.8 Hz, 2H), 3.58 (t, J = 6.7 Hz, 2H), 2.60 (s, 3H), 2.03–2.00 (m, 2H), and 1.95 (td, J = 6.9, 1.6 Hz, 2H); ^13^C NMR (101 MHz, CDCl_3_): δ 192.97, 168.50, 136.10, 126.88, 125.48, 123.86, 122.33, 119.71, 119.03, 111.23, 54.71, 50.43, 32.80, 25.97, and 24.23; IR (KBr, cm^−1^) ν_max_: 3405, 2969, 2869, 1702, 1329, 1160, 1006, 954, and 743; and EI-MS (*m*/*z*, % int.): 318 (6). Analysis was calculated for C_16_H_18_N_2_OS_2_ (MW = 318.45) with the following: C, 60,35; H, 5.70; N, 8.80; and S, 20.13; and found: C, 61,01; H, 5.62; N, 8.72; and S, 20.42.

Synthesis of 3-(ethoxymethyl)-1H-indol-1-yl benzoate (**17**)

A mixture of benzoic acid (0.5 mmol), PPh_3_ (0.75 mmol), and NBS (0.75 mmol) in CH_2_Cl_2_ (2 mL) was prepared. The solution was stirred at 0 °C for 15 minutes and then warmed to room temperature. Next, 3-etoxymethylindole (0.55 mmol) and Et_3_N (0.55 mmol) were added, and the reaction mixture was stirred for one hour. The mixture was then diluted with EtOAc and washed with NaHCO_3_. The aqueous layer was extracted with EtOAc. The organic layers were combined, dried with anhydrous Na_2_SO_4_, and evaporated. The crude product obtained was purified by column chromatography (CHCl_3_: EtOAc 5:1).

Yellow oil (37 mg, 32%); ^1^H NMR (400 MHz, CDCl_3_): δ 8.18–8.13 (m, 1H), 8.05 (d, J = 7.1 Hz, 1H), 7.69–7.64 (m, 1H), 7.56–7.49 (m, 4H), 7.46–7.40 (m, 1H), 4.68 (s, 2H), 4.38 (q, J = 7.1 Hz, 2H), and 1.39 (t, J = 7.1 Hz, 3H); ^13^C NMR (101 MHz, CDCl_3_): δ 166.65, 162.35, 134.53, 132.79, 130.54, 129.50, 128.85, 128.79, 128.28, 121.21, 119.35, 117.48, 110.65, 60.94, and 14.30; FT-IR (KBr, cm^−1^) ν_max_: 3057, 2924, 1783, 1715, and 1618; and EI-MS (m/z, % int.): 295 (100%). Analysis was calculated for C_18_H_17_NO_3_ (MW = 295.12) with the following: C, 73.20; H, 5.80; N, 4.74; and O, 16.25; and found: C, 73.41; H, 6.12; N, 4.53; and O, 15.94%.

A typical procedure for the synthesis of Compounds **18**–**27**

Next, to 5 mL of anhydrous DMF, which was cooled to 0 °C, NaH (60%, 1 mmol) was added. The resulting mixture was stirred at 0 °C for 15 minutes. Then, 3-etoxymethylindole (1 mmol) that was dissolved in 1 mL of anhydrous DMF was added, and the mixture was stirred for 30 minutes at 0 °C. Finally, an appropriate bromoester (methyl bromoacetate, ethyl bromoacetate, isopropyl bromoacetate, *tert*-butyl bromoacetate, ethyl 2-bromopropionate, ethyl 2-bromovalerate, phenyl bromoacetate, benzyl bromoacetate, methyl α-bromophenyl acetate, and ethyl α-bromophenyl acetate) (1 mmol) were added dropwise and stirred for 24 h at room temperature. The resulting mixture was then extracted with EtOAc, washed with water and brine, dried over anhydrous Na_2_SO_4_, and evaporated. The resulting crude product was purified using column chromatography with gradient elution, starting from PhMe: EtOAc 50:1.

Methyl 2-(3-(ethoxymethyl)-1H-indol-1-yl)acetate (**18**)

Yellow oil (94 mg, 38%); ^1^H NMR (400 MHz, CDCl_3_): δ 7.71 (dt, J = 7.8, 1.0 Hz, 1H), 7.22 (d, *J* = 1.7 Hz, 1H), 7.17–7.15 (m, 2H), 7.08 (s, 1H), 4.81 (s, 2H), 4.71 (d, J = 0.7 Hz, 2H), 3.73 (s, 3H), 3.57 (q, *J* = 7.0 Hz, 2H), 1.23 (t, *J* = 7.0 Hz, 3H); ^13^C NMR (101 MHz, CDCl_3_): δ 168.95, 136.93, 127.77, 127.63, 122.36, 119.89, 119.63, 113.50, 108.89, 65.17, 64.38, 52.50, 47.55, 15.27; FT-IR (KBr, cm^−1^) ν_max_: 3050, 2952, 2866, 1743, 1661, 1614; and EI-MS (*m*/*z*, % int.): 246 (15). Analysis was calculated for C_14_H_17_NO_3_ (MW = 247.12) with the following: C, 68.00; H, 6.93; N, 5.66; and O, 19.41; and found: C, 67.71; H, 7.43; N, 5.82; and O, 19.04%.

Ethyl 2-(3-(ethoxymethyl)-1H-indol-1-yl)acetate (**19**)

Yellow oil (144 mg, 55%); ^1^H NMR (400 MHz, CDCl_3_): δ 7.71 (d, *J* = 7.8 Hz, 1H), 7.23 (dd, *J* = 2.5, 1.0 Hz, 1H), 7.22 (d, *J* = 1.0 Hz, 1H), 7.17–7.13 (m, 1H), 7.09 (s, 1H), 4.79 (s, 2H), 4.71 (s, 2H), 4.20 (q, *J* = 7.2 Hz, 2H), 3.55 (t, *J* = 7.0 Hz, 2H), and 1.27–1.21 (m, 6H); ^13^C NMR (101 MHz, CDCl_3_): δ 168.45, 136.97, 127.78, 127.71, 122.29, 119.83, 119.60, 113.39, 108.93, 65.10, 64.39, 61.63, 47.72, 15.27, and 14.09; FT-IR (KBr, cm^−1^) ν_max_: 3053, 2978, 2932, 2867, 1750, and 1614; and EI-MS (*m*/*z*, % int.): 261 (20). Analysis was calculated for C_15_H_19_NO_3_ (MW = 261.14) with the following: C, 68.94; H, 7.33; N, 5.36; and O, 18.37; and found: C, 69.00; H, 7.15; N, 5.41; and O, 18.41%.

Isopropyl 2-(3-(ethoxymethyl)-1H-indol-1-yl)acetate (**20**)

Yellow oil (165 mg, 60%); ^1^H NMR (400 MHz, CDCl_3_): δ 7.71 (dt, *J* = 7.8, 1.0 Hz, 1H), 7.23 (dd, *J* = 3.0, 1.0 Hz, 1H), 7.22 (d, *J* = 1.0 Hz, 1H), 7.17–7.13 (m, 1H), 7.09 (s, 1H), 5.06 (hept, *J* = 6.3 Hz, 1H), 4.76 (s, 2H), 4.71 (d, *J* = 0.7 Hz, 2H), 3.56 (q, *J* = 7.0 Hz, 2H), and 1.24–1.21 (m, 9H); ^13^C NMR (101 MHz, CDCl_3_): δ 167.98, 137.00, 127.81, 122.25, 122.14, 119.80, 119.59, 113.30, 108.96, 69.49, 65.04, 64.39, 47.95, 21.69, and 15.28; FT-IR (KBr, cm^−1^) ν_max_: 3056, 2979, 2933, 2859, 1737, and 1615; and EI-MS (*m*/*z*, % int.): 275 (12,5). Analysis was calculated for C_16_H_21_NO_3_ (MW = 275.15) with the following: C, 69.79; H, 7.69; N, 5.09; and O, 17.43; and found: C, 70.00; H, 7.51; N, 5.18; and O, 17.31%. 

*Tert*-butyl 2-(3-(ethoxymethyl)-1H-indol-1-yl)acetate (**21**)

Yellow oil (191 mg, 66%); ^1^H NMR (400 MHz, CDCl_3_): δ 7.71 (dt, J = 7.9, 1.0 Hz, 1H), 7.23–7.22 (m, 2H), 7.16 –7.12 (m, 1H), 7.09 (t, J = 0.8 Hz, 1H), 4.71 (d, J = 0.7 Hz, 2H), 4.70 (s, 2H), 3.55 (q, J = 7.0 Hz, 2H), 1.48 (s, 1H), 1.44 (s, 9H), and 1.22 (t, J = 7.0 Hz, 3H); ^13^C NMR (101 MHz, CDCl_3_): δ 167.59, 137.00, 127.86, 127.81, 122.19, 119.73, 119.57, 113.16, 108.97, 82.51, 64.99, 64.40, 48.50, 27.95, and 15.28; FT-IR (KBr, cm^−1^) ν_max_: 3054, 2979, 2933, 2873, 1745, and 1614; and EI-MS (m/z, % int.): 289 (23). Analysis was calculated for C_17_H_23_NO_3_ (MW = 289.17) with the following: C, 70.56; H, 8.01; N, 4.84; and O, 16.59; and found: C, 70.73; H, 7.94; N, 5.03; and O, 16.30%.

Ethyl 2-(3-(ethoxymethyl)-1H-indol-1-yl)propanoate (**22**)

Yellow oil (157 mg, 57%); ^1^H NMR (400 MHz, CDCl_3_): δ 7.71 (d, J = 8.3 Hz, 1H), 7.31–7.27 (m, 2H), 7.24–7.20 (m, 1H), 7.16–7.13 (m, 1H), 5.10 (q, J = 7.3 Hz, 1H), 4.71 (s, 2H), 4.15 (qd, J = 7.1, 1.0 Hz, 2H), 3.57 (q, J = 7.0 Hz, 2H), 1.79 (d, J = 7.3 Hz, 3H), and 1.22 (dt, J = 13.2, 7.1 Hz, 6H); ^13^C NMR (101 MHz, CDCl_3_): δ 171.21, 136.62, 127.81, 124.45, 122.05, 119.84, 119.58, 113.26, 109.15, 65.16, 64.58, 61.57, 53.50, 17.56, 15.28, and 14.04; FT-IR (KBr, cm^−1^) ν_max_: 3051, 2977, 2936, 2860, 1739, and 1614; and EI-MS (m/z, % int.): 275 (50). Analysis was calculated for C_16_H_21_NO_3_ (MW = 275.15) with the following: C, 69.79; H, 7.69; N, 5.09; O, and 17.43; and found: C, 69.76; H, 7.99; N, 4.92; and O, 17.33%.

Ethyl 2-(3-(ethoxymethyl)-1H-indol-1-yl)pentanoate (**23**)

Yellow oil (222 mg, 73%); ^1^H NMR (400 MHz, CDCl_3_): δ 7.71 (d, J = 7.9 Hz, 1H), 7.33 (d, J = 8.3 Hz, 1H), 7.29 (s, 1H), 7.24–7.20 (m, 1H), 7.16–7.12 (m, 1H), 4.96 (dd, J = 9.3, 6.2 Hz, 1H), 4.72 (d, J = 0.7 Hz, 2H), 4.15 (qd, J = 7.1, 2.9 Hz, 2H), 3.56 (q, J = 7.0 Hz, 2H), 1.30 (t, J = 7.1 Hz, 3H), 1.25–1.20 (m, 6H), and 0.97–0.91 (m, 4H); ^13^C NMR (101 MHz, CDCl_3_): δ 170.91, 137.01, 127.68, 124.81, 122.00, 119.77, 119.55, 113.30, 109.13, 65.07, 64.62, 61.46, 57.82, 34.04, 19.24, 15.28, 14.07, and 13.52; FT-IR (KBr, cm^−1^) ν_max_: 3051, 2964, 2932, 2873, 1740, and 1614; and EI-MS (*m*/*z*, % int.): 303 (45). Analysis was calculated for C_18_H_25_NO_3_ (MW = 303.18) with the following: C, 71.26; H, 8.31; N, 4.62; and O, 15.82; and found: C, 70.98; H, 8.42; N, 4.60; and O, 16.00%. 

Phenyl 2-(3-(ethoxymethyl)-1H-indol-1-yl)acetate (**24**)

Yellow oil (114 mg, 37%); ^1^H NMR (400 MHz, CDCl_3_): δ 8.44 (d, J = 8.1 Hz, 1H), 7.63 (d, J = 7.0 Hz, 1H), 7.54 (s, 1H), 7.40–7.36 (m, 1H), 7.34–7.29 (m, 3H), 7.03–6.99 (m, 3H), 5.15 (s, 2H), 4.66 (d, J = 1.1 Hz, 2H), 3.61 (q, J = 7.0 Hz, 2H), and 1.27 (t, J = 7.0 Hz, 3H); ^13^C NMR (101 MHz, CDCl_3_): δ 166.17, 157.54, 136.18, 129.70, 129.25, 125.75, 124.22, 122.11, 121.83, 121.20, 119.46, 116.67, 114.69, 67.78, 66.03, 64.33, and 15.21; FT-IR (KBr, cm^−1^) ν_max_: 3119, 3059, 2969, 2940, 2842, 1707, and 1600; and EI-MS (m/z, % int.): 309 (35). Analysis was calculated for C_19_H_19_NO_3_ (MW = 309.14) with the following: C, 73.77; H, 6.19; N, 4.53; and O, 15.52; and found: C, 73.80; H, 6.27; N, 4.49; and O, 15.43%.

Benzyl 2-(3-(ethoxymethyl)-1H-indol-1-yl)acetate (**25**)

Yellow oil (243 mg, 75%); ^1^H NMR (400 MHz, CDCl_3_): δ 7.71 (d, J = 7.8 Hz, 1H), 7.34–7.32 (m, 3H), 7.27 (d, J = 4.0 Hz, 2H), 7.21 (dd, J = 6.2, 1.3 Hz, 2H), 7.17–7.13 (m, 1H), 7.08 (s, 1H), 5.16 (s, 2H), 4.84 (s, 2H), 4.70 (d, J = 0.7 Hz, 2H), 3.55 (q, J = 7.0 Hz, 2H), and 1.22 (t, J = 7.0 Hz, 3H); ^13^C NMR (101 MHz, CDCl_3_): δ 168.31, 136.98, 135.02, 128.57, 128.48, 128.27, 127.83, 127.68, 122.36, 119.90, 119.65, 113.55, 108.96, 67.23, 65.07, 64.37, 47.71, and 15.27; FT-IR (KBr, cm^−1^) ν_max_: 3033, 2974, 2874, 2840, 1747, and 1683; and EI-MS (*m*/*z*, % int.): 323 (23). Analysis was calculated for C_20_H_21_NO_3_ (MW = 323.15) with the following: C, 74.28; H, 6.55; N, 4.33; and O, 14.84; and found: C, 74.27; H, 6.74; N, 3.99; and O, 15.00%.

Methyl 2-(3-(ethoxymethyl)-1H-indol-1-yl)-2-phenylacetate (**26**)

Yellow oil (133 mg, 41%); ^1^H NMR (400 MHz, CDCl_3_): δ 7.72 (d, J = 7.3 Hz, 1H), 7.38 (d, J = 2.3 Hz, 3H), 7.19–7.17 (m, 3H), 7.16–7.13 (m, 1H), 7.10 (s, 1H), 7.10–7.08 (m, 1H), 4.65 (t, J = 0.7 Hz, 2H), 3.83 (s, 3H), 3.81 (s, 1H), 3.56–3.50 (m, 3H), and 1.20 (t, J = 7.0 Hz, 3H); ^13^C NMR (101 MHz, CDCl_3_): δ 170.01, 138.70, 129.63, 129.06, 128.98, 128.11, 127.92, 127.73, 125.94, 122.24, 120.15, 119.75, 113.32, 109.00, 65.14, 64.57, 61.85, 52.71, and 15.25; FT-IR (KBr, cm^−1^) ν_max_: 3033, 2952, 2873, 1734, and 1612; and EI-MS (*m*/*z*, % int.): 323 (5). Analysis was calculated for C_20_H_21_NO_3_ (MW = 323.15) with the following: C, 74.28; H, 6.55; N, 4.33; and O, 14.84; and found: C, 74.22; H, 6.69; N, 4.12; and O, 14.97%.

Ethyl 2-(3-(ethoxymethyl)-1H-indol-1-yl)-2-phenylacetate (**27**)

Yellow oil (109 mg, 32%); ^1^H NMR (400 MHz, CDCl_3_): δ 7.72 (d, J = 7.8 Hz, 1H), 7.38–7.34 (m, 4H), 7.23–7.21 (m, 1H), 7.18–7.14 (m, 3H), 7.11 (d, J = 4.0 Hz, 1H), 6.19 (s, 1H), 4.66 (s, 2H), 4.31–4.26 (m, 2H), 3.53 (qd, J = 7.0, 0.9 Hz, 2H), 1.26 (t, J = 7.1 Hz, 4H), and 1.20 (t, J = 7.0 Hz, 3H); ^13^C NMR (101 MHz, CDCl_3_): δ 169.50, 137.03, 134.57, 129.68, 129.02, 128.09, 127.53, 126.04, 122.17, 120.10, 119.73, 113.20, 109.05, 65.07, 64.57, 61.94, 15.24, 14.07, and 13.97; FT-IR (KBr, cm^−1^) ν_max_: 3058, 2978, 2935, 2864, 1745, and 1613; and EI-MS (*m*/*z*, % int.): 337 (40). Analysis was calculated for C_21_H_23_NO_3_ (MW = 337.17) with the following: C, 74.75; H, 6.87; N, 4.15; and O, 14.23; and found: C, 74.93; H, 6.59; N, 4.32; and O, 14.16%. 

Synthesis of (1-acetyl-1H-indol-3-yl)methyl 2,5-dihydroxybenzoate (**29**)

*N*-acetyl-3-hydroxymethylindole (0.72 mmol) and 2,5-dihydroxybenzoic acid (0.72 mmol) were dissolved in THF (5 mL), then DCC (0.72 mmol) was added, and the mixture was then stirred for 48 hours at room temperature. The resulting white precipitate was filtered, and the filtrate was evaporated so as to obtain a dark brown precipitate. The precipitate was then dissolved in EtOAc, washed with 5% citric acid, saturated with NaHCO_3_ and brine, and then dried over anhydrous Na_2_SO_4_ and evaporated. The crude product was purified by column chromatography (PhMe: EtOAc 5:1).

Orange oil (87 mg, 37%)^; 1^H NMR (400 MHz, CDCl_3_): δ 8.41 (d, J = 8.4 Hz, 1H), 7.65 (d, J = 7.1 Hz, 1H), 7.54 (s, 1H), 7.39–7.27 (m, 3H), 7.18–7.16 (m, 2H), 5.50 (s, 2H), and 2.64 (s, 3H); ^13^C NMR (101 MHz, CDCl_3_): δ 169.60, 168.88, 155.71, 148.06, 135.84, 129.00, 128.19, 125.78, 125.26, 124.40, 124.05, 119.04, 118.48, 116.78, 114.60, 111.90, 58.72, and 23.92; FT-IR (KBr, cm^−1^) ν_max_: 3215, 2931, 2854, 1733, 1686, and 1620; and EI-MS (m/z, % int.): 325 (10). Analysis was calculated for C_18_H_15_NO_5_ (MW = 325.10) with the following: C, 66.46; H, 4.65; N, 4.31; and O, 24.59; and found: C, 66.51; H, 9.58; N, 4.43; and O, 24.89%.

### 3.3. X-ray Analysis

Single-crystal X-ray diffraction measurements were carried out with the monochromated CuKα radiation on a SuperNova diffractometer (**4**, **5**, **7**, **8**, **9**, and **11**), or with MoKα radiation on an Xcalibur diffractometer (**2** and **10**). Each dataset was measured with an omega scan. These data were processed with the CrysAlisPro 1.171.42 software [76]. The crystal structures were solved by direct methods with SHELXT [77] and refined by full-matrix least-squares calculations on F^2^ with SHELXL [78]. All non-H atoms were refined with anisotropic displacement parameters. Hydrogen atoms bonded to C and N atoms were placed at calculated positions based on the environment and perceived hybridization of the atoms to which they were bonded. For room temperature structures, the methyl, methylene, and aromatic C-H distances were standardized to 0.96, 0.97, and 0.93 Å, respectively, and the N-H distance was 0.86 Å. For low-temperature structures, the corresponding values were 0.98 and 0.99 and 0.95 Å and 0.88 Å. The solvent hydrogen atoms were located on difference Fourier maps, and their O-H distances were standardized to the values of 0.82 and 0.85 Å for the ethanol and water molecules, respectively. All H-atoms were refined as ‘riding’ on their carriers. During the refinement, isotropic displacement parameters for H-atoms were assigned as 20% higher than the isotropic equivalent for the atom to which the H-atom was bonded. The crystals of **8** were inversion-twinned with a ratio of 0.70(3):0.30(3). Moreover, in the crystal of **9**, there were signs of disorder in the thiazolidine moiety. We modeled this disorder by taking into account two alternative positions for one of the two methylene groups for which the component occupancy factors were refined to 0.62(3) and 0.38(3). MERCURY [79] was used to prepare drawings. CCDC contains the supplementary crystallographic data for **2** (Deposition Number 2346551), **4** (Deposition Number 2346552), **5** (Deposition Number 2346553), **7** (Deposition Number 2346554), **8** (Deposition Number 2346555), **9** (Deposition Number 2346556), **10** (Deposition Number 2346557), and **11** (Deposition Number 2346558). These data can be obtained free of charge via http://www.ccdc.cam.ac.uk/conts/retrieving.html (accessed on 8 April 2024) (or from the CCDC, 12 Union Road, Cambridge CB2 1EZ, UK; Fax: +44 1223 336033; email: deposit@ccdc.cam.ac.uk).

### 3.4. Biological Study 

#### 3.4.1. Antibacterial and Antifungal Activity Measurements

The antibacterial properties of the compounds were determined against selected bacteria: *Micrococcus luteus*, *Bacillus subtilis*, *Escherichia coli*, and *Pseudomonas fluorescens*. The antifungal activity of the compounds was determined against *Alternaria alternata*, *Fusarium culmorum*, *Trichoderma harzianum*, *Trichoderma harzianum*, and *Botrytis cinerea*. All the cultures of microorganisms were obtained from the pure culture collection of the Microbiology Department of the Faculty of Soil Science and Microbiology of the Poznan University of Life Sciences. 

The well diffusion method was used to evaluate the antimicrobial properties of the compounds. A broth medium was used for the bacterial tests, while potato dextrose agar (PDA) was used for mold cultivation. Next, 6 mL of each liquidized medium was poured into sterile Petri dishes and allowed to solidify. After this, two 0.5 cm-diameter sterile glass rings were placed on the surface of each plate. Then, 20 mL of each liquid medium containing suspensions of the tested microorganisms was added. The final bacterial suspension had a density of 107 cells/cm^3^, which was obtained from 48-hour cultures on broth slants, and the fungal suspension had a density of 108 spores/cm^3^, which was obtained from 5-day cultures on PDA slants. After the medium solidified, the glass rings were removed with a pencil, leaving two wells on each plate. Then, 0.1 mL of the compound dissolved in pure dimethyl sulfoxide was added to one well, and 0.1 mL of pure dimethyl sulfoxide was added to the other well, which served as a control. Each compound was tested in four replicates. Plates were incubated in a thermostat at 27 °C for *M. luteus*, *B. subtilis*, and *P. fluorescens* cultures, as well as at 37 °C for the *E. coli* culture for 48 hours. All fungal cultures were incubated in a thermostat at 24 °C for five days. At the end of the incubation, the growth inhibition diameters of the tested strains were measured using calipers.

#### 3.4.2. Human Red Blood Cell (RBC) Preparation

Human RBC suspensions (~65% hematocrit) were purchased from the Blood Bank in Poznań according to the bilateral agreement between the Adam Mickiewicz University and Blood Bank no. ZP/2867/D/21 without any contact with blood donors. The RBCs were washed three times (960× *g*, 10 min, 4 °C) in 7.4 pH phosphate-buffered saline (PBS_137 mM of NaCl, 2.7 mM of KCl, 10 mM of Na_2_HPO_4_, and 1.76 mM of KH_2_PO_4_), which was supplemented with 10 mM of glucose. After washing, the RBCs were suspended in the PBS buffer at 1.65 × 10^9^ cells/mL, stored at 4 °C, and used within 5 h.

#### 3.4.3. Inhibition of Free Radical-Induced Hemolysis

The cytoprotective activity of the derivatives was evaluated in accordance with a previously described method [14,15,16]. Briefly, human RBCs (1.65 × 10^8^ cells/mL, 1.5% hematocrit) were preincubated in PBS (pH 7.4) supplemented with 10 mM of glucose, which contained the tested compound or Trolox used as a standard antioxidant at a concentration of 0.01 mg/mL for 20 min at 37 °C in a shaking incubator. After preincubation, 2,2′-azobis(2-methylpropionamidine) dihydrochloride (AAPH) was added to a final concentration of 60 mM, and samples were incubated for the next four hours at 37 °C. RBCs incubated in PBS and in PBS with AAPH were used as negative and positive controls, respectively. After incubation, the RBC suspensions were centrifuged (960× *g*, 5 min, + 4 °C), and the degree of hemolysis was determined by measuring the absorbance (Ab) of the supernatant at λ = 540 nm in a BioMate™ 160 UV–Vis spectrophotometer. The percentage of free radical-induced hemolysis inhibition was calculated using the following equation:

inhibition of hemolysis (%) = 100 − [(Ab_comp_/Ab_AAPH_) × 100],

where Ab_comp_ is the absorbance value of the supernatants obtained from samples incubated with a compound tested in the presence of AAPH and Ab_AAPH_ is the absorbance of the supernatant obtained from the positive control, respectively. Each sample was prepared in triplicate and results were expressed as the mean ±SD value from three independent experiments (n = 6), using RBCs obtained from different donors. 

#### 3.4.4. Ferrous Ion (Fe^2+^) Chelating Assay 

The ferrous ions’ chelating activity of the derivatives was evaluated in accordance with a previously described method [15]. The Fe^2+^-chelating ability of the tested compounds was determined by the absorbance of the ferrous-ion–ferrozine complex at 562 nm at room temperature (~22 °C, RT). Briefly, 0.1 mg/mL of the concentration of the tested compounds in 0.2 mL of ethyl alcohol was added to a solution of 0.6 mM of FeCl_2_ (0.05 mL). EDTA was used as the standard metal chelator. The reaction was started by adding 5 mM of ferrozine (0.05 mL) in ethyl alcohol and then immediately shaking vigorously. The samples were stored for 10 min at room temperature. After incubation, the absorbance (Ab) of the solutions was measured at 562 nm in a BioMate™ 160 UV–Vis spectrophotometer. The percentage of inhibition of the ferrozine–Fe^2+^ complex formation was calculated using the following equation:

Fe^2+^ chelating (%) = [1 − (Ab1/Ab0)] × 100,

where Ab1 is the absorbance in the presence of the compound tested or EDTA and Ab0 is the absorbance of the sample without the tested compound. Each sample was made in triplicate and three independent experiments were performed (n = 9).

#### 3.4.5. Hemolysis Assay under Physiological Condition 

The hemolytic activity was evaluated according to the previously described method [14,15,16]. Briefly, RBCs (1.65 × 10^8^ cells/mL, 1.5% hematocrit) were incubated in a PBS (pH = 7.4) supplemented with 10 mM of glucose (Sigma Aldrich, Steinheim, Germany) and containing the tested compound at the concentration of 0.1 mg/mL for 60 min at 37 °C in a thermo shaker (BioSan Thermo-Shaker TS-100C, Biosan, Riga, Latvia). The negative control sample was a solution with RBCs incubated in PBS without the addition of the tested compounds. The positive control sample was a solution of the RBCs incubated in deionized water without the addition of the tested compounds. Each sample was prepared in triplicate, and the experiments were repeated three times with RBCs from different donors. After incubation, the RBC suspensions were centrifuged (Sigma 3–30 K Sartorious AG, Göttingen, Germany) (960× *g*, 10 min, 4 °C), and the degree of hemolysis was estimated by measuring the absorbance of the supernatant on a BioMate™ 160 UV-Vis spectrophotometer (Thermo Scientific, Waltham, MA, USA) at 540 nm. The results were expressed as the percentage (%) of hemolysis, which was calculated using the following formula:

hemolysis (%) = (sample Ab/positive control Ab) × 100,

where sample Ab is the absorbance value of the supernatant of RBCs incubated with the tested compounds and the negative control, and the positive control AB is an absorbance value of the supernatant of RBCs incubated in ice-cold deionized water. Each sample was prepared in triplicate, and the results are presented as a mean value (±SD) of the three independent experiments (*n* = 9).

#### 3.4.6. Statistical Analysis

For the antioxidant and cytoprotective properties, data were plotted as the mean value ± standard deviation (SD) of the results of three independent experiments, with every sample taken in triplicate (n = 9). A paired t-Student test was used to, respectively, compare the derivatives’ activity with the activity of the standard Trolox or EDTA. Statistical significance was defined as *p* < 0.05. Inactive compounds were indicated as n.a. Non statistically significant difference is indicated as n.s.

### 3.5. In Silico Study

The physicochemical calculations were conducted using the SwissADME website: www.swissadme.ch (accessed on 2 February 2024).

### 3.6. Molecular Docking

The molecular docking process commenced by converting the SMILES representation of indole-based chemical structures into 3D structures, and this was accomplished through the application of OpenBabel tool version 3.1.1 [80,81]. Subsequently, the protein domains corresponding to PDB [82], IDs 1DNU [83], 1N5X [84], and 4COX [85] were prepared in accordance with the standard AutoDock tool 1.5.7 scheme [86]. Molecular dockings were then carried out using AutoDock Vina [87], with the specific parameters outlined in Table 5 for each docking search.

## 4. Conclusions

The newly synthesized indole derivatives with methylene-bridged azole and benzazole substituents at C3 are compounds with a strong cytoprotective activity under oxidative stress conditions. The exceptions are derivatives appearing as zwitterions at a physiological pH, which occurred because of their ability to convert to harmful thiyl radicals. Of the two possible tautomeric forms, the molecules in crystals and those in a DMSO-*d*_6_ solution appeared as the thione tautomers. The C=S group was found to be the primary site for H-bonding in the condensed media: it has the ability to form multicenter hydrogen bonds with N-H and C-H donors. 

Compared to imidazoles and oxazoles, thiazoles are more prone toward conformational changes driven by hydrogen bond association. The steric effects witnessed in the crystals of **7** and **8** (which are also supposedly present in Compounds **3** and **13**) accounted for their high hemolytic activity.

Since the majority of the indole derivatives substituted solely at the C3 position are hemocompatibile, and as all of them adhere to the Lipinski and Veber rules, they represent promising candidates for future research on designing new bioactive compounds and drugs. The results presented in this study may facilitate the development of novel indole-based molecules with antioxidant and cytoprotective activities.

## Figures and Tables

**Table 1:** Torsion angles (◦) describing the rotation around the methylene C-C and C-N bonds in the molecules present in crystals. Molecular diagrams and perspective views of the molecules are provided to relate the metrical values to a particular molecular shape.

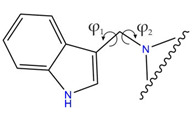			
	φ_1_	φ_2_	
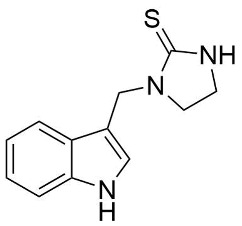 **2**	−77.7(5)	89.6(5)	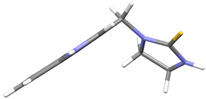
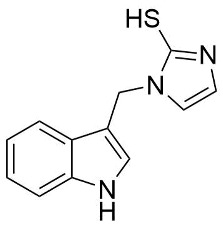 **4**	−74.6(2)	−57.9(2)	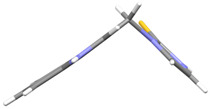
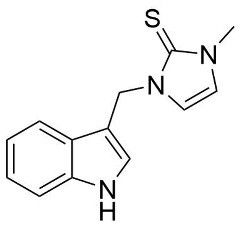 **5**	−79.2(3)	−58.3(3)	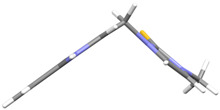
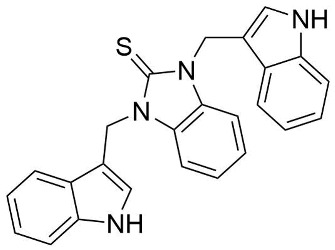 **7**	−69.1(3)−67.9(3)	−63.6(2)−66.6(2)	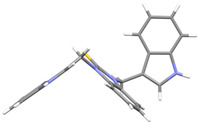
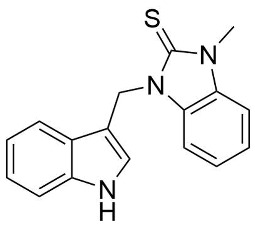 **8**	−84.3(3)	−74.8(3)	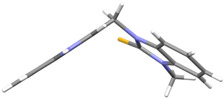
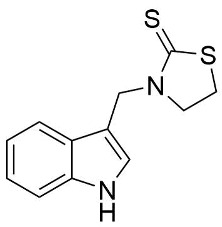 **9**	−79.1(2)	−62.0(2)	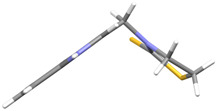
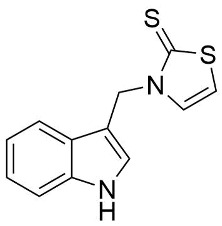 **10**	−83.23(19)	34.4(2)	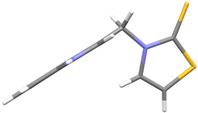
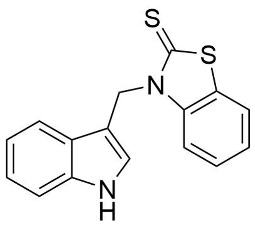 **10, from ref. [16]**	−165.4(2)	76.1(3)	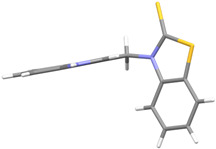
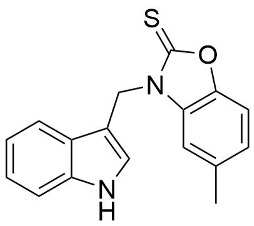 **11**	−51.6(2)	−63.64(19)	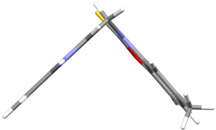
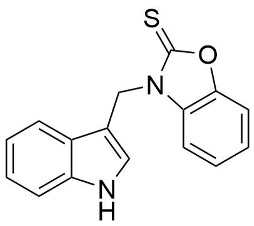 **11, from ref. [16]**	−88.96(16)	−80.65(16)	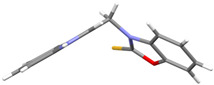

**Table 2:** Fungicidal activities of Compounds **1**–**29**. Growth inhibition zones: <9 mm—low active compounds; 10–15 mm—medium active compounds; and >15 mm—active compounds.

Compound	Zone of Growth Inhibition [mm]				
	*Alternaria alternata*	*Fusarium culmorum*	*Trichoderma harzianum*	*Trichoderma atroviride*	*Botrytis cinerea*
**Gramine (1)**	10.0	3.5	0	13.0	11.2
**2**	2.3	2.8	8.0	2.5	3.5
**3**	9	1	4	11	16
**4**	2.0	3.5	7.5	1.5	4.3
**5**	13.0	4.5	7.0	4.0	4.5
**6**	2.3	7.8	13.0	22.0	3.3
**7**	4.8	5.5	2.8	13.5	2.3
**8**	2.3	4.0	9.0	12.0	5.0
**9**	2.0	2.5	3.7	3.0	4.0
**10**	9	2.1	8	5	23
**11**	9	1	3.5	0	17
**12**	10	1	8.4	6.6	19
**13**	18	13.2	4.5	7	16.5
**17**	1.3	3.8	5.0	20.0	4.0
**18**	0	3.0	7.5	10.0	3.8
**19**	8.0	4.5	6.5	11.5	3.5
**20**	0	5.0	8.0	10.5	3.5
**21**	10.0	1.5	0	3.0	18.0
**22**	3.5	4.5	8.5	11.0	4.5
**23**	1.5	3.8	9.5	10.0	4.5
**24**	7.0	1.5	2.0	0	11.0
**25**	6.0	2.0	0	8.0	15.0
**26**	2.8	5.0	20.0	20.0	4.0
**27**	0	4.0	9.0	2.0	3.8
**29**	8.5	3.8	6.0	15.0	5.3

**Table 3:** The physicochemical, pharmacokinetic, and drug-likeness properties of the indole derivatives. LogS in the table is the average value of the logS calculated using three different methods. * Solubility class—logS scale: insoluble < −10, poorly < −6, moderately < −4, soluble < −2, and very < 0.

Compound	MW [g/mol]	logP	HBD	HBA	RTB	TPSA [Å^2^]	GI Absorption	BBB Permeant	LogS	Solubility *
2	231.32	1.80	2	0	2	63.15	High	Yes	−3.01	Soluble
3	360.48	3.39	2	0	4	70.15	High	No	−5.32	Moderately
4	229.30	2.17	1	1	2	72.41	High	Yes	−3.63	Soluble
5	245.34	1.94	1	0	2	61.07	High	Yes	−3.32	Soluble
6	279.36	3.25	1	1	2	72.41	High	Yes	−5.08	Moderately
7	408.52	4.78	2	0	4	73.53	High	Yes	−6.91	Poorly
8	293.39	3.43	1	0	2	57.74	High	Yes	−4.58	Moderately
9	248.37	2.63	1	0	2	76.42	High	Yes	−3.77	Soluble
10	246.35	3.03	1	0	2	70.15	High	No	−3.99	Soluble
11	294.37	3.90	1	1	2	65.95	High	Yes	−5.34	Moderately
12	230.29	1.85	1	2	2	85.30	High	No	−5.47	Moderately
13	359.45	3.38	2	1	4	86.42	High	Yes	−2.90	Soluble
15	318.46	3.27	0	1	5	82.63	High	No	−5.47	Moderately
17	295.33	3.43	0	3	6	40.46	High	Yes	−4.74	Moderately
18	247.29	2.14	0	3	6	40.46	High	Yes	−3.01	Soluble
19	261.32	2.49	0	3	7	40.46	High	Yes	−3.35	Soluble
20	275.34	2.80	0	3	7	40.46	High	Yes	−3.62	Soluble
21	289.37	3.03	0	3	7	40.46	High	Yes	−3.87	Soluble
22	275.34	2.81	0	3	7	40.46	High	Yes	−3.61	Soluble
23	303.40	3.50	0	3	9	40.46	High	Yes	−4.36	Moderately
24	309.36	3.32	0	3	7	40.46	High	Yes	−4.73	Moderately
25	323.39	3.41	0	3	8	40.46	High	Yes	−4.82	Moderately
26	323.39	3.47	0	3	7	40.46	High	Yes	−4.83	Moderately
27	337.41	3.75	0	3	8	40.46	High	Yes	−5.17	Moderately
29	325.32	2.54	2	5	5	88.76	High	No	−4.25	Moderately

**Table 4:** The results of molecular docking to the 1DNU, 1N5X, and 4COX protein domains of all the compounds analyzed. Melatonin, febuxostat, and indomethacin were used as reference molecules.

PDB ID	Compound	Average Binding Energy [kcal/mol]	Standard Deviation of Binding Energy [kcal/mol]
1DNU	Melatonin	−5.3	0.15
	**2**	−5.4	0.21
	**5**	−5.5	0.25
	**15**	−5.2	0.15
1N5X	Febuxostat	−7.0	0.32
	**2**	−7.9	0.41
	**5**	−7.8	0.61
	**15**	−7.5	0.45
4COX	Indomethacin	−8.0	0.82
	**2**	−7.4	0.39
	**5**	−7.3	0.61
	**15**	−7.2	0.15

**Table 5:** The search spaces of the analyzed binding sites of the protein domains.

PDB ID	Search Space Center (x, y, z)	Size of the Search Space (x, y, z)
1DNU	39.637, −38.454, −5.011	24, 24, 26
1N5X	96.559, 55.159, 39.980	24, 22, 40
4COX	23.941, 21.867, 13.892	26, 26, 32

## Data Availability

Data are contained within the article and Supplementary Materials.

## Supplementary Materials

The following supporting information can be downloaded at: https://www.mdpi.com/article/10.3390/ijms25105364/s1.
