# Supplementary material for: Novel C3-Methylene-Bridged Indole Derivatives with and without Substituents at N1: The Influence of Substituents on Their Hemolytic, Cytoprotective, and Antimicrobial Activity
Source: Int J Mol Sci. 2024 May 14;25(10):5364. doi: 10.3390/ijms25105364 (PMC11121452; doi:10.3390/ijms25105364)
Supplement: Supplementary file 1 [file ijms-25-05364-s001.zip › ijms-2989670-supplementary.pdf]

Supplementary data

Novel C3-methylene-bridged indole derivatives with and without substituents at N1: the influence of substituents on their hemolytic, cytoprotective and antimicrobial activity

Karolina Babijczuk[1], Natalia Berdzik[1], Damian Nowak[2], Beata Warżajtis[3], Urszula Rychlewska[3], Justyna Starzyk[4], Lucyna Mrówczyńska[5], and Beata Jasiewicz[1,*]

[1] Department of Bioactive Products, Faculty of Chemistry, Adam Mickiewicz University, Uniwersytetu Poznańskiego 8, 61-614 Poznań, Poland

[2] Department of Quantum Chemistry, Faculty of Chemistry, Adam Mickiewicz University, Uniwersytetu Poznańskiego 8, 61-614 Poznań, Poland

[3] Department of Crystallography, Faculty of Chemistry, Adam Mickiewicz University, Uniwersytetu Poznańskiego 8, 61-614 Poznań, Poland

[4] Department of Soil Science and Microbiology, Faculty of Agronomy, Horticulture, and Bioengineering, University of Life Science, Szydłowska 50, 60-656 Poznań, Poland

[5] Department of Cell Biology, Faculty of Biology, Adam Mickiewicz University, Uniwersytetu Poznańskiego 6, 61-614 Poznań, Poland

***** Correspondence: beatakoz@amu.edu.pl (B.J.)

TABLE OF CONTENTS

^{1}H and ^{13}C NMR spectra of compound **2**…………..….…………………………….….S3
EI-MS and FIR spectra of compound **2**…..…………………………………………..S4
^{1}H and ^{13}C NMR spectra of compound **3**………………………………………….S5
EI-MS and IR spectra of compound **3**……..……………………………………….S6
^{1}H and ^{13}C NMR spectra of compound **4**………………………………………….S7
EI-MS and IR spectra of compound **4**……..………………………………………S8
^{1}H and ^{13}C NMR spectra of compound **5**…………………………………….....S9
EI-MS and IR spectra of compound **5**……..…………………………….…..….S10
^{1}H and ^{13}C NMR spectra of compound **6**…………………………………..…….S11
EI-MS and IR spectra of compound **6**……..……………………………….….…S12
^{1}H and ^{13}C NMR spectra of compound **7**…………………………………..….S13
EI-MS and IR spectra of compound **7**……..…………………..……………….S14
^{1}H and ^{13}C NMR spectra of compound **8**…………………………………….....S15
EI-MS and IR spectra of compound **8**……..……………………………….….S16
^{1}H and ^{13}C NMR spectra of compound **9**……………………………….….….S17

EI-MS and IR spectra of compound **9**……………………………………………………..….S18
^{1}H and ^{13}C NMR spectra of compound **10**………………………………………………S19
EI-MS and IR spectra of compound **10**…..………………………………………………….S20
^{1}H and ^{13}C NMR spectra of compound **11**………..………………………………………..S21
EI-MS and IR spectra of compound **11**…..………………………………………………….S22
^{1}H and ^{13}C NMR spectra of compound **12**………………………………………………..S23
EI-MS and IR spectra of compound **12**…..…………………………………………………..S24
^{1}H and ^{13}C NMR spectra of compound **13**………………………………………………..S25
EI-MS and IR spectra of compound **13**…..…………………………………………………..S26
^{1}H and ^{13}C NMR spectra of compound **15**………………………………………………..S27
EI-MS and IR spectra of compound **15**…..……………………………………………….….S28
^{1}H and ^{13}C NMR spectra of compound 1**7**……………………………………..……..S29
EI-MS and IR spectra of compound 1**7**…..……………………………………………...….S30
^{1}H and ^{13}C NMR spectra of compound 1**8**…………………………………………...….S31
EI-MS and IR spectra of compound 1**8**…..……………………………..………………….S32
^{1}H and ^{13}C NMR spectra of compound 1**9**…………………………………………………...S33
EI-MS and IR spectra of compound 1**9**…..……………………………………………...….S34
^{1}H and ^{13}C NMR spectra of compound **20**…………………………………………………...S35
EI-MS and IR spectra of compound **20**…..…………………………………………………...S36
^{1}H and ^{13}C NMR spectra of compound **21**……………………………………………………S37
EI-MS and IR spectra of compound **21**…..…………………………………………………….S38
^{1}H and ^{13}C NMR spectra of compound **22**………..…………………………………………S39
EI-MS and IR spectra of compound **22**…..…………………………………………………….S40
^{1}H and ^{13}C NMR spectra of compound **23**……………………………………………………S41
EI-MS and IR spectra of compound **23**…..…………………………………………………….S42
^{1}H and ^{13}C NMR spectra of compound **24**……………………………………………………S43
EI-MS and IR spectra of compound **24**…..…………………………………………………….S44
^{1}H and ^{13}C NMR spectra of compound **25**……………………………………………….....S45
EI-MS and IR spectra of compound **25**…..………………………………………………….…...S46
^{1}H and ^{13}C NMR spectra of compound **26**……………………………………..……..S47
EI-MS and IR spectra of compound **26**…..………………………………………..……..S48
^{1}H and ^{13}C NMR spectra of compound **27**……………………………………..…..S49
EI-MS and IR spectra of compound **27**…..…………………………..………………..S50
^{1}H and ^{13}C NMR spectra of compound **29**……………………………………………....S51
EI-MS and IR spectra of compound **29**…..……………………………………..…..S52
Crystal data Description of the crystallographic results……………………………………S53
Table S1. Hydrogen bond geometrical parameters………………………………………….S56
Table S2. Crystal data and structure refinement parameters for gramine derivatives……...S58
Table S3. Antibacterial activities of compounds **1-29**…………………………………...…S59
Interactions between indole-based derivatives **2**, **5**, and **15**, and the 1N5X and the
4COX protein domains……………………………………………………………….S60

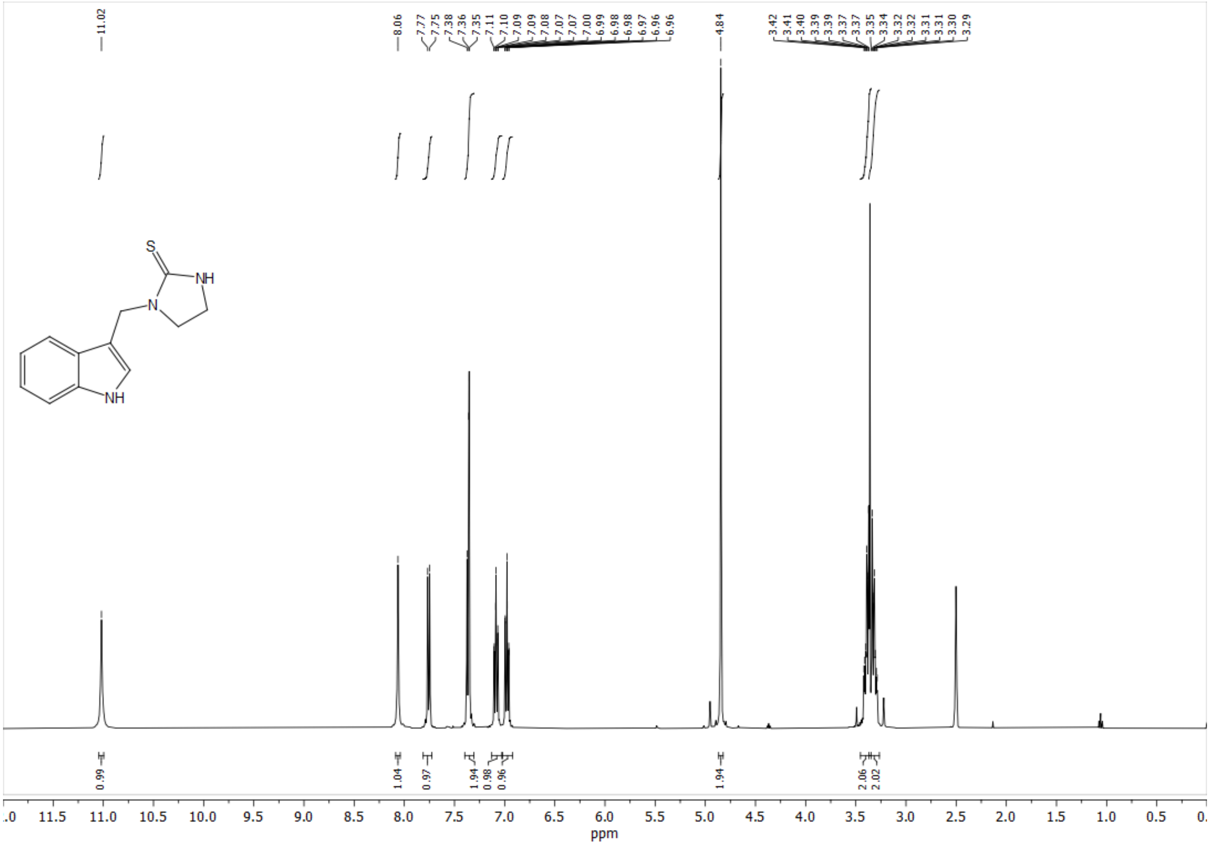

Fig.S1a. ^{1}H NMR spectrum of compound **2**

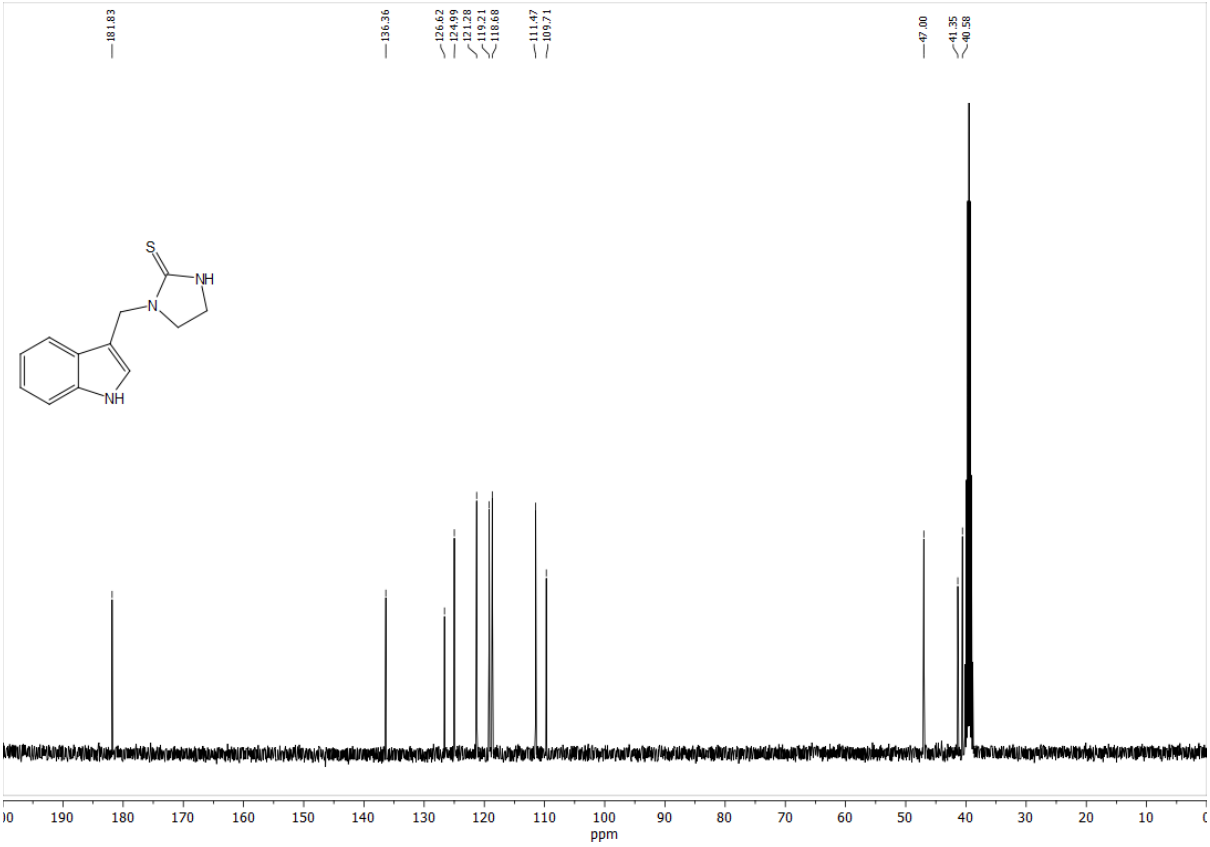

Fig.S1b. ^{13}C NMR spectrum of compound **2**

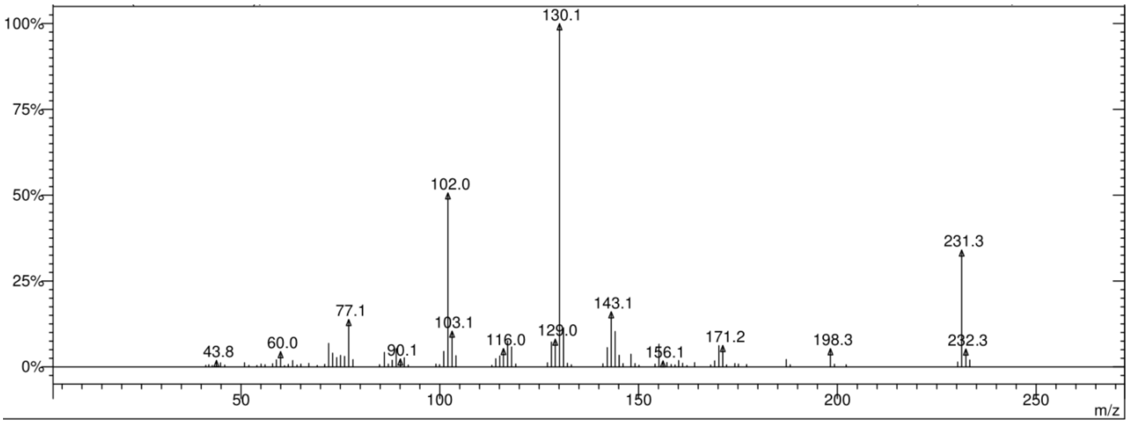

Fig.S1c. EI-MS spectrum of compound **2**

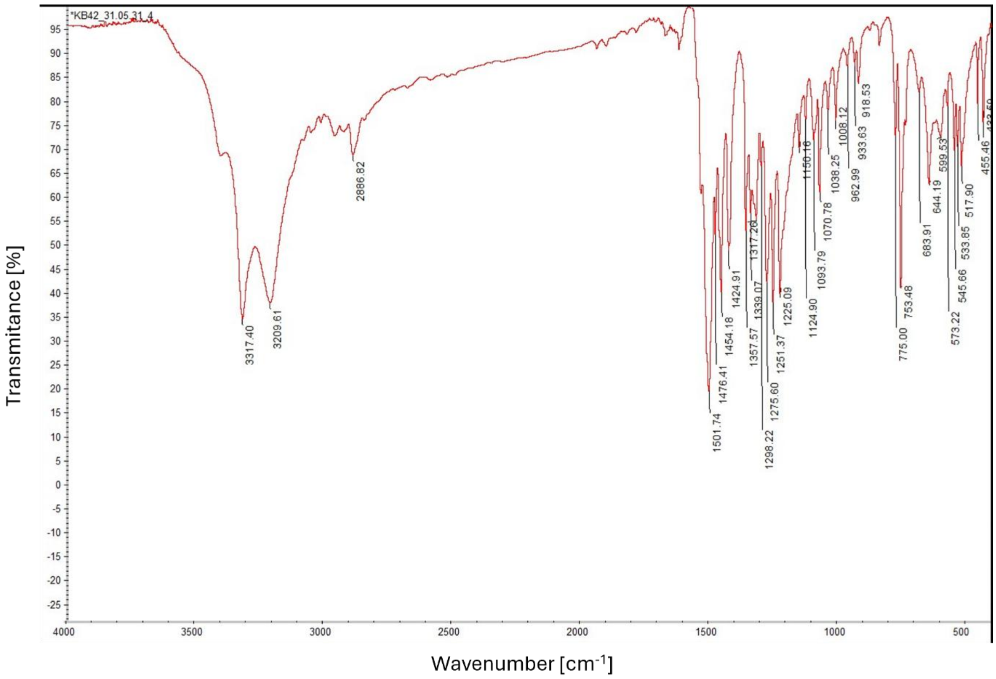

Fig.S1d. IR spectrum of compound **2**

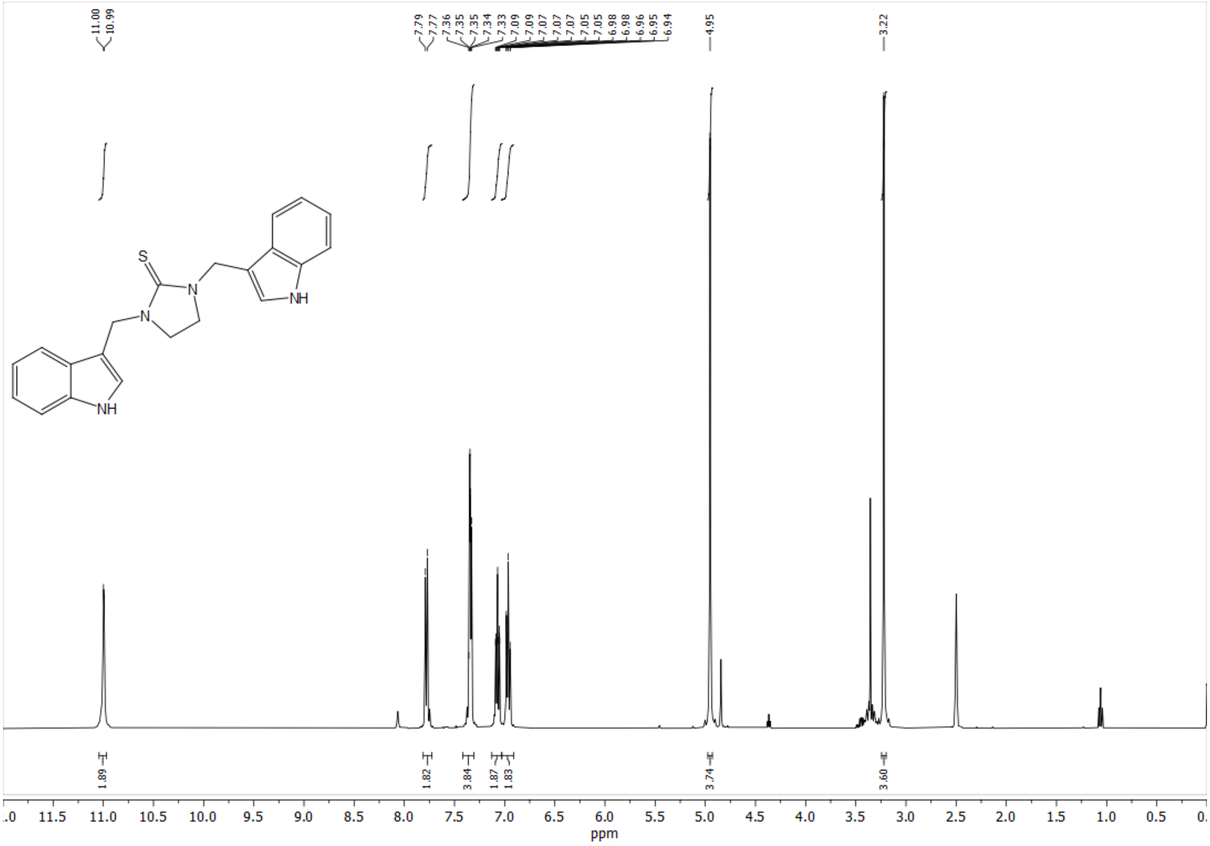

Fig.S2a. ^{1}H NMR spectrum of compound **3**

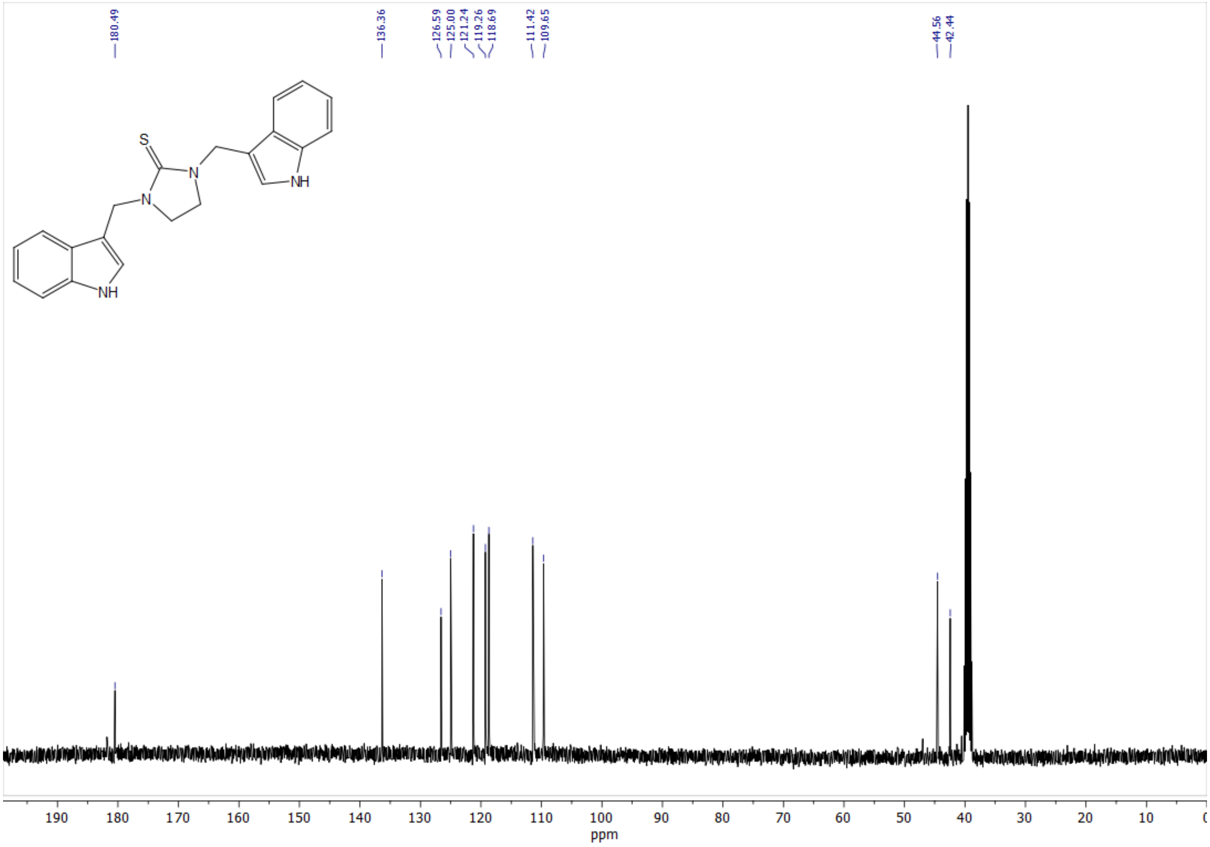

Fig.S2b. ^{13}C NMR spectrum of compound **3**

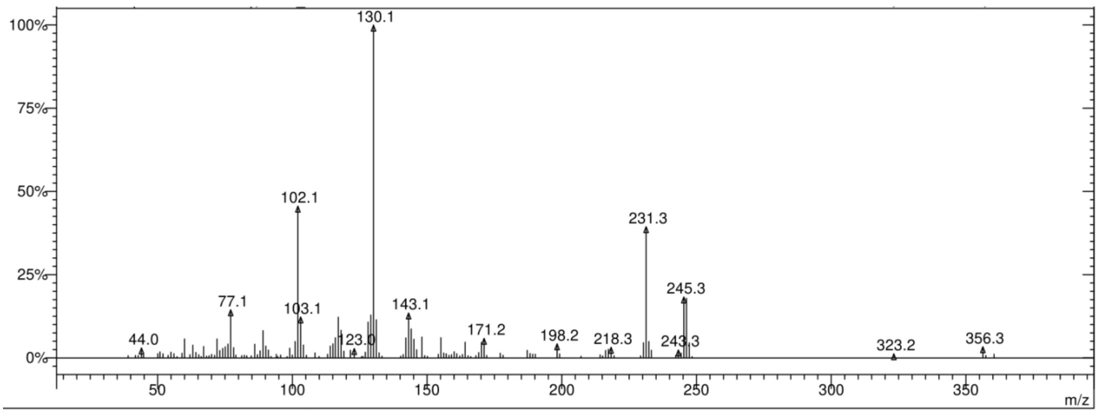

Fig.S2c. EI-MS spectrum of compound **3**

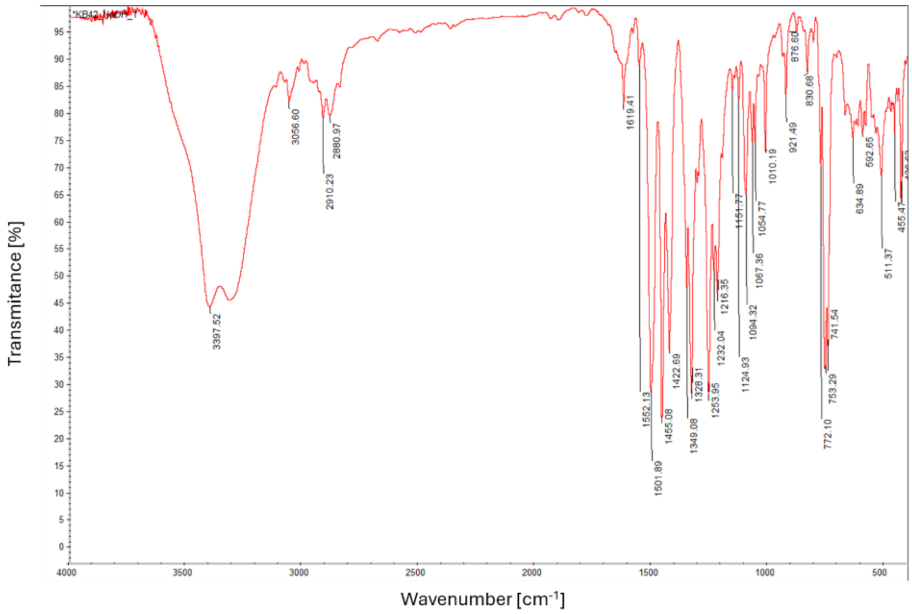

Fig.S2d. IR spectrum of compound **3**

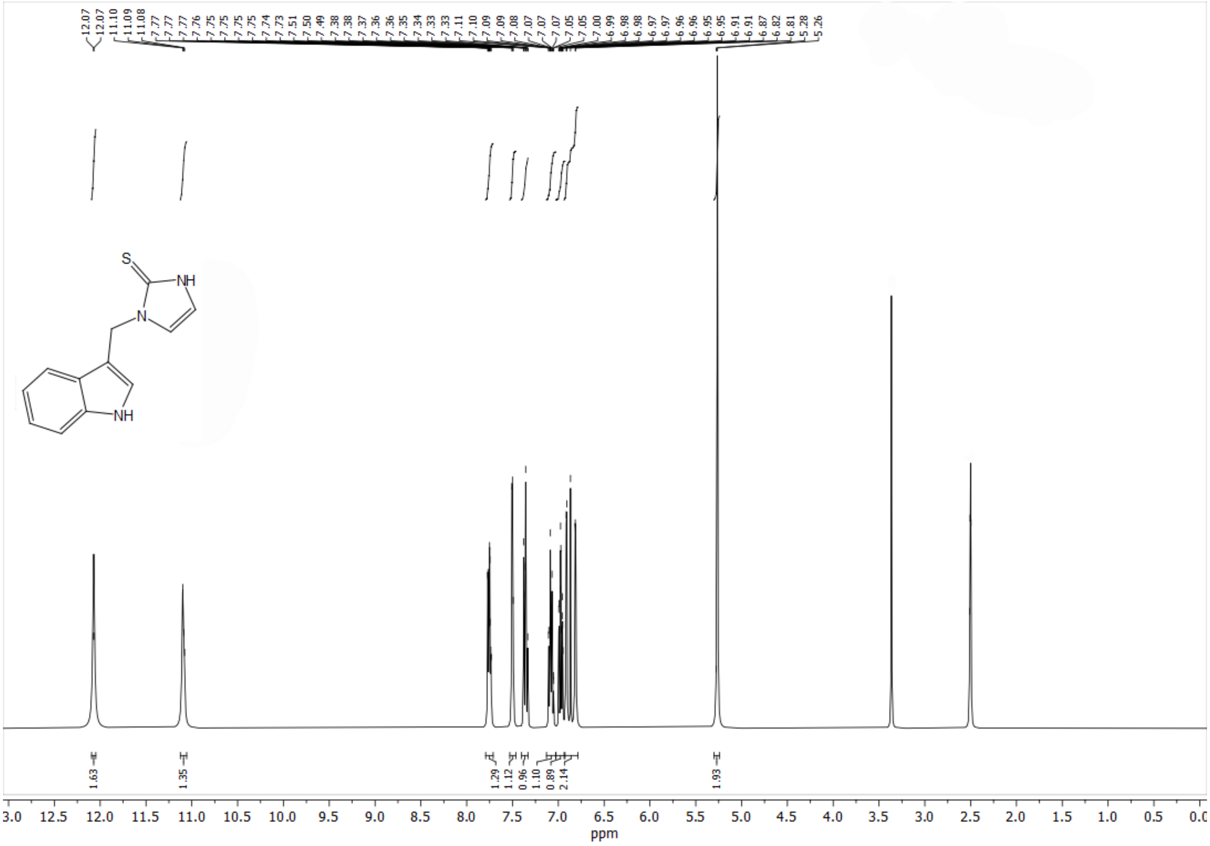

Fig.S3a. ^{1}H NMR spectrum of compound **4**

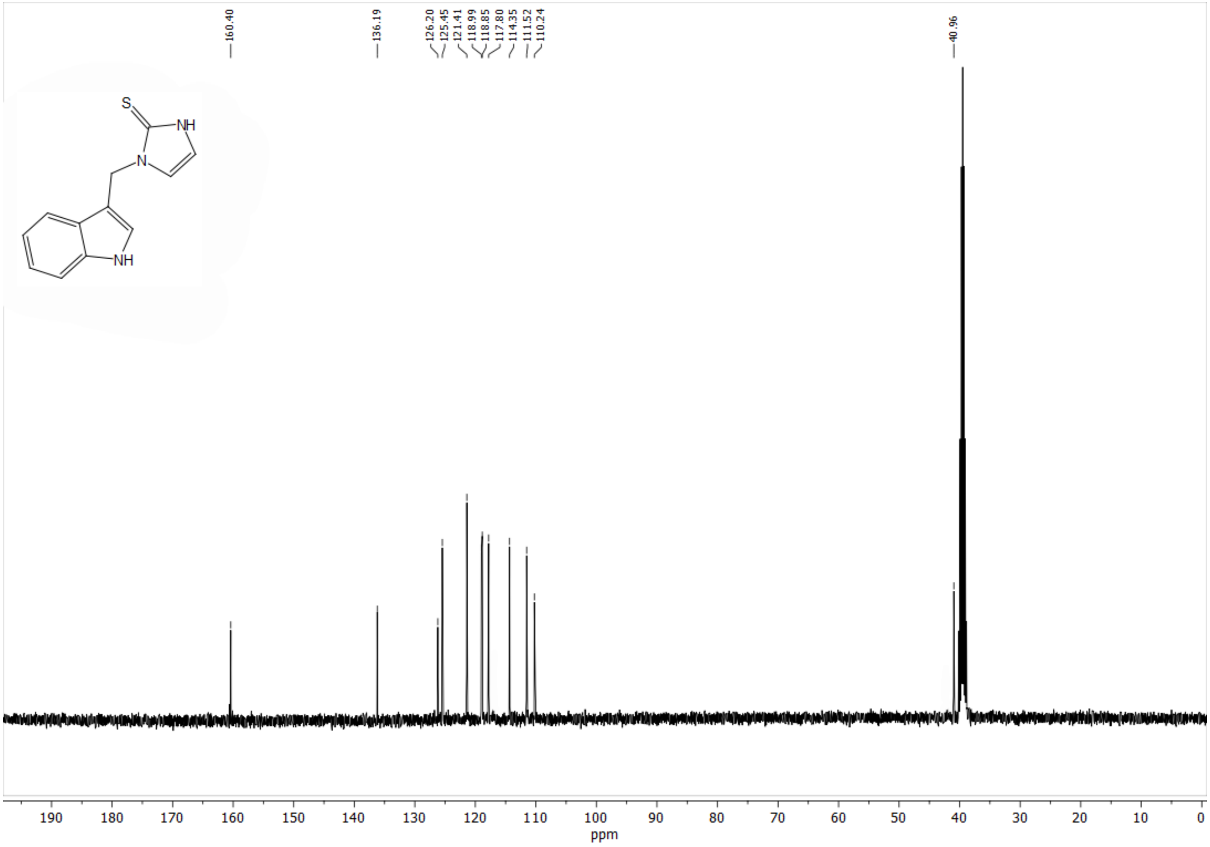

Fig.S3b. ^{13}C NMR spectrum of compound **4**

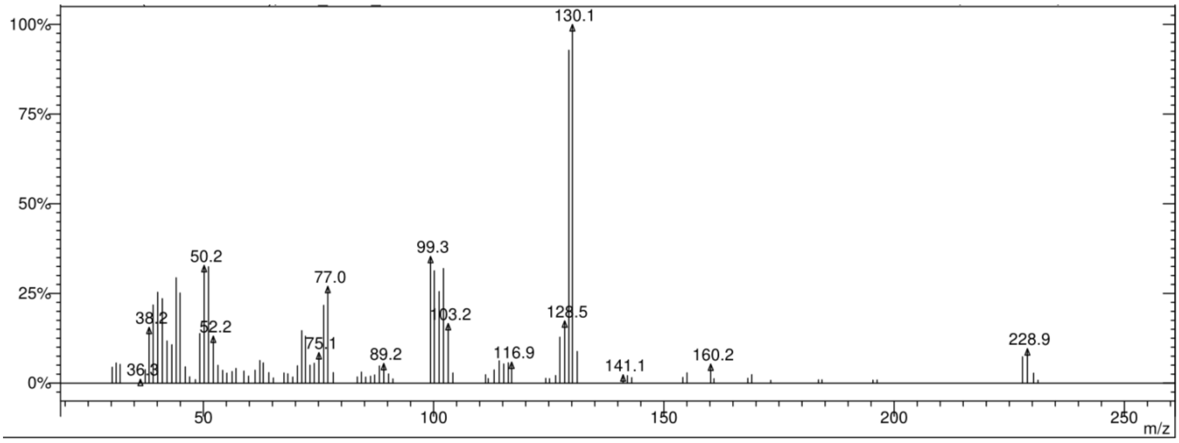

Fig.S3c. EI-MS spectrum of compound **4**

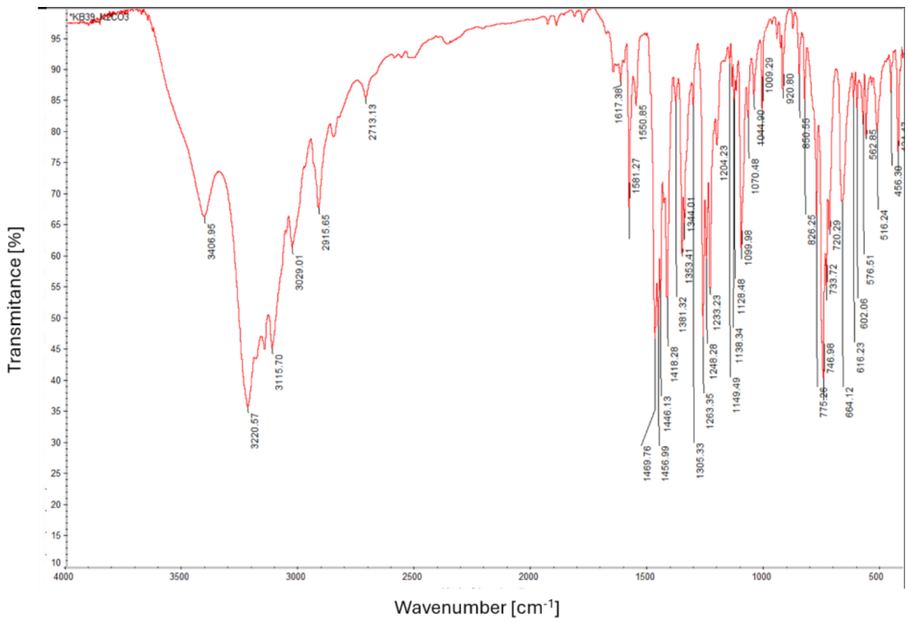

Fig.S3d. IR spectrum of compound **4**

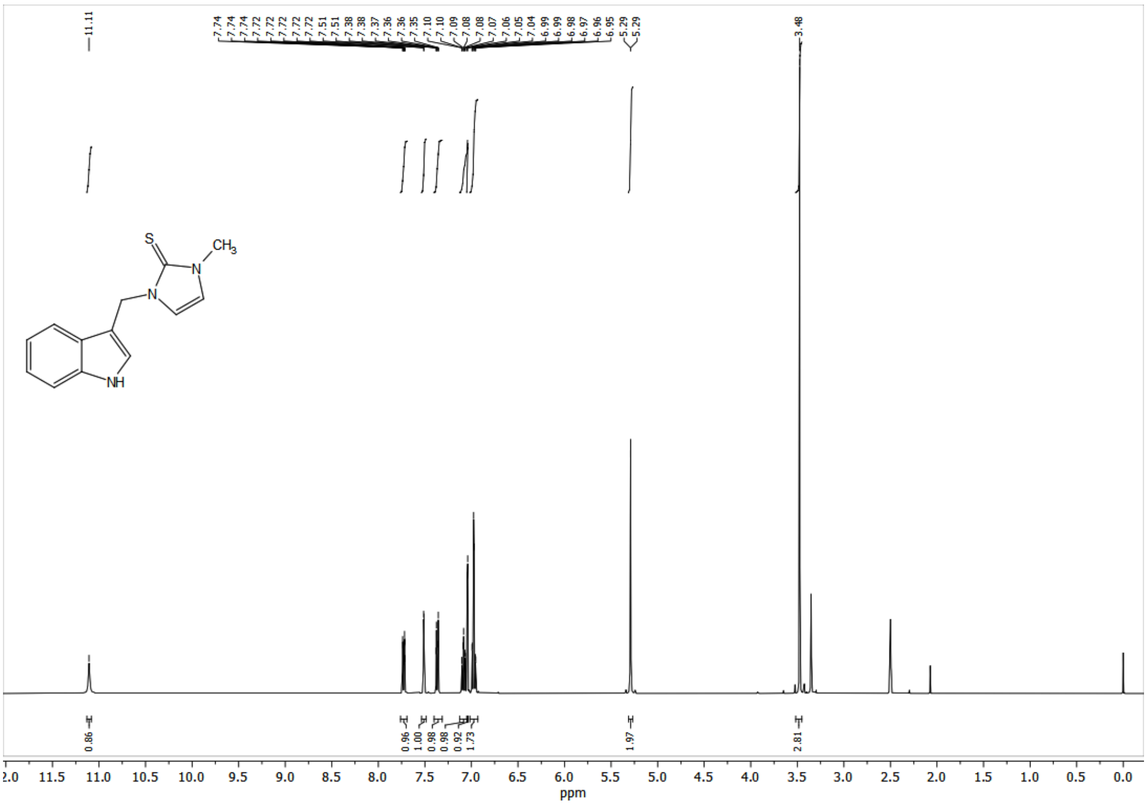

Fig.S4a. ^{1}H NMR spectrum of compound **5**

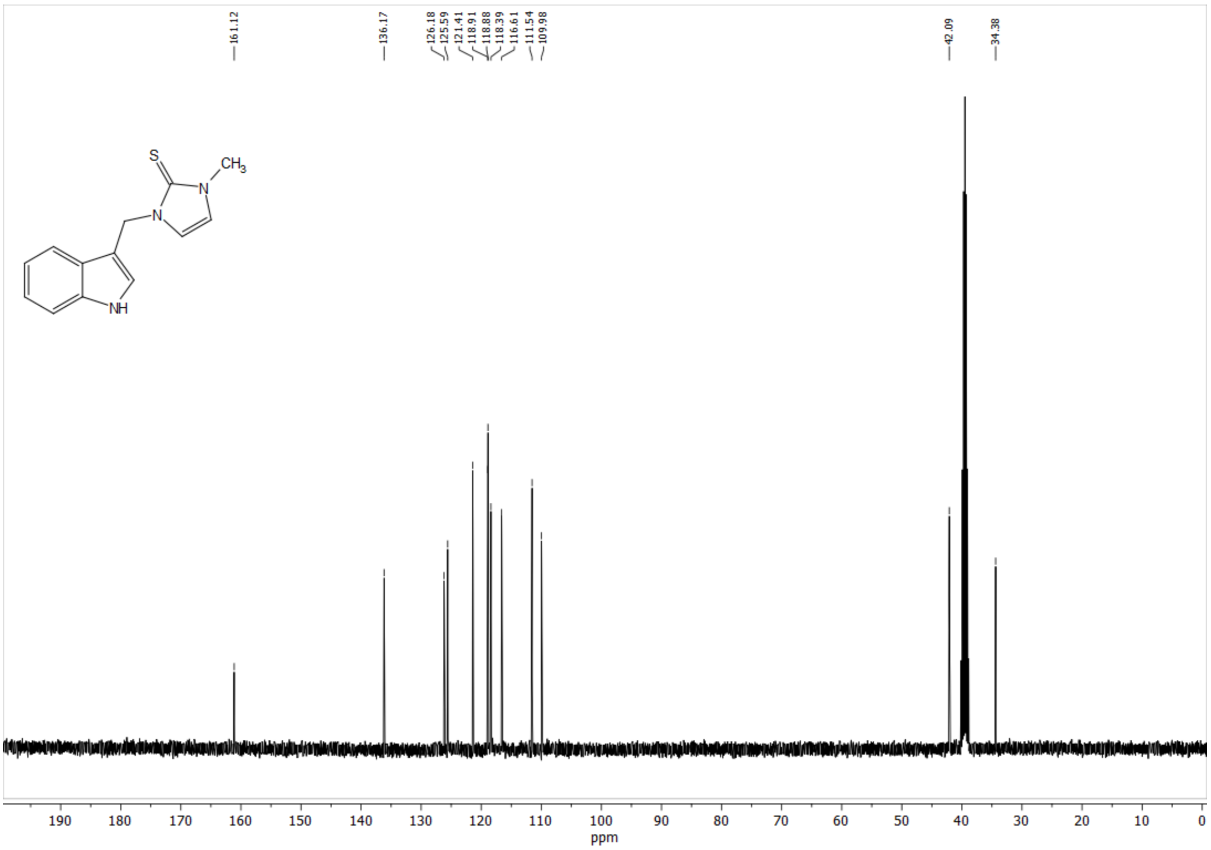

Fig.S4b. ^{13}C NMR spectrum of compound **5**

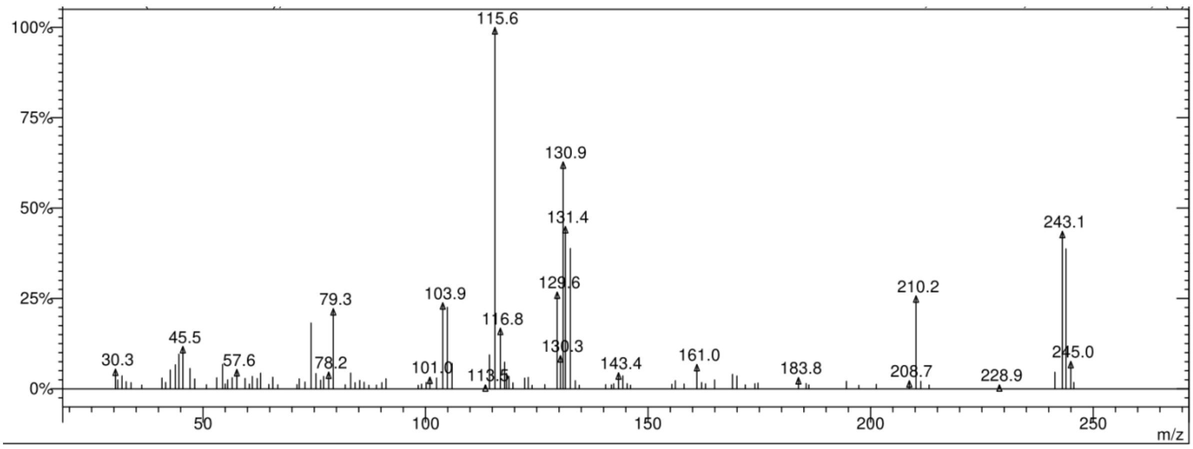

Fig.S4c. EI-MS spectrum of compound **5**

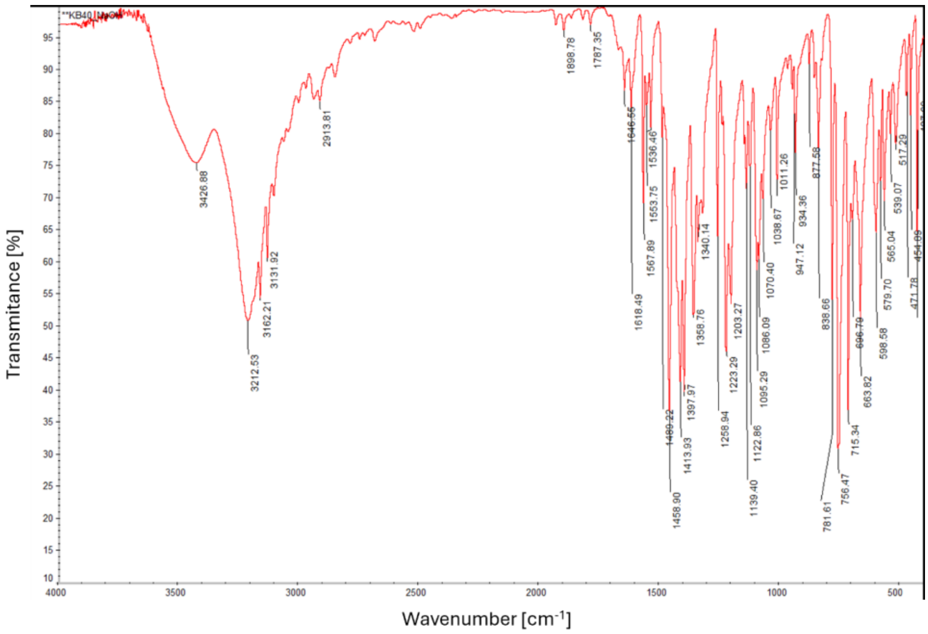

Fig.S4d. IR spectrum of compound **5**

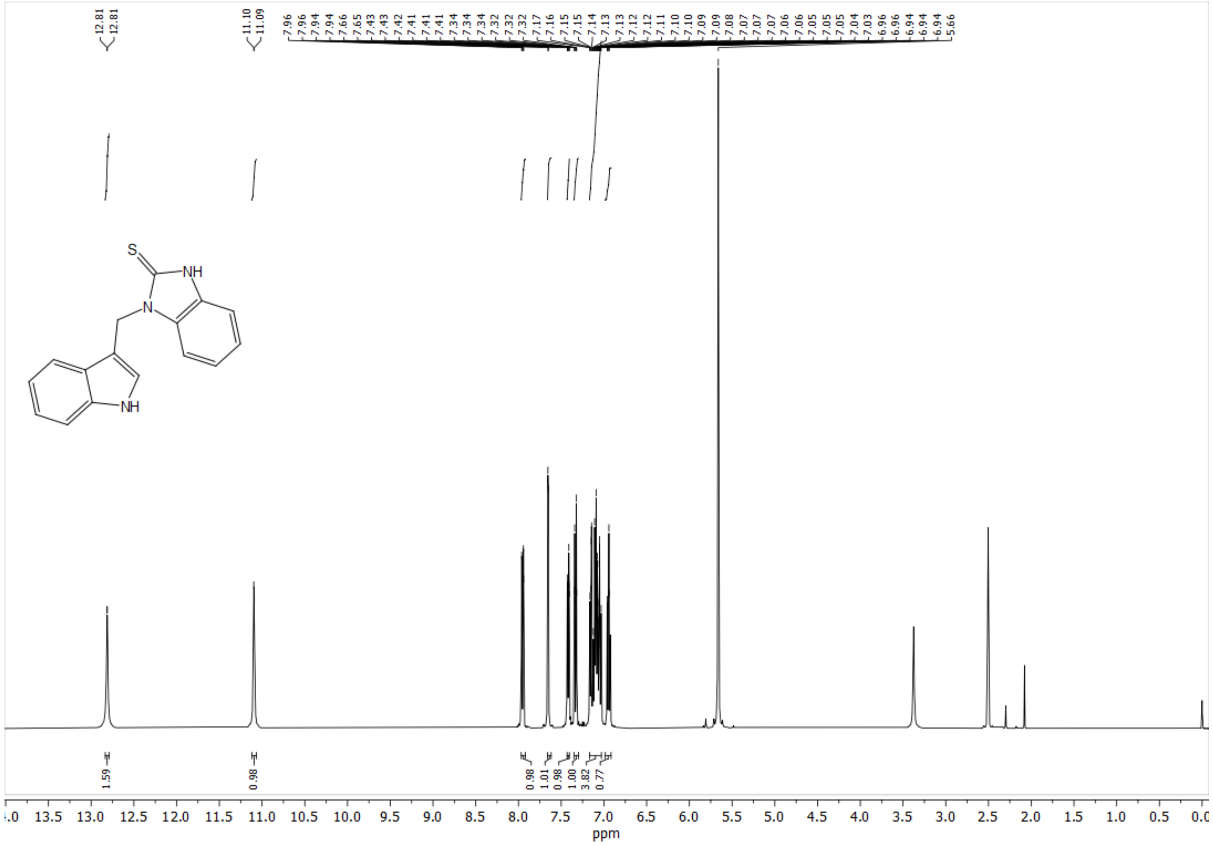

Fig.S5a. ^{1}H NMR spectrum of compound **6**

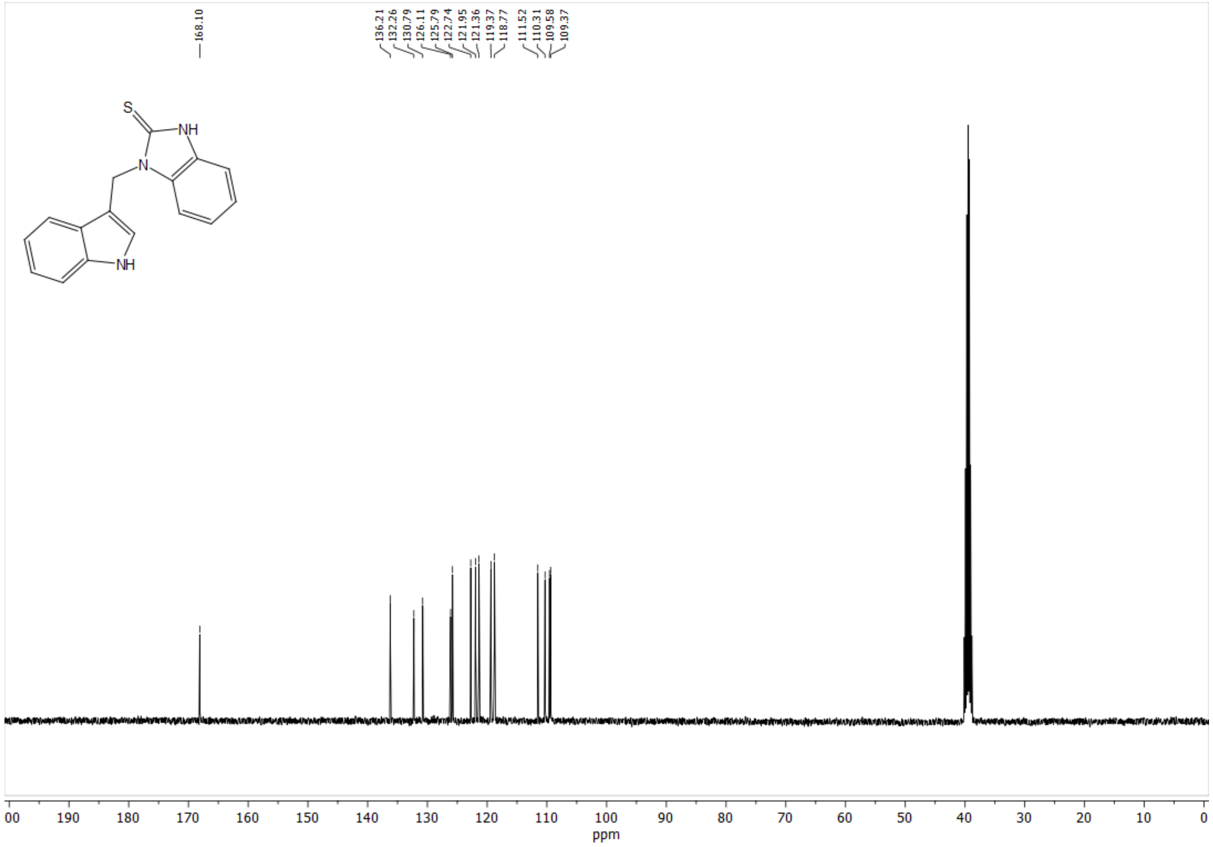

Fig.S5b. ^{13}C NMR spectrum of compound **6**

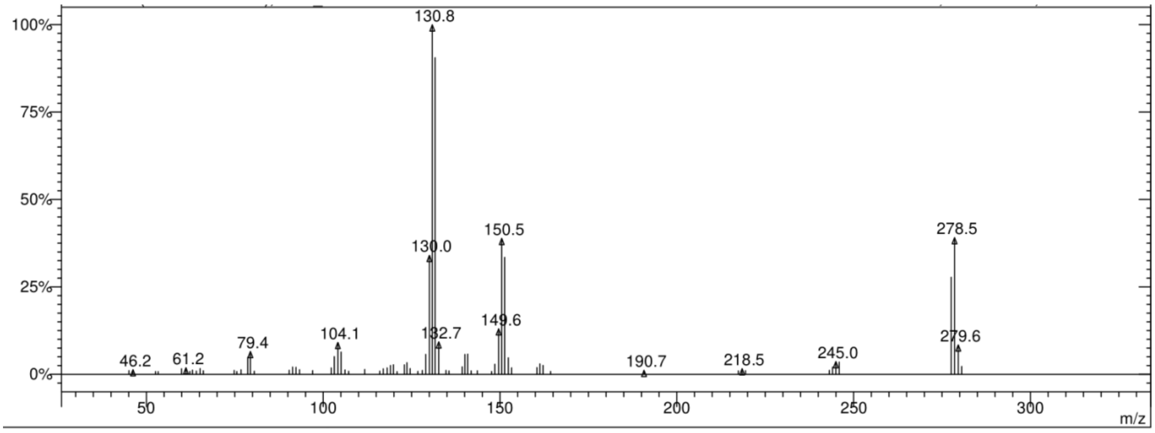

Fig.S5c. EI-MS spectrum of compound **6**

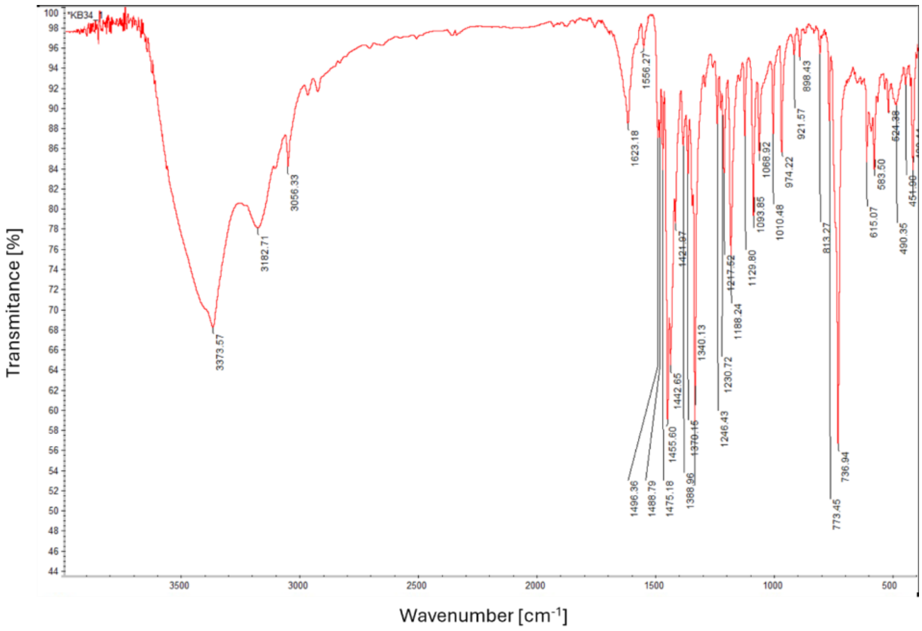

Fig.S5d. IR spectrum of compound **6**

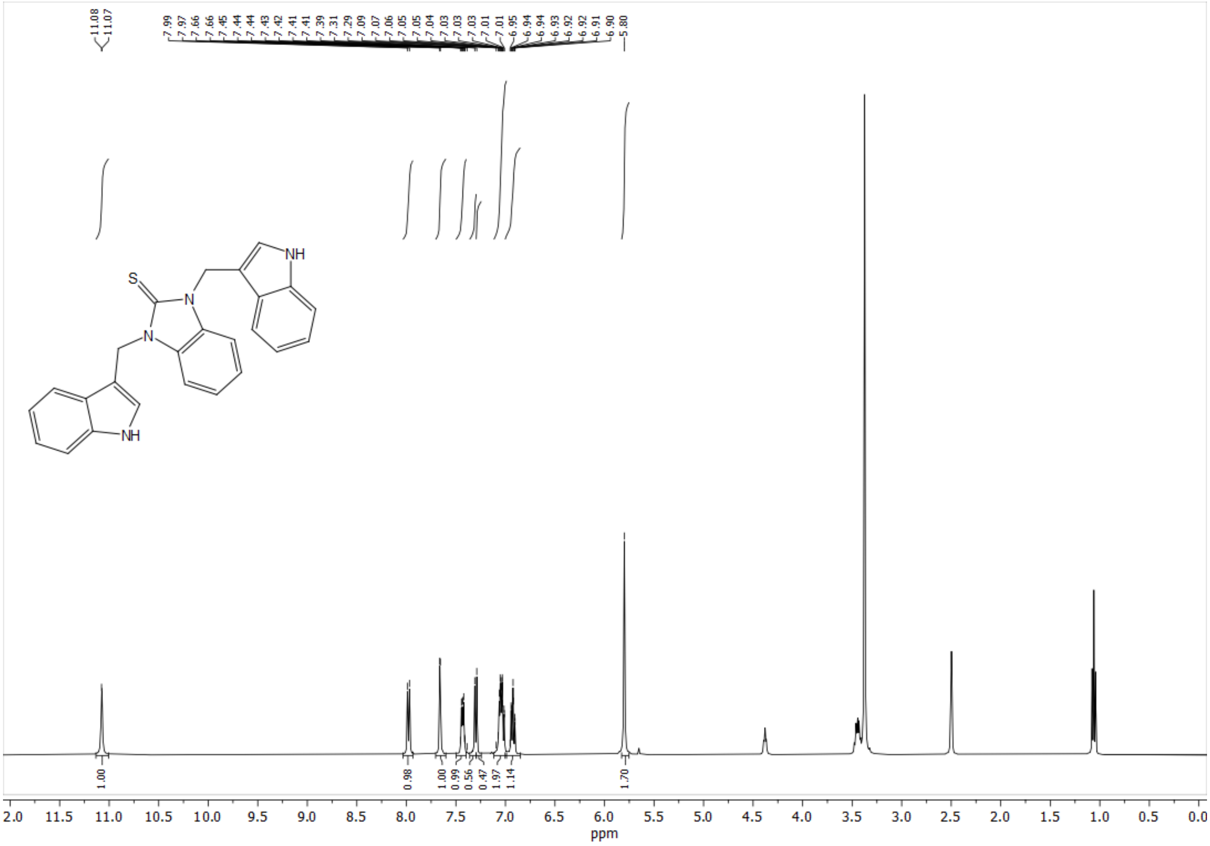

Fig.S6a. ^{1}H NMR spectrum of compound **7**

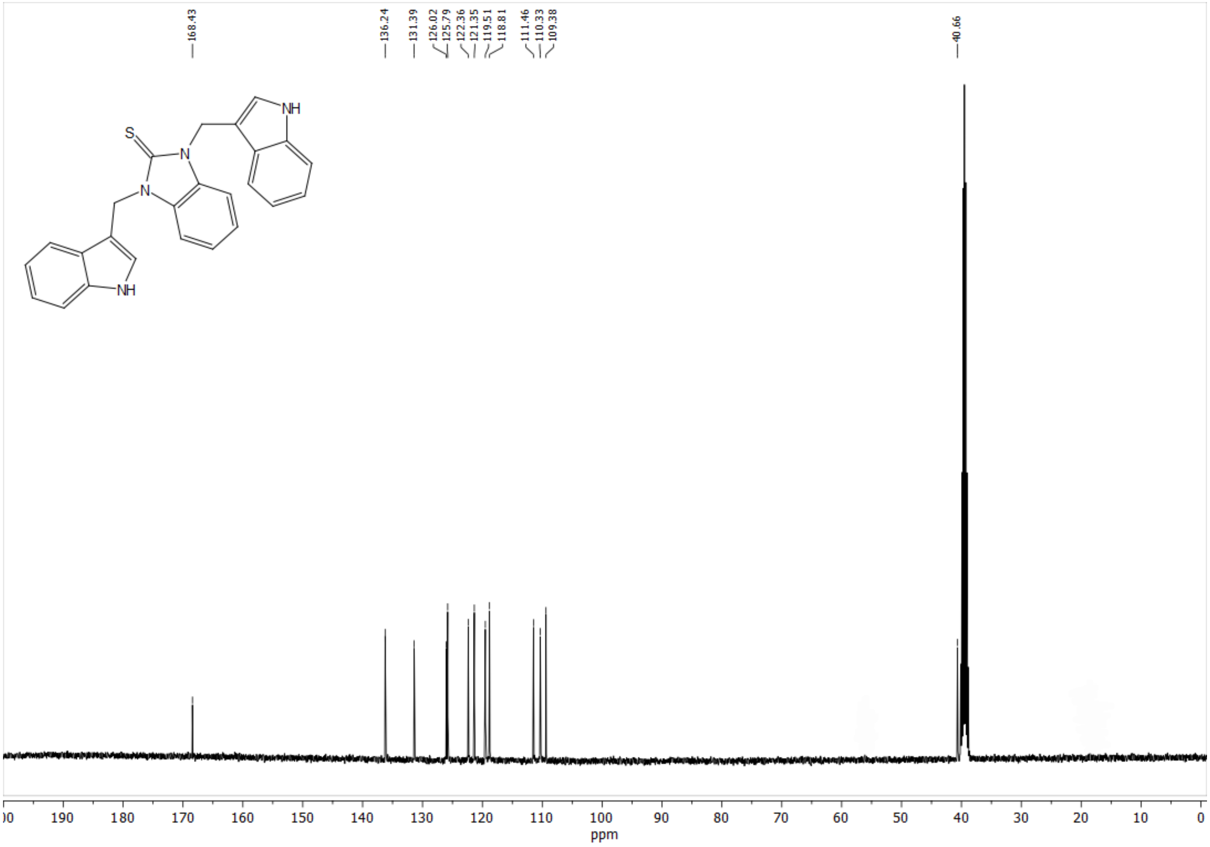

Fig.S6b. ^{13}C NMR spectrum of compound **7**

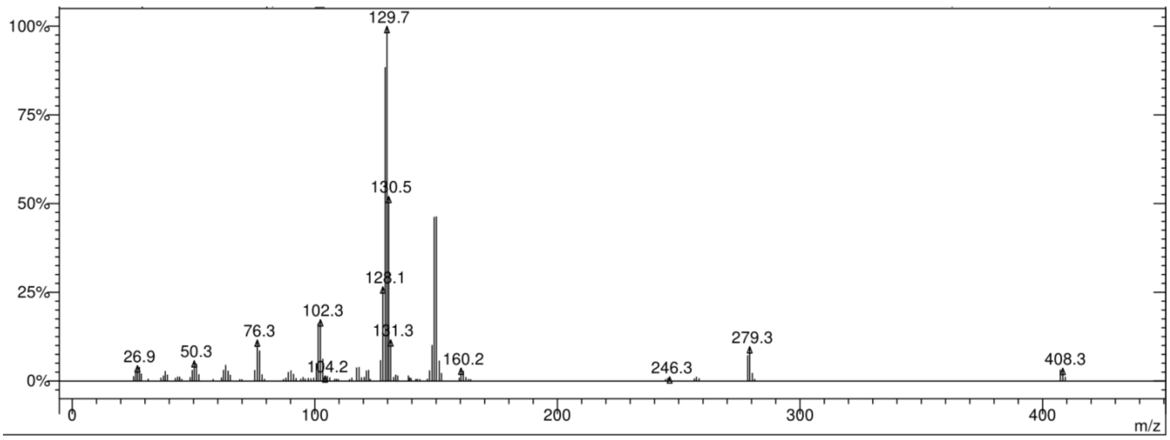

Fig.S6c. EI-MS spectrum of compound **7**

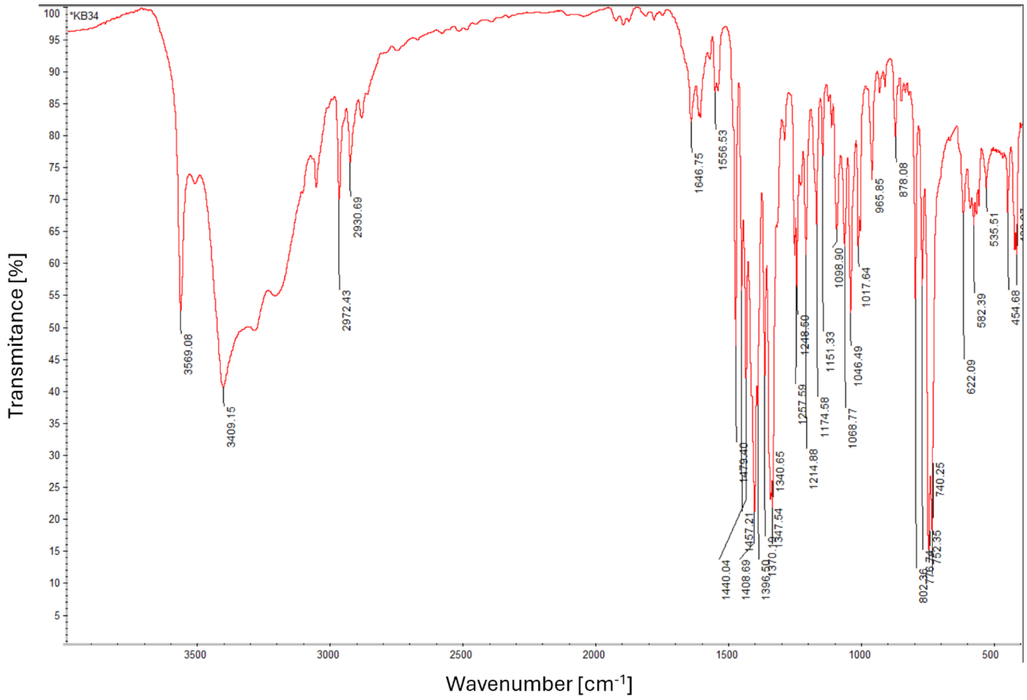

Fig.S6d. IR spectrum of compound **7**

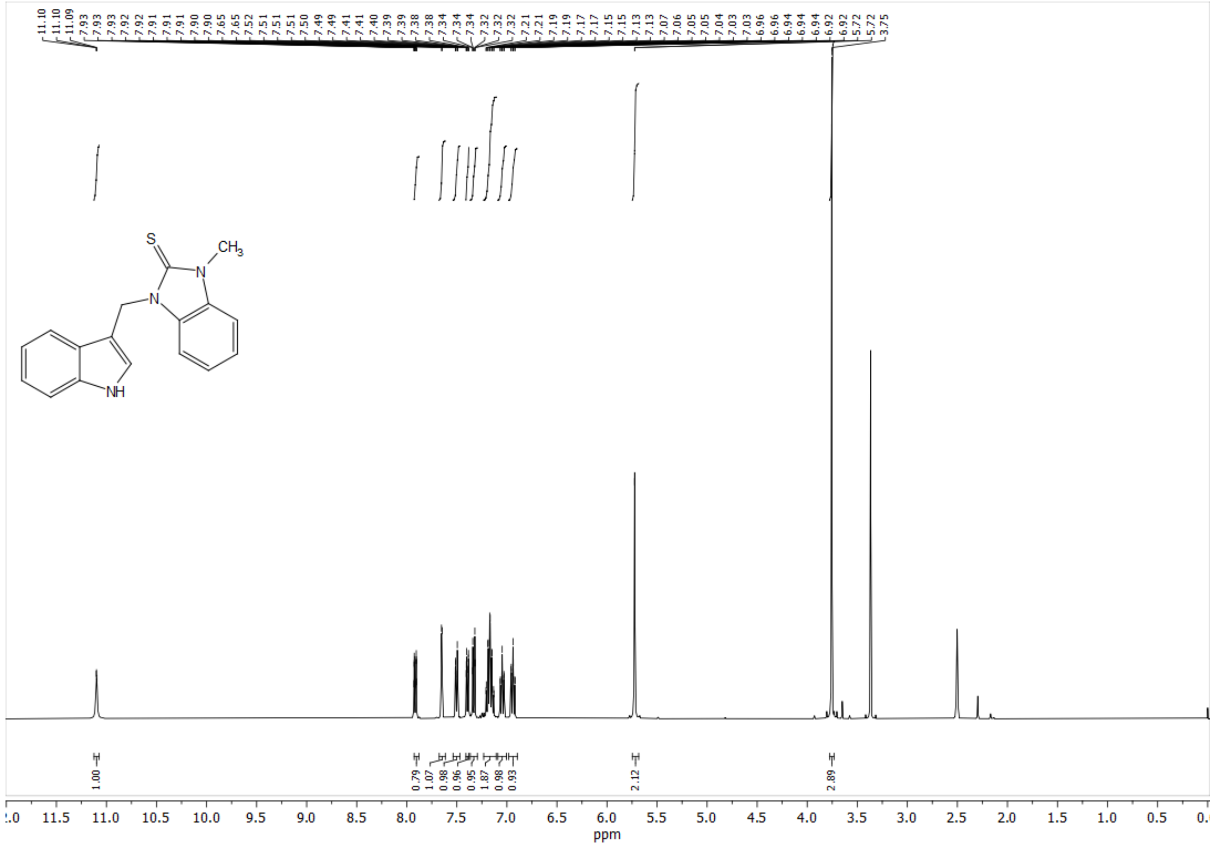

Fig.S7a. ^{1}H NMR spectrum of compound **8**

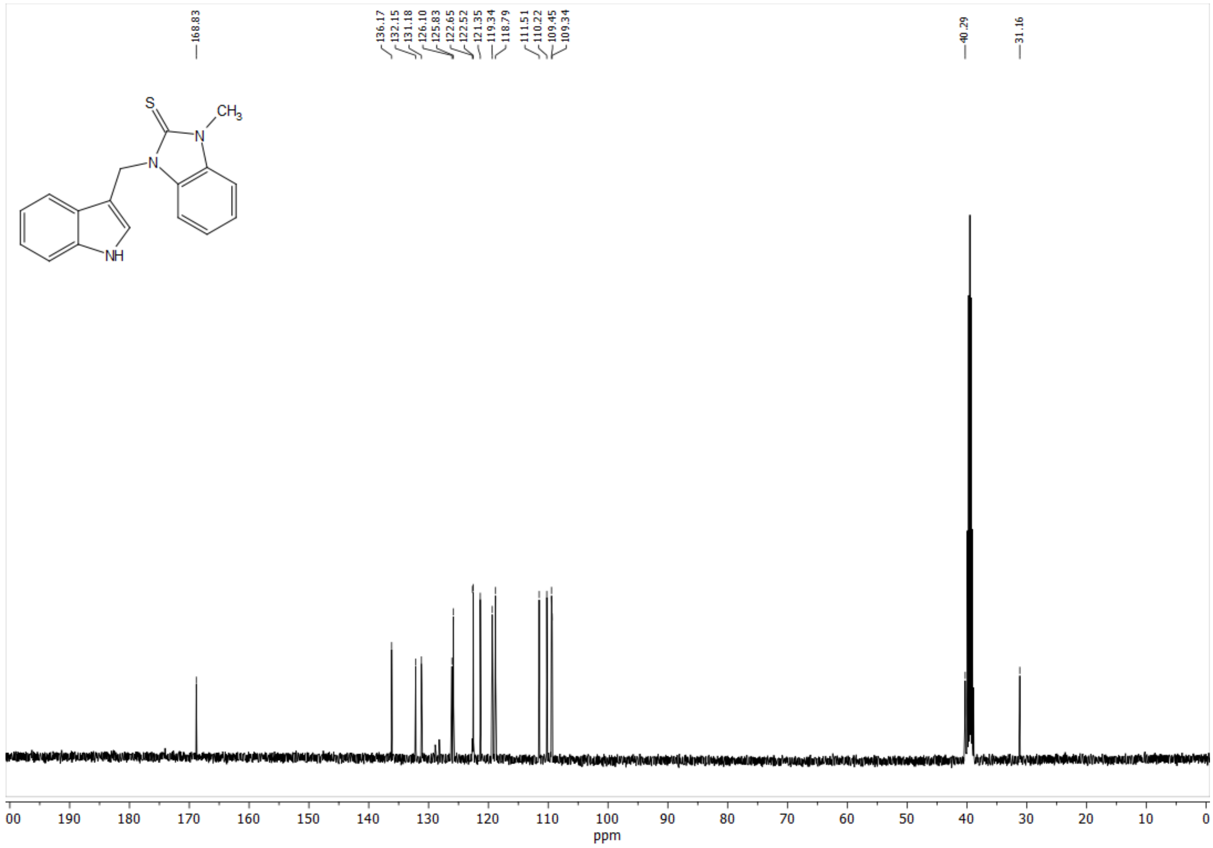

Fig.S7b. ^{13}C NMR spectrum of compound **8**

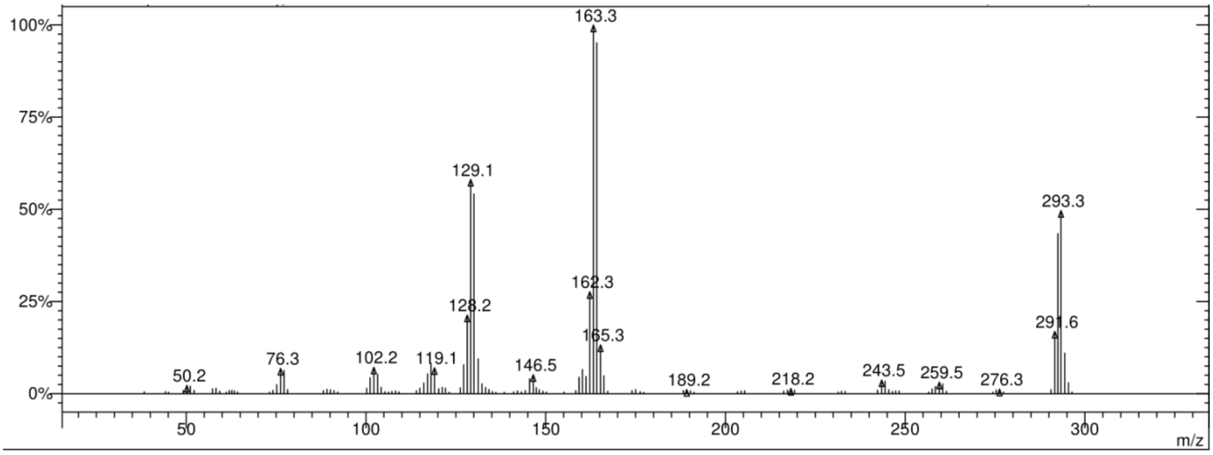

Fig.S7c. EI-MS spectrum of compound **8**

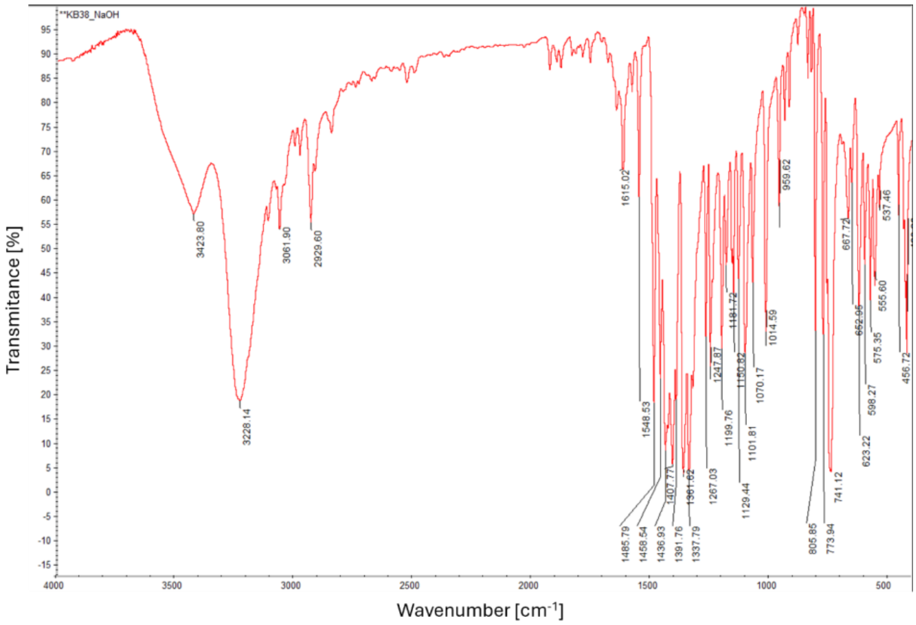

Fig.S7d. IR spectrum of compound **8**

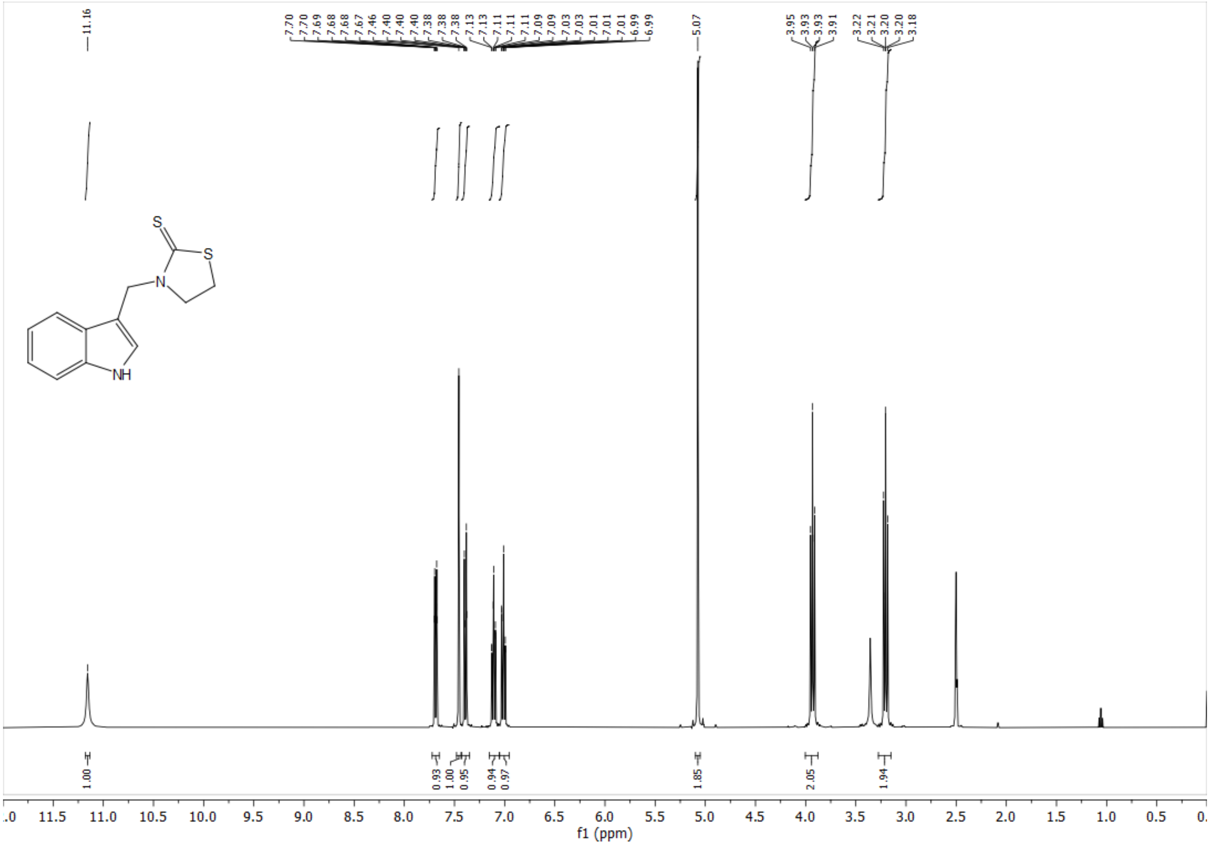

Fig.S8a. ^{1}H NMR spectrum of compound **9**

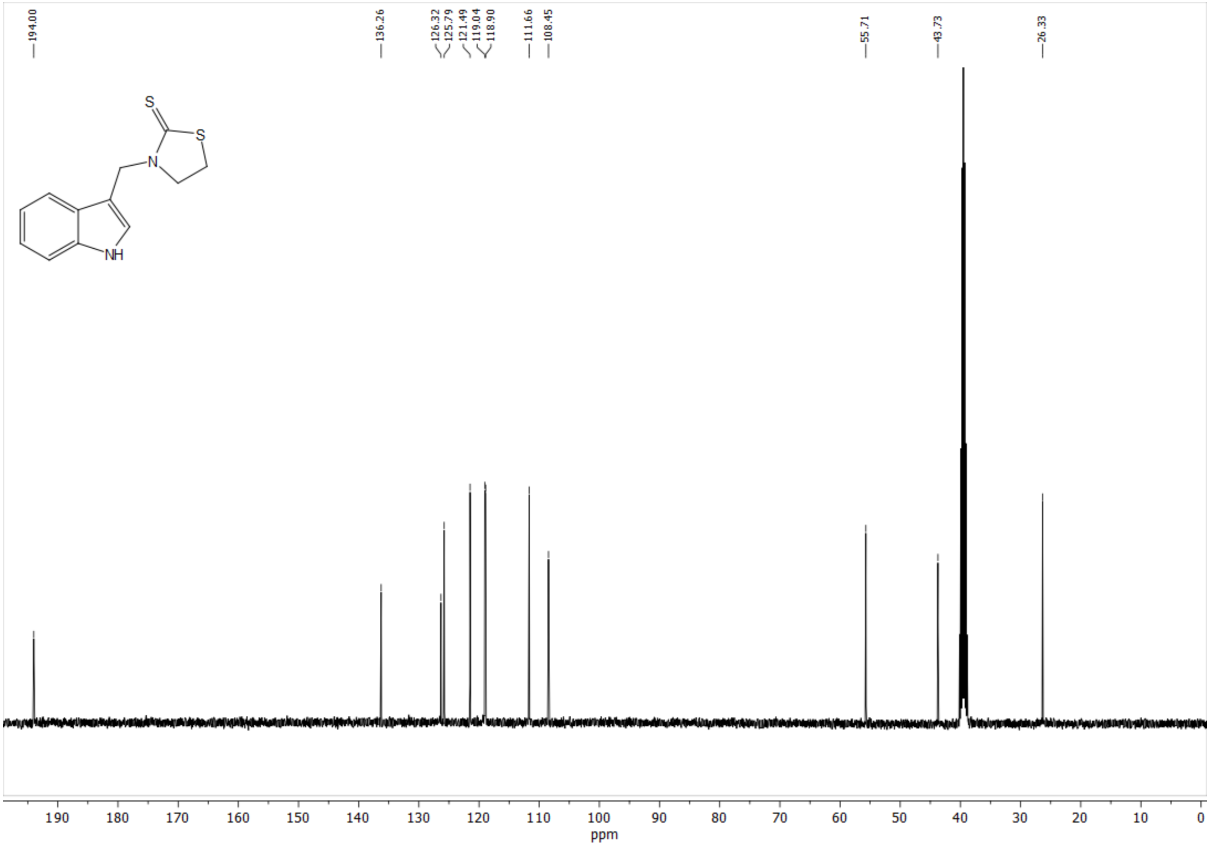

Fig.S8b. ^{13}C NMR spectrum of compound **9**

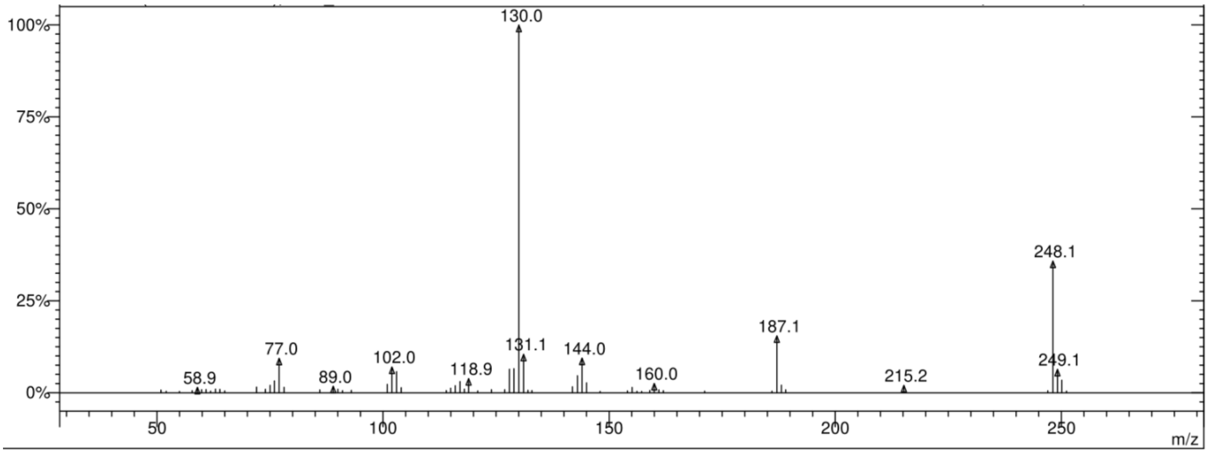

Fig.S8c. EI-MS spectrum of compound **9**

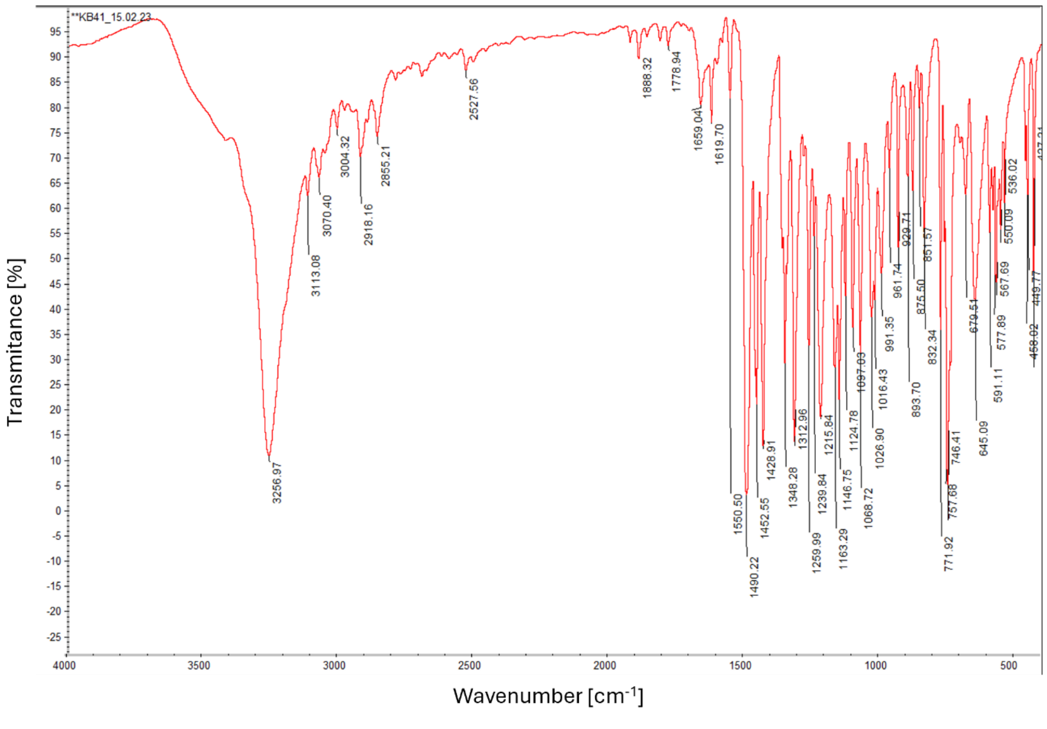

Fig.S8d. IR spectrum of compound **9**

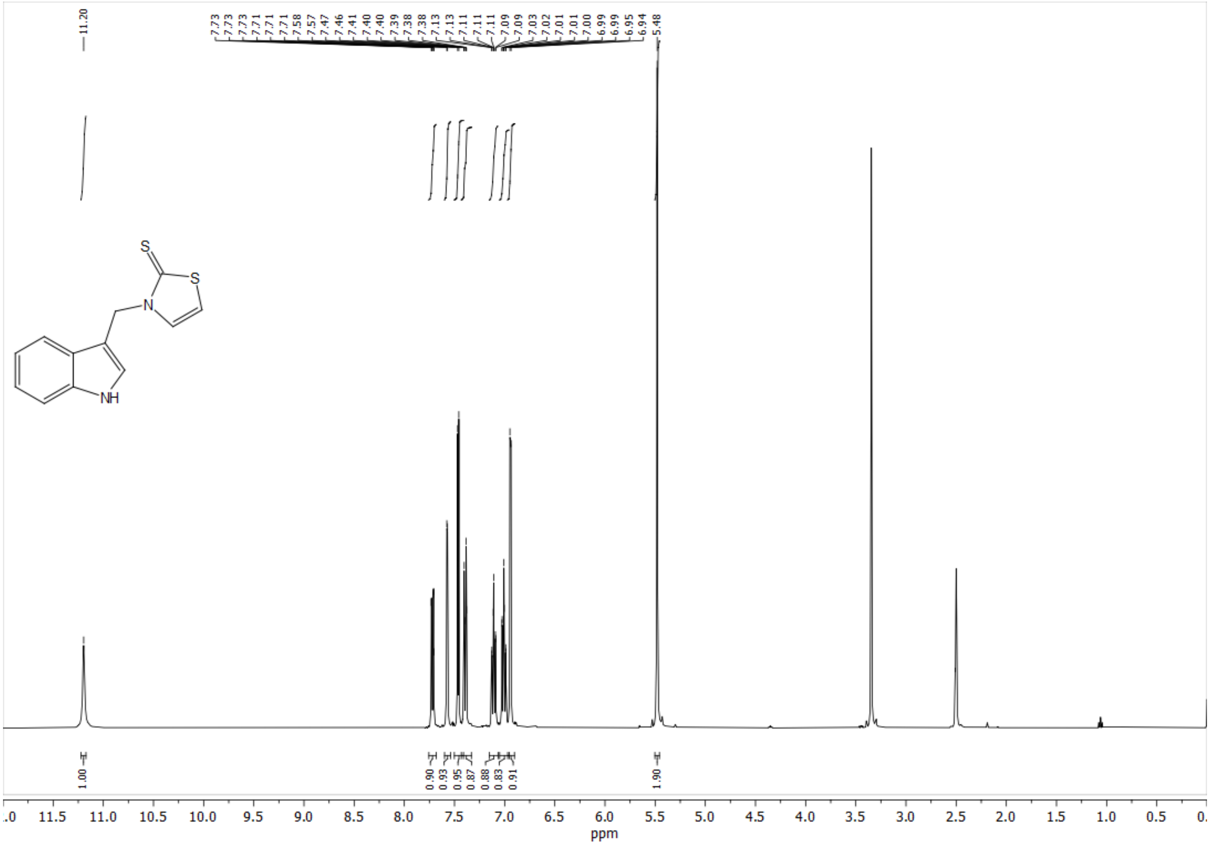

Fig.S9a. ^{1}H NMR spectrum of compound **10**

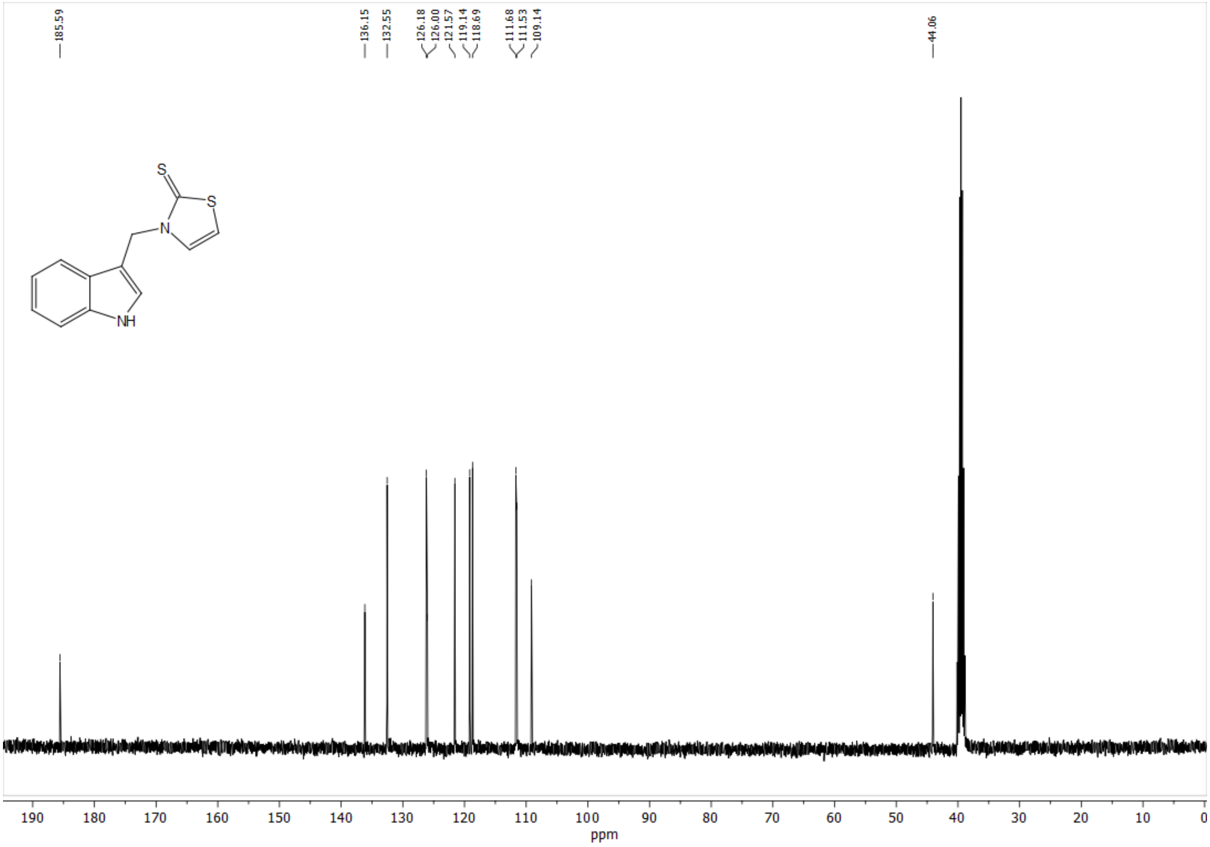

Fig.S9b. ^{13}C NMR spectrum of compound **10**

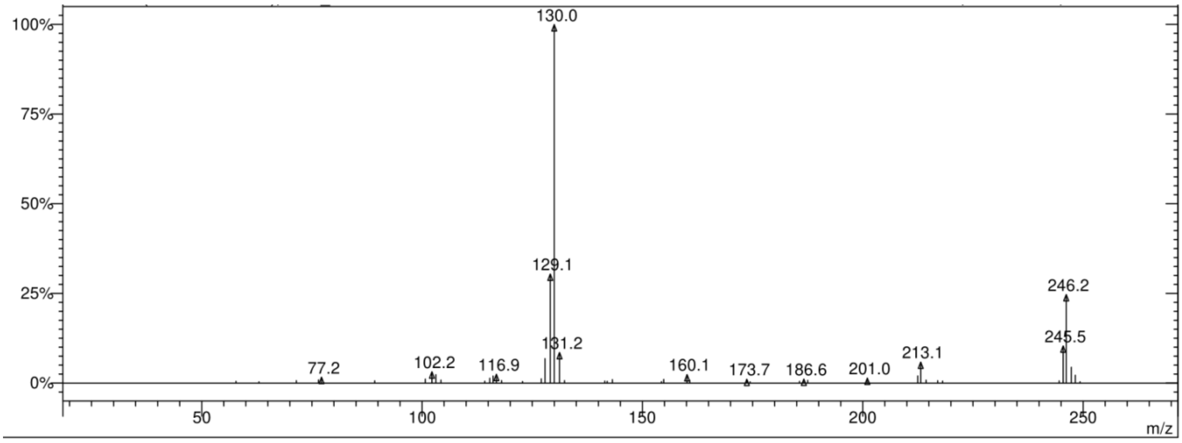

Fig.S9c. EI-MS spectrum of compound **10**

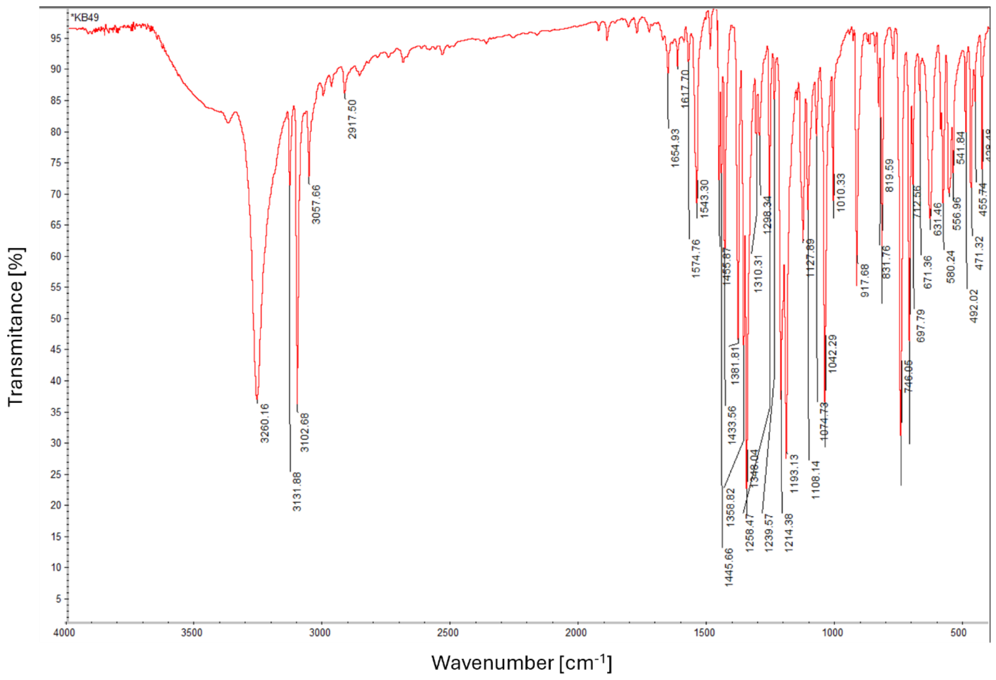

Fig.S9d. IR spectrum of compound **10**

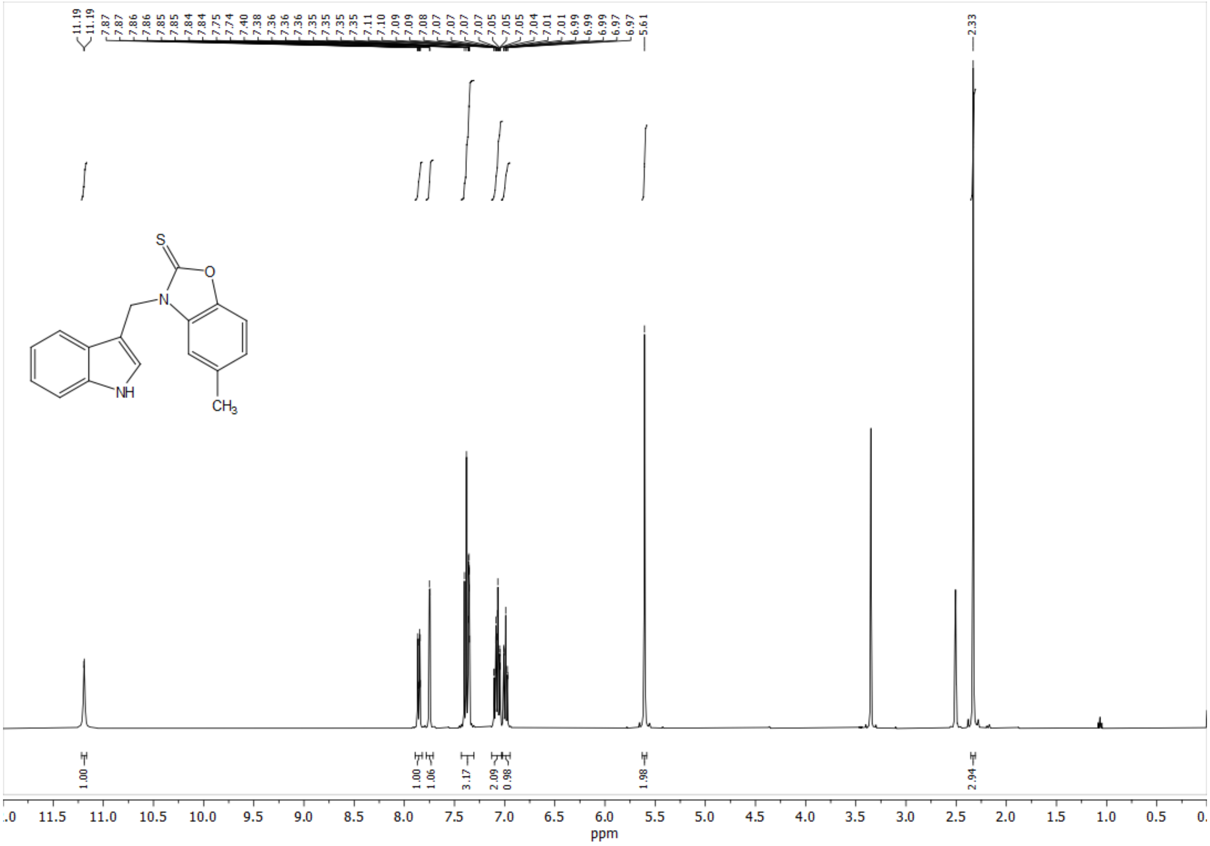

Fig.S10a. ^{1}H NMR spectrum of compound **11**

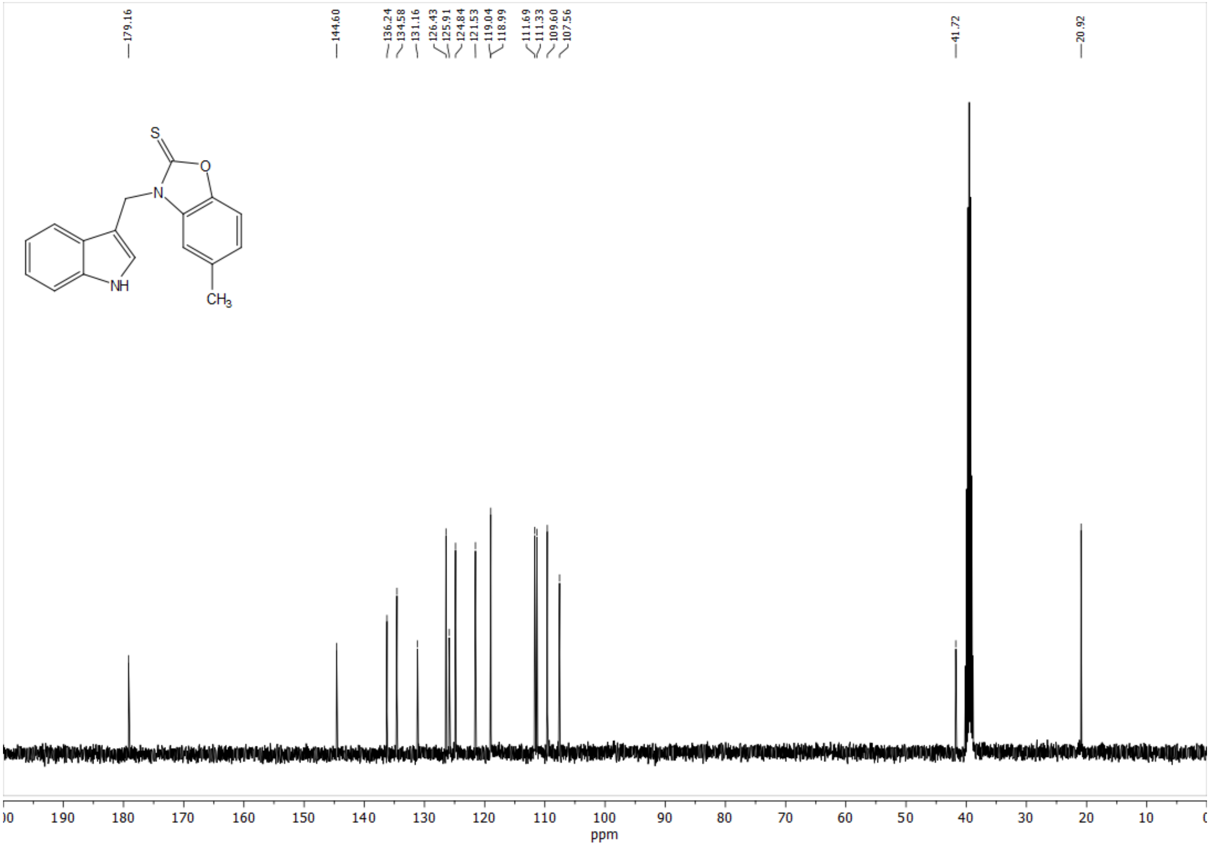

Fig.S10b. ^{13}C NMR spectrum of compound **11**

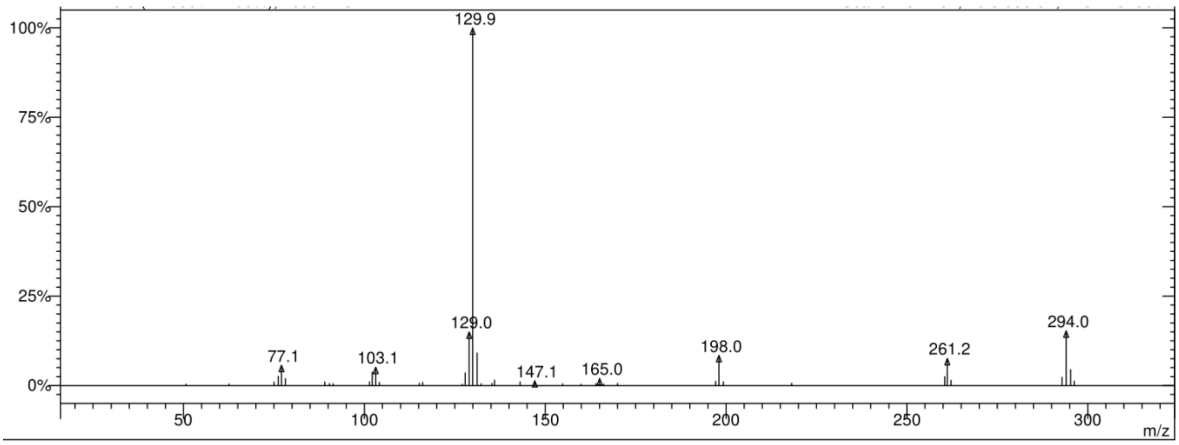

Fig.S10c. EI-MS spectrum of compound **11**

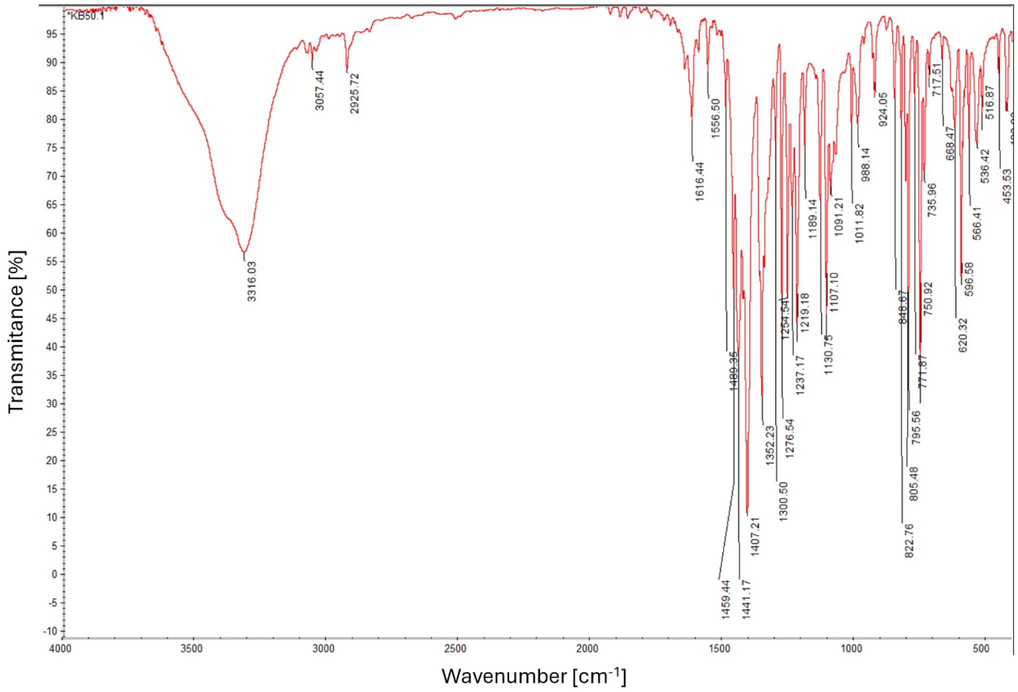

Fig.S10d. IR spectrum of compound **11**

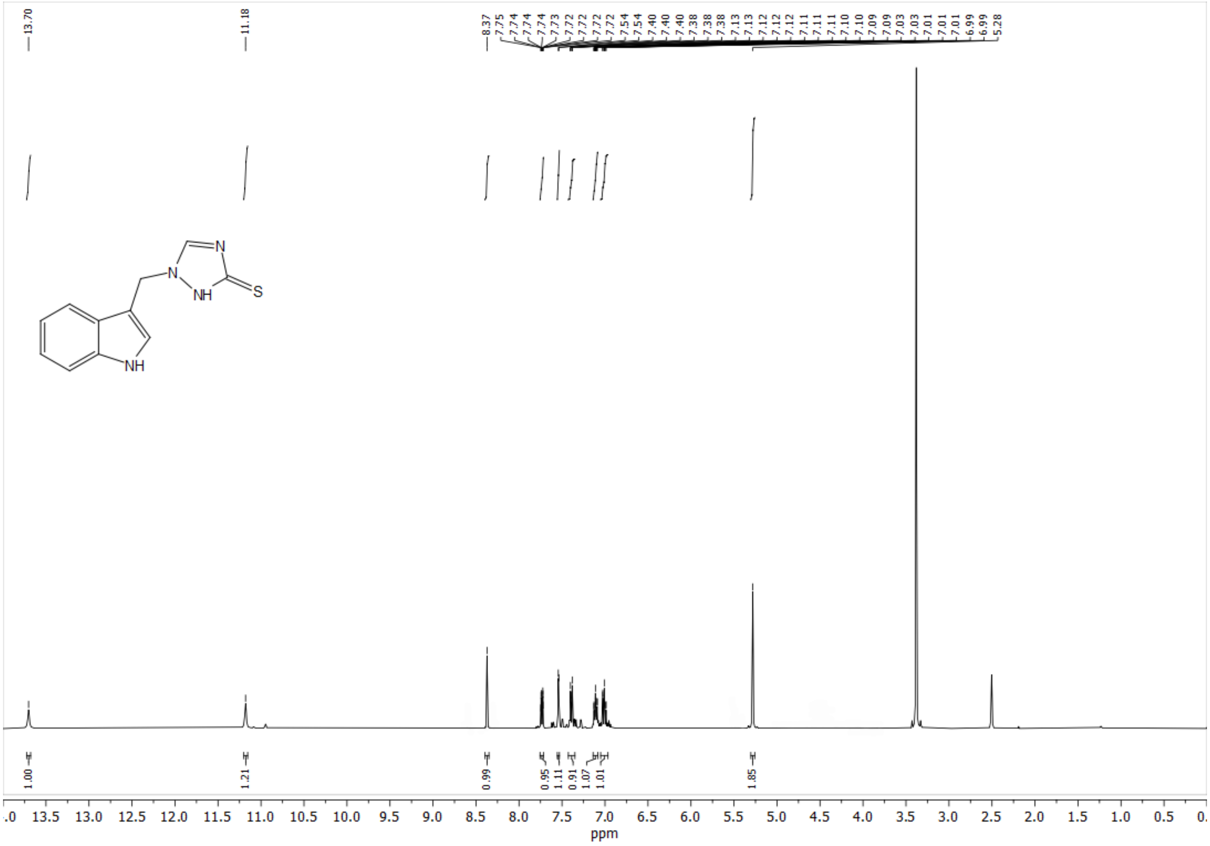

Fig.S11a. ^{1}H NMR spectrum of compound **12**

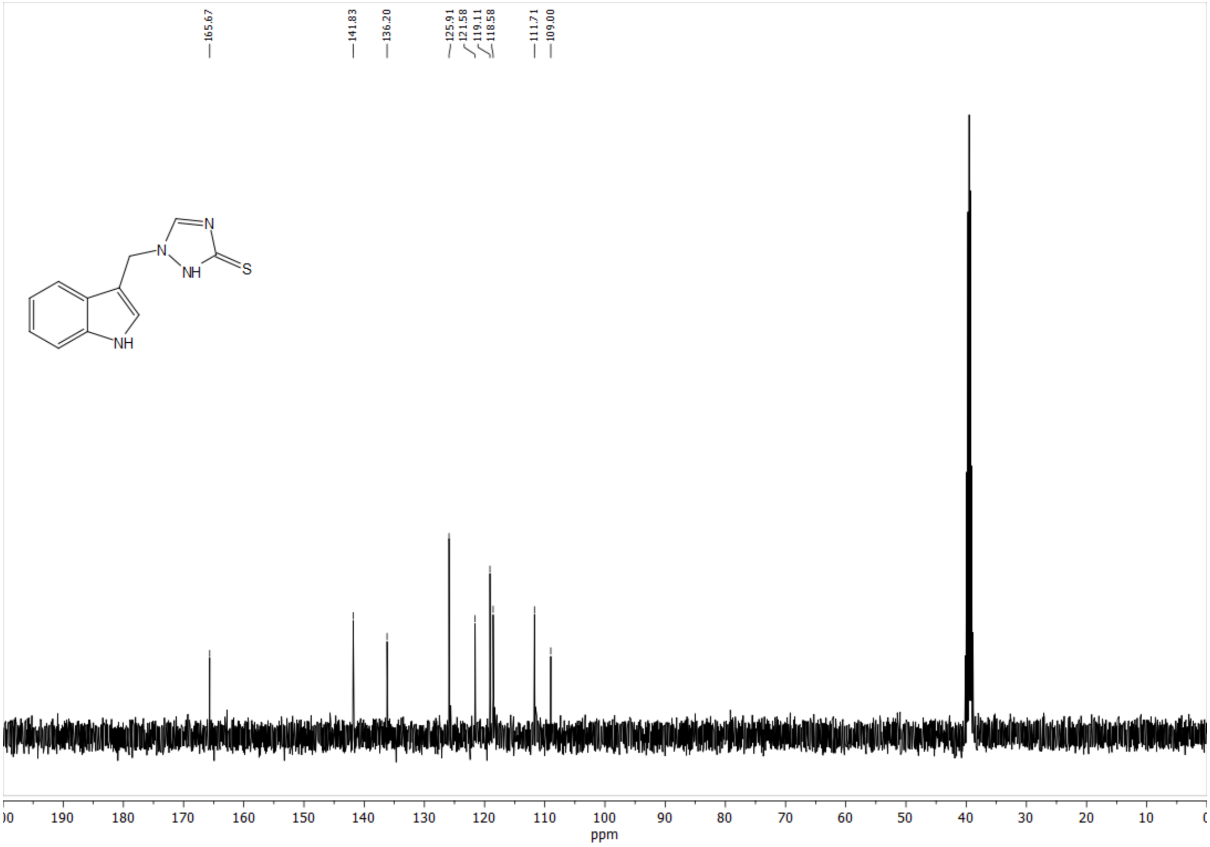

Fig.S11b. ^{13}C NMR spectrum of compound **12**

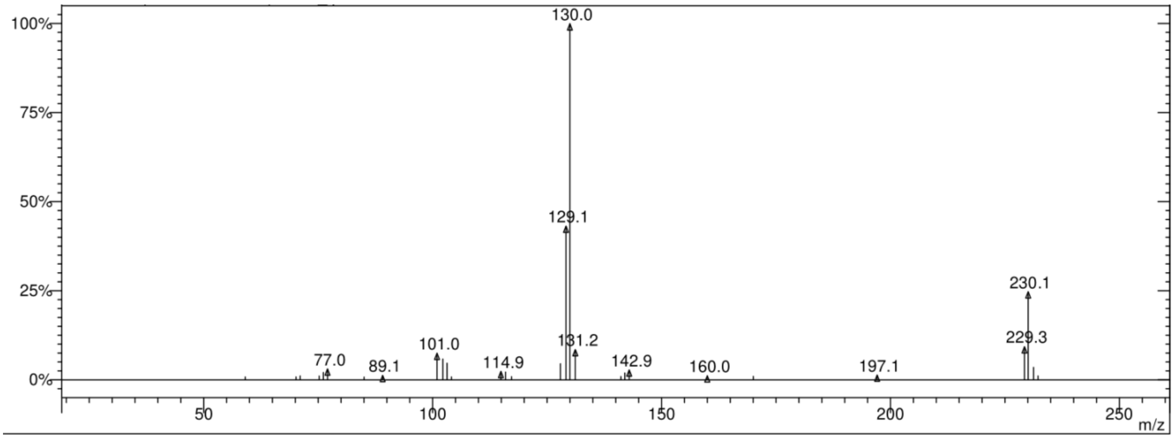

Fig.S11c. EI-MS spectrum of compound **12**

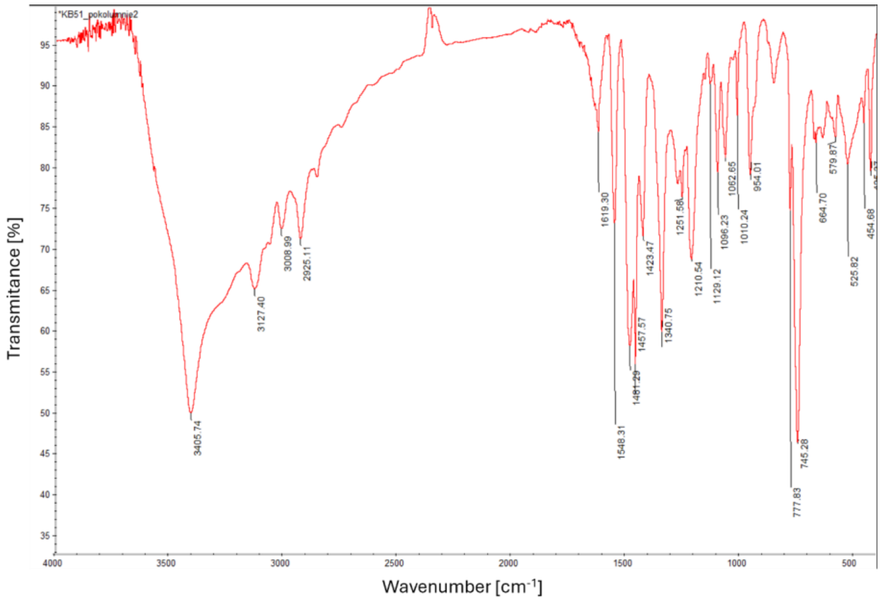

Fig.S11d. IR spectrum of compound **12**

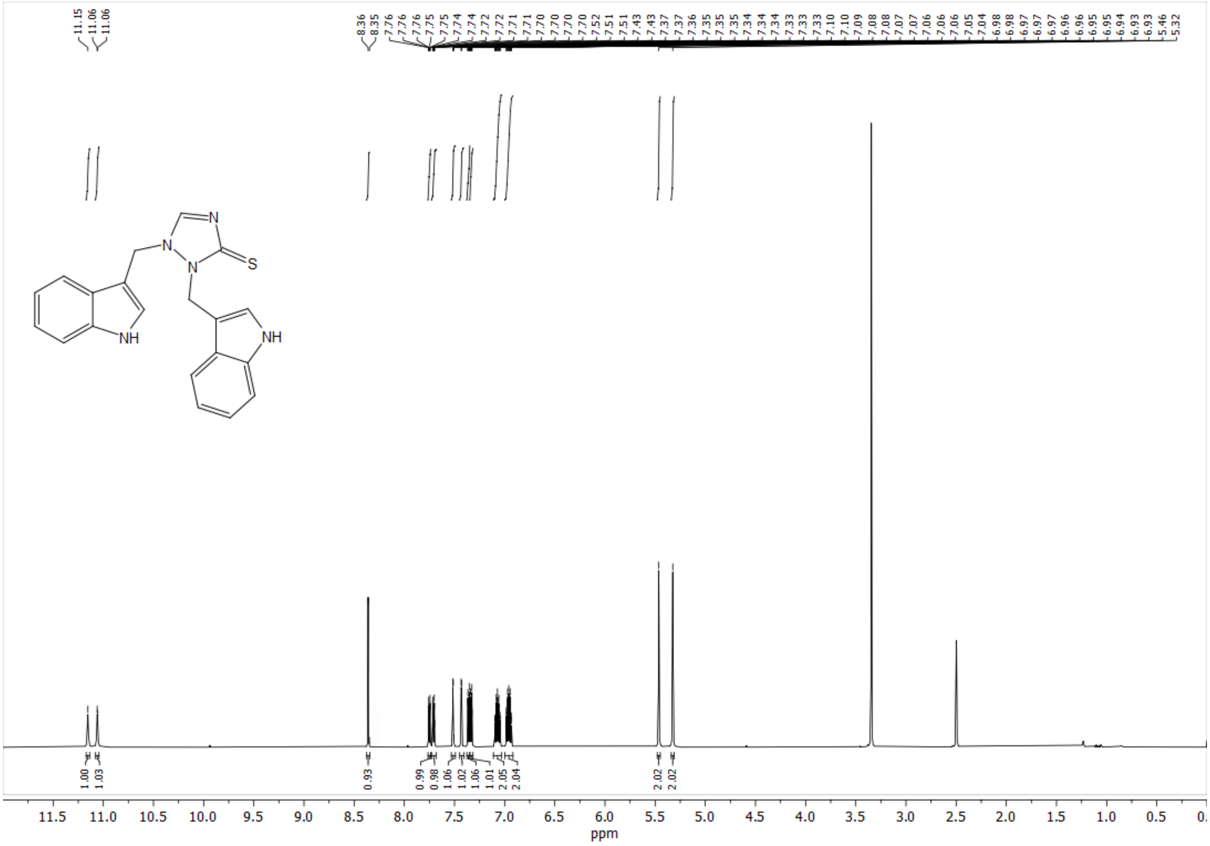

Fig.S12a. ^{1}H NMR spectrum of compound **13**

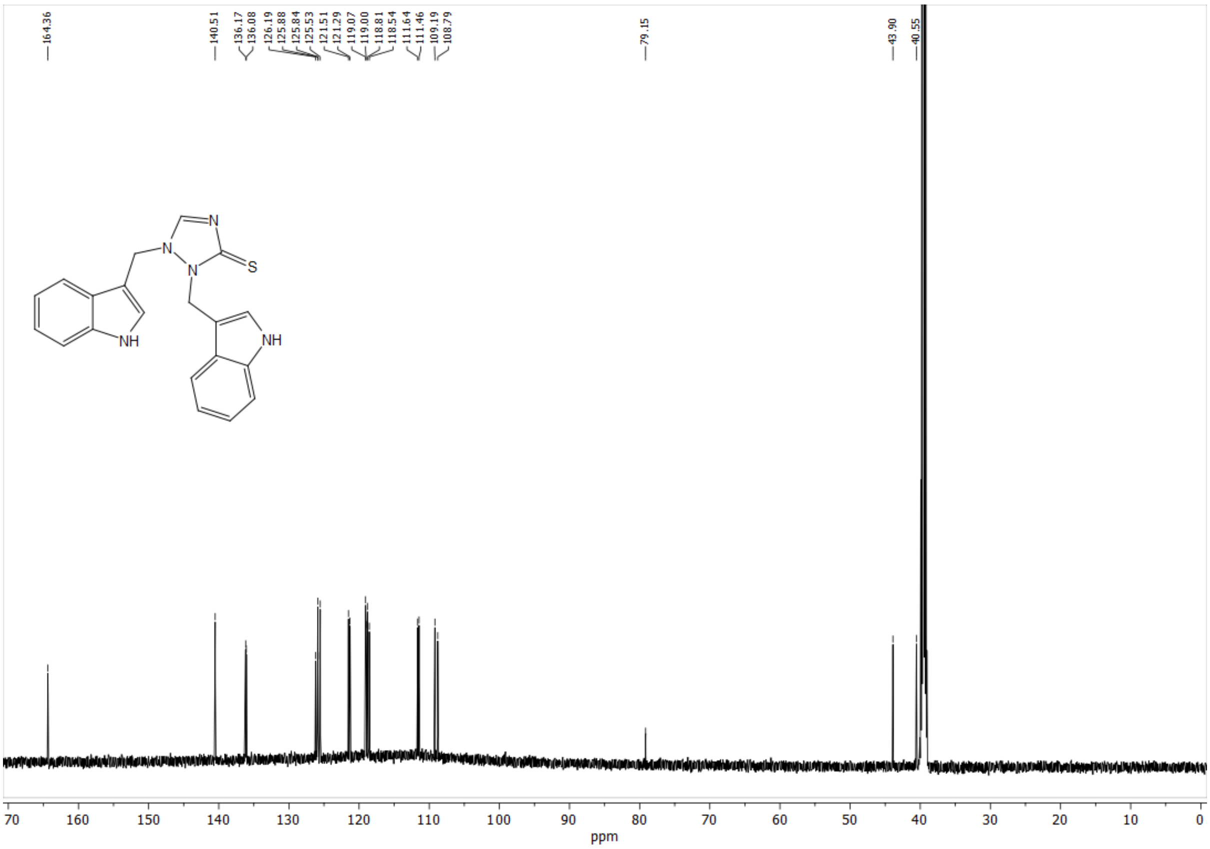

Fig.S12b. ^{13}C NMR spectrum of compound **13**

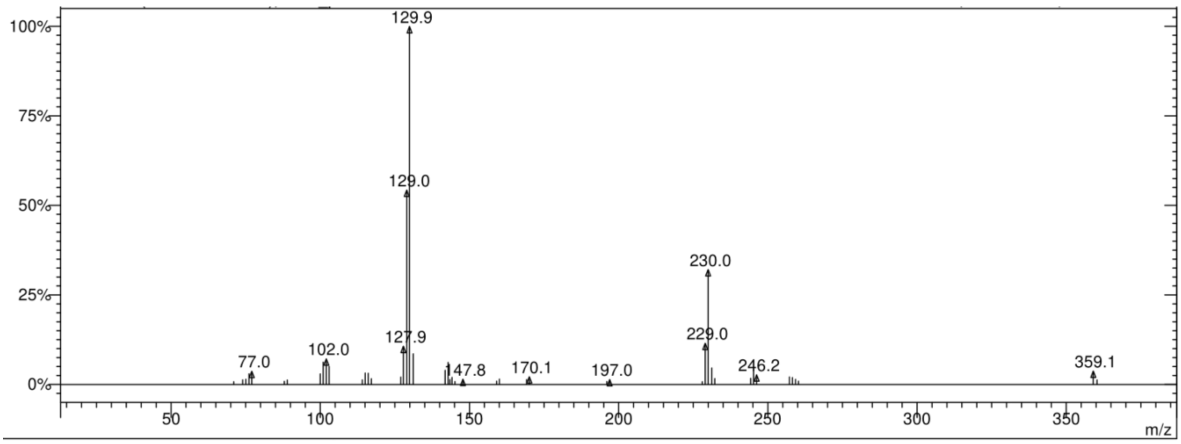

Fig.S12c. EI-MS spectrum of compound **13**

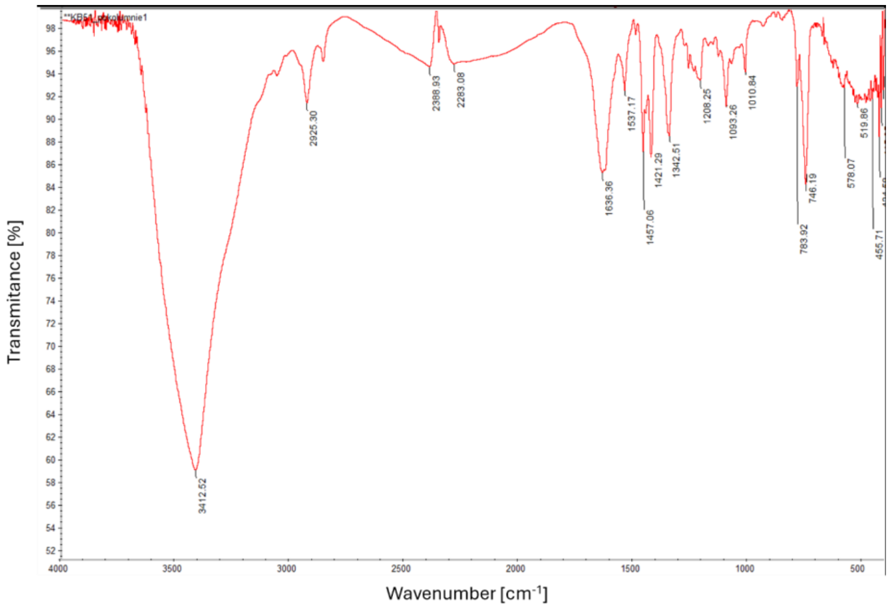

Fig.S12d. IR spectrum of compound **13**

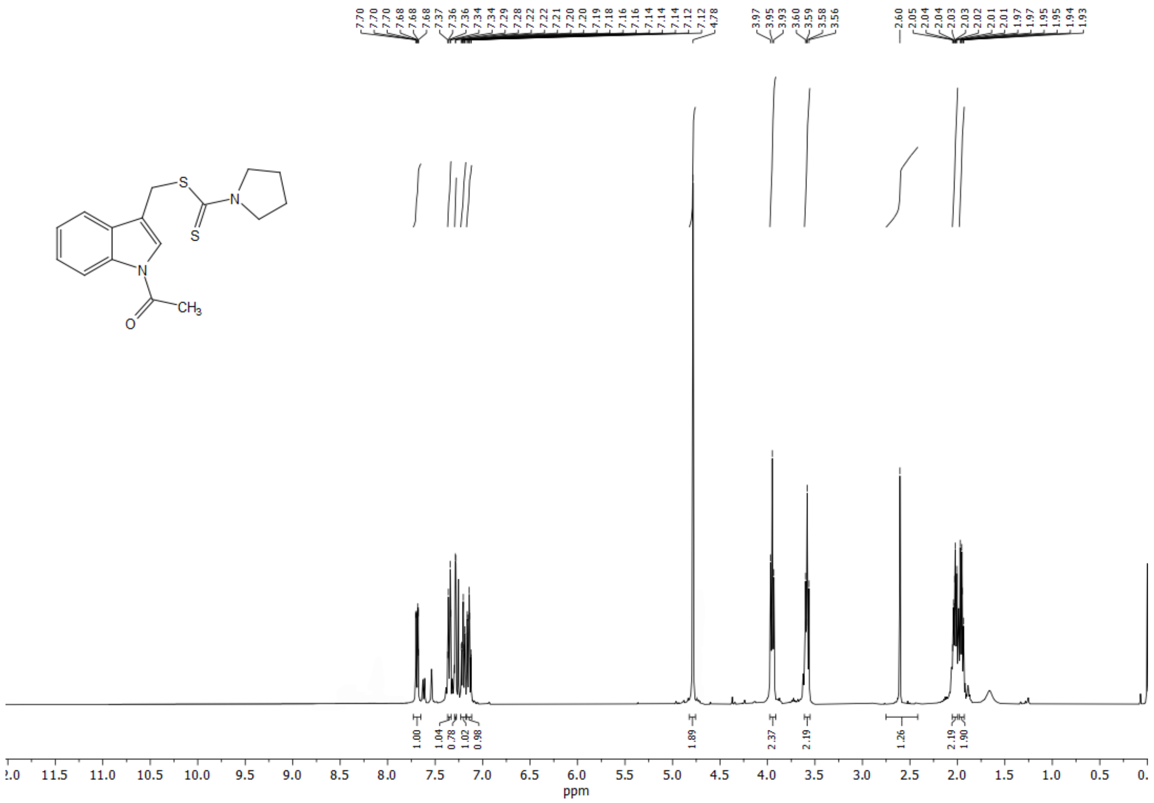

Fig.S13a. ^{1}H NMR spectrum of compound **15**

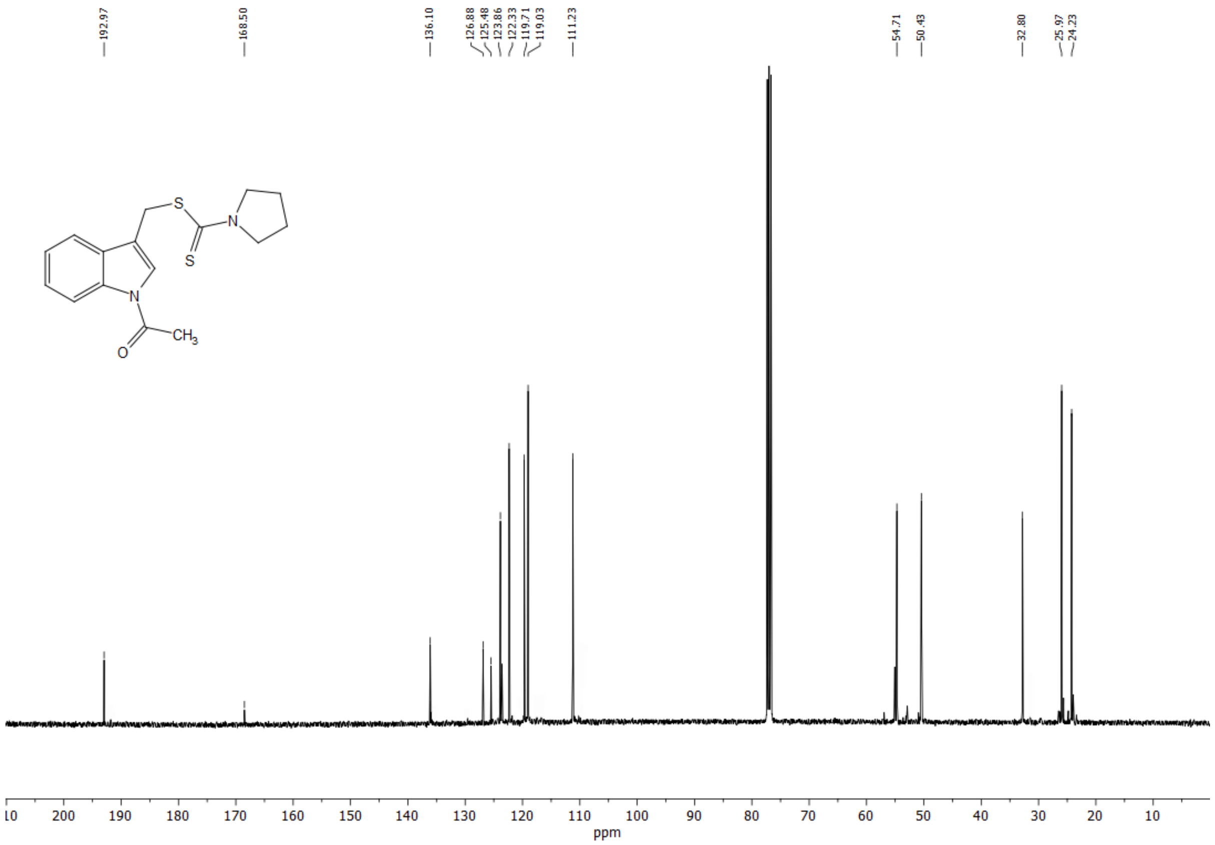

Fig.S13b. ^{13}C NMR spectrum of compound **15**

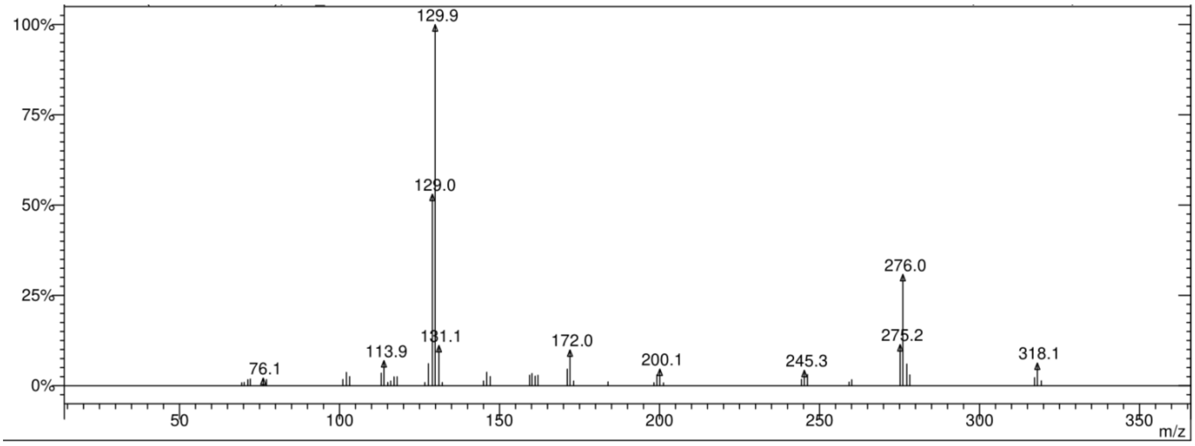

Fig.S13c. EI-MS spectrum of compound **15**

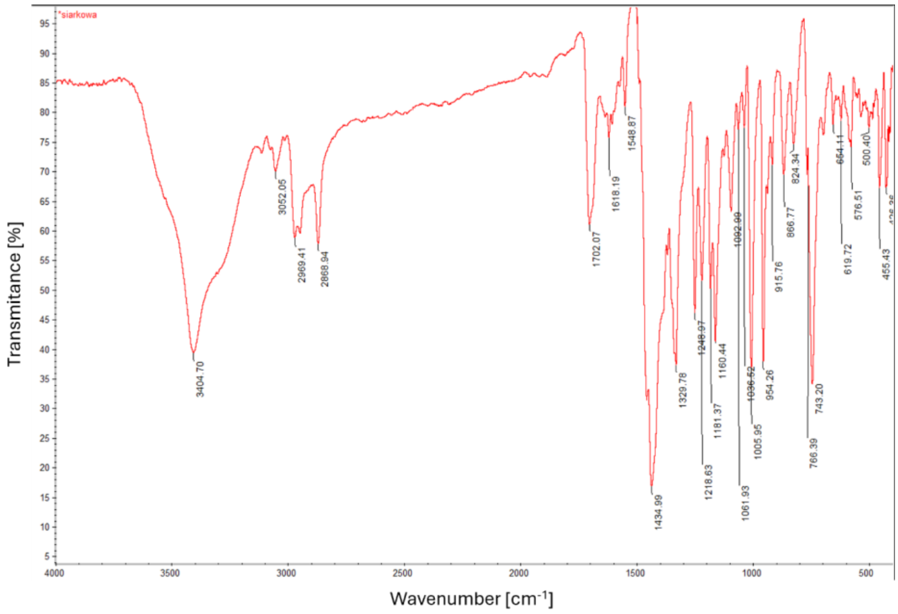

Fig.S13d. FT-IR spectrum of compound **15**

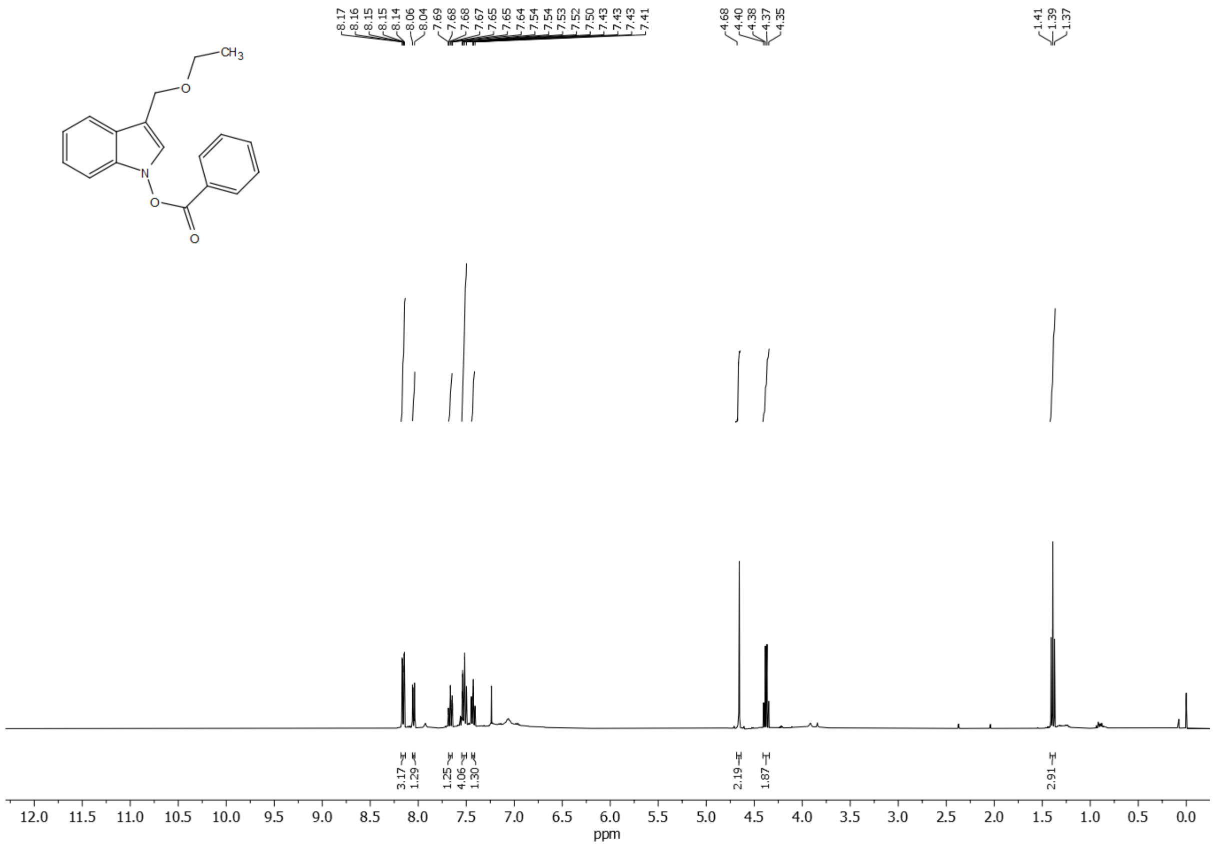

Fig.S14a. ^{1}H NMR spectrum of compound **17**

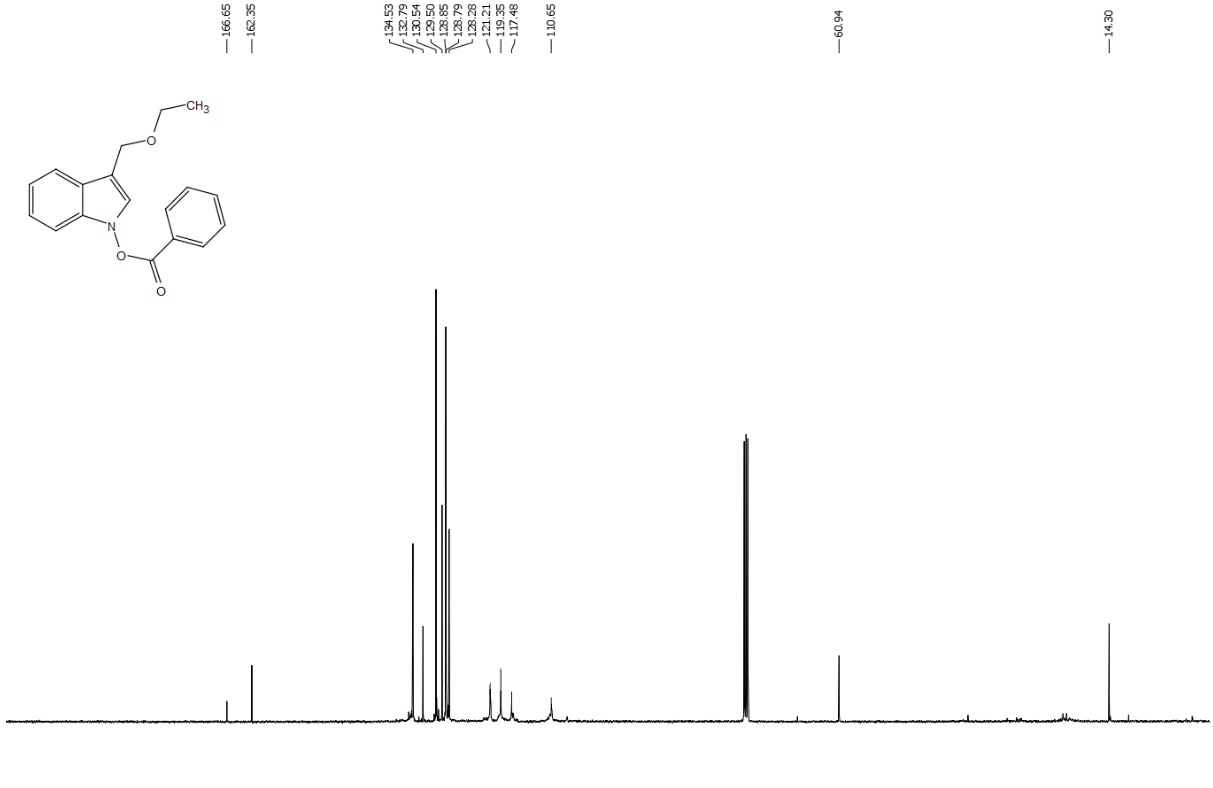

Fig.S14b. ^{13}C NMR spectrum of compound **17**

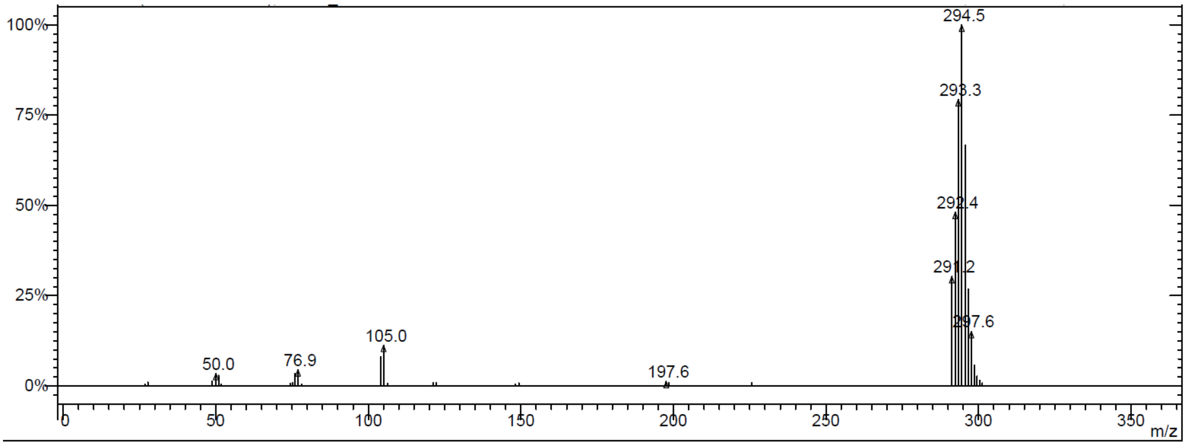

Fig.S14c. EI-MS spectrum of compound **17**

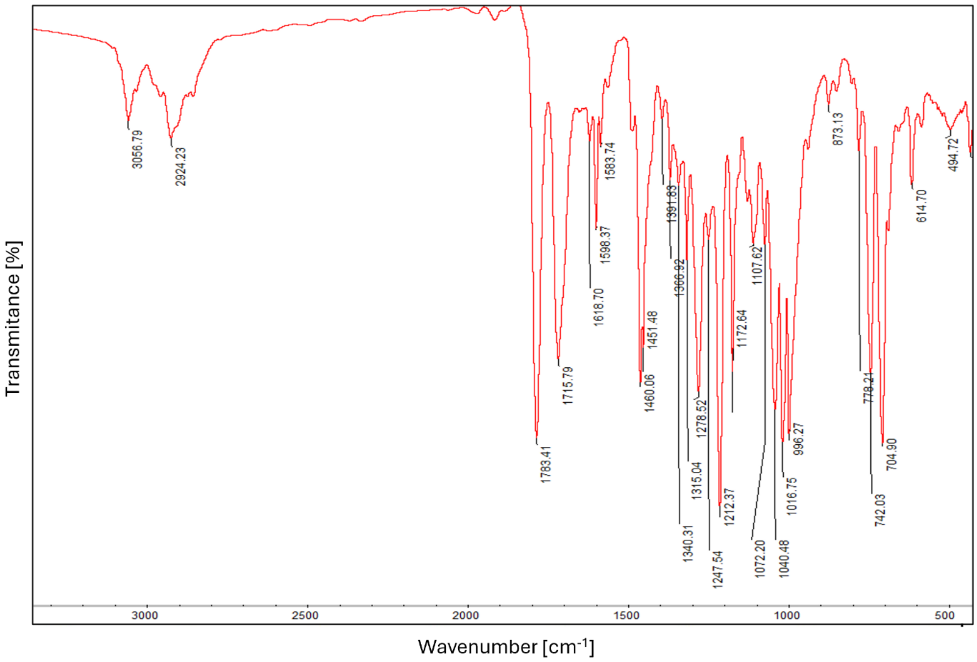

Fig.S14d. FT-IR spectrum of compound **17**

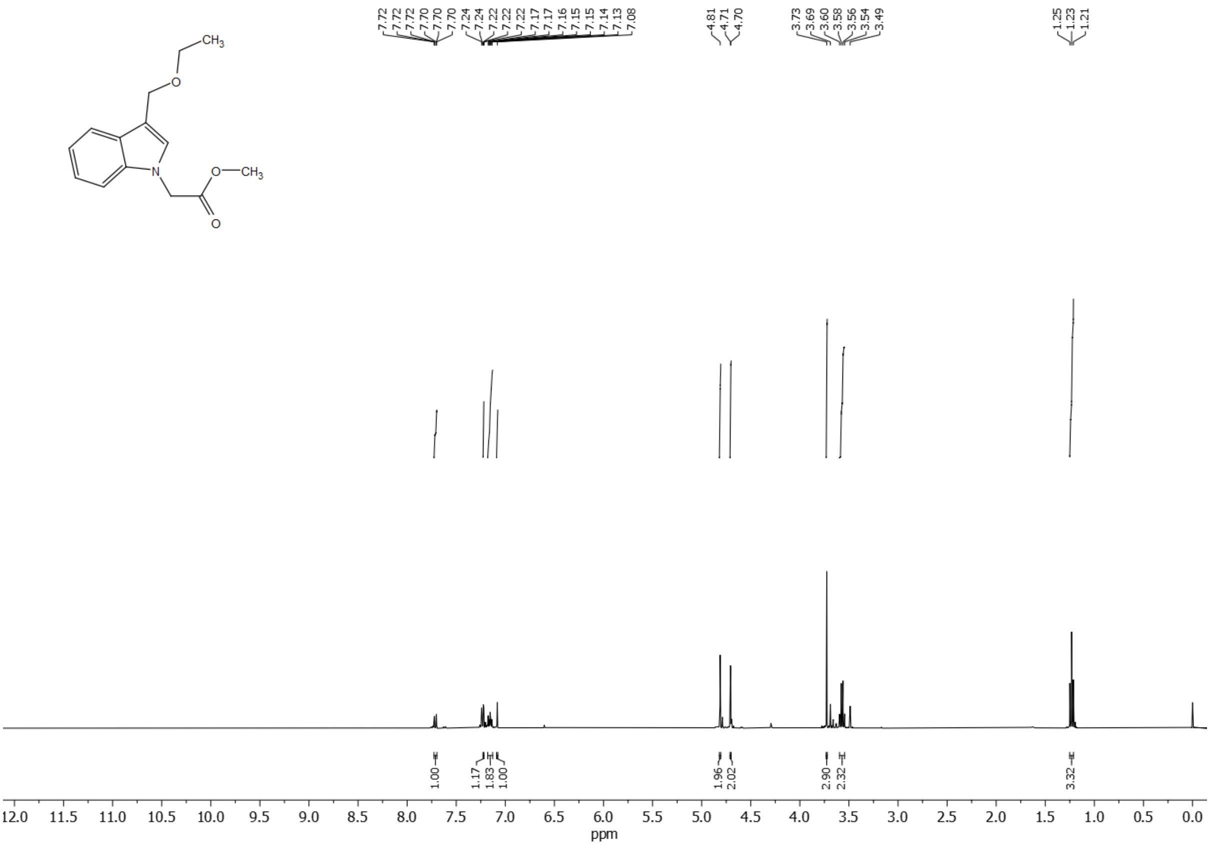

Fig.S15a. ^{1}H NMR spectrum of compound **18**

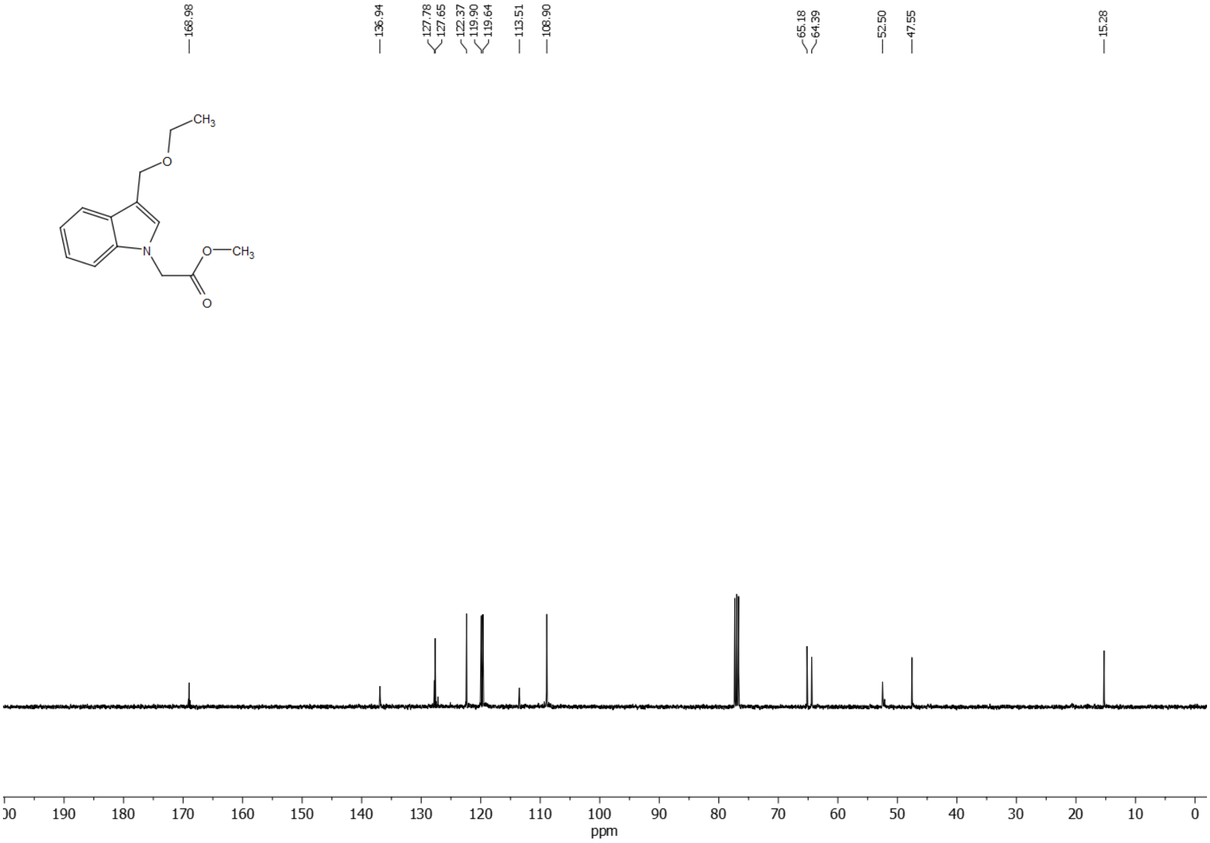

Fig.S15b. ^{13}C NMR spectrum of compound **18**

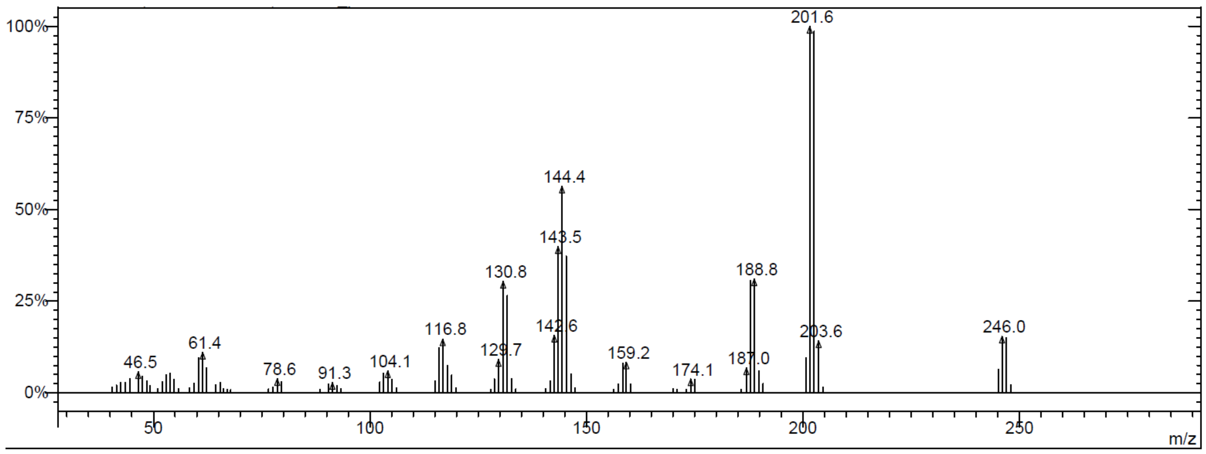

Fig.S15c. EI-MS spectrum of compound **18**

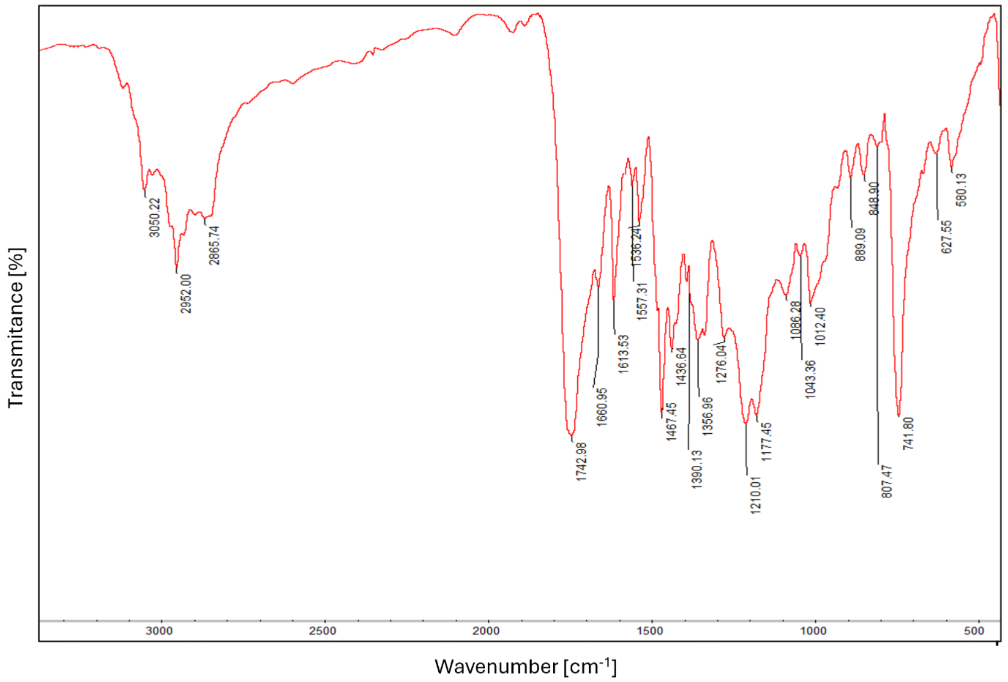

Fig.S15d. FT-IR spectrum of compound **18**

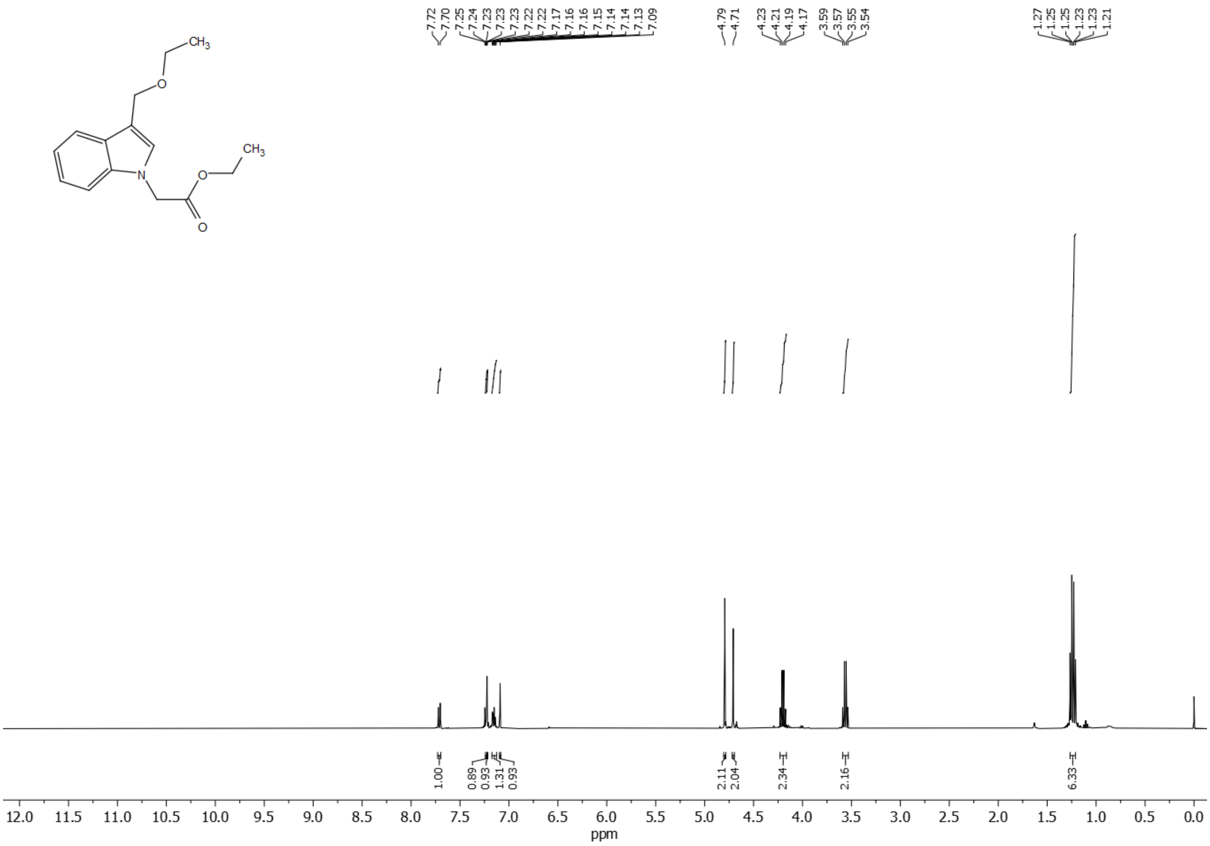

Fig.S16a. ^{1}H NMR spectrum of compound **19**

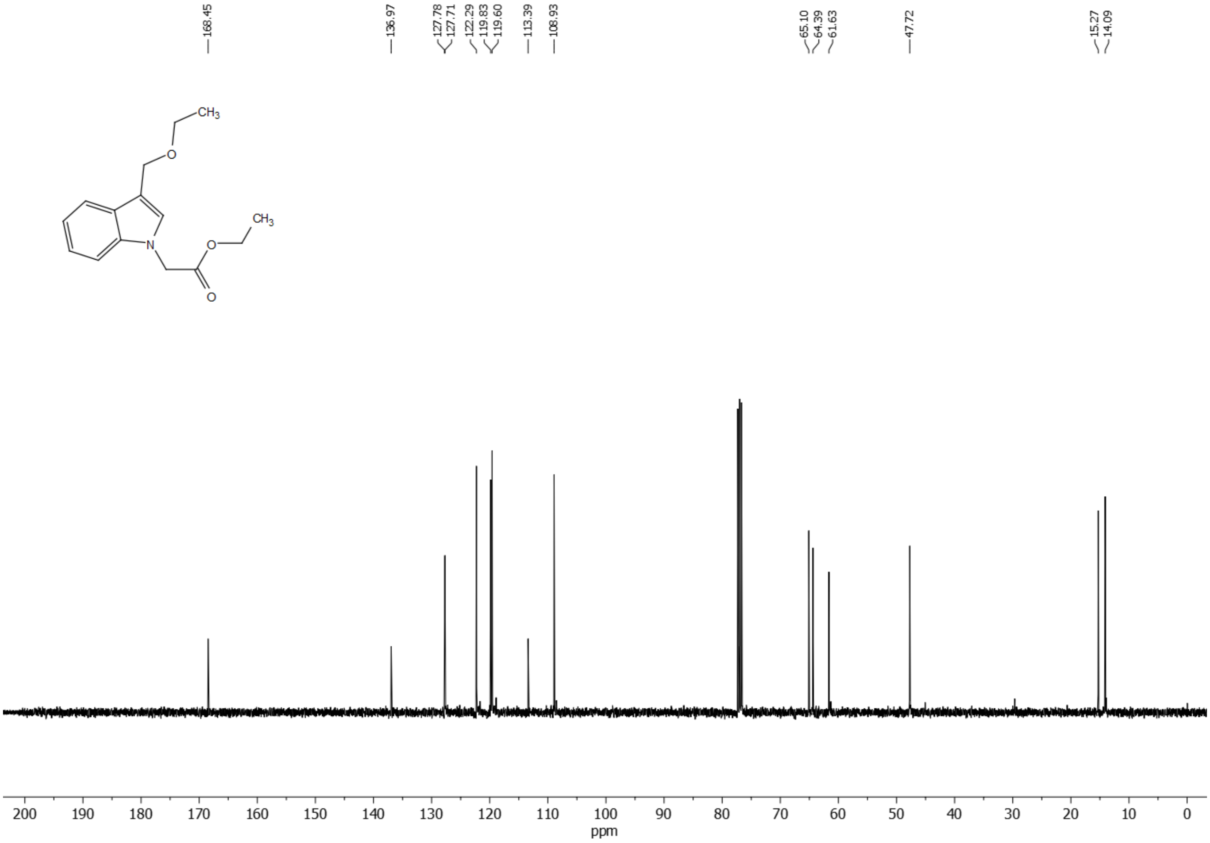

Fig.S16b. ^{13}C NMR spectrum of compound **19**

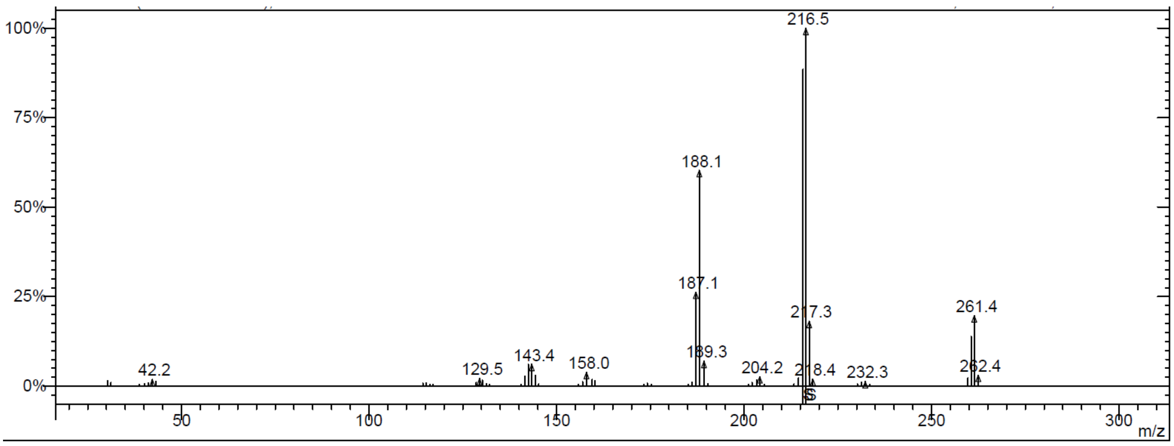

Fig.S16c. EI-MS spectrum of compound **19**

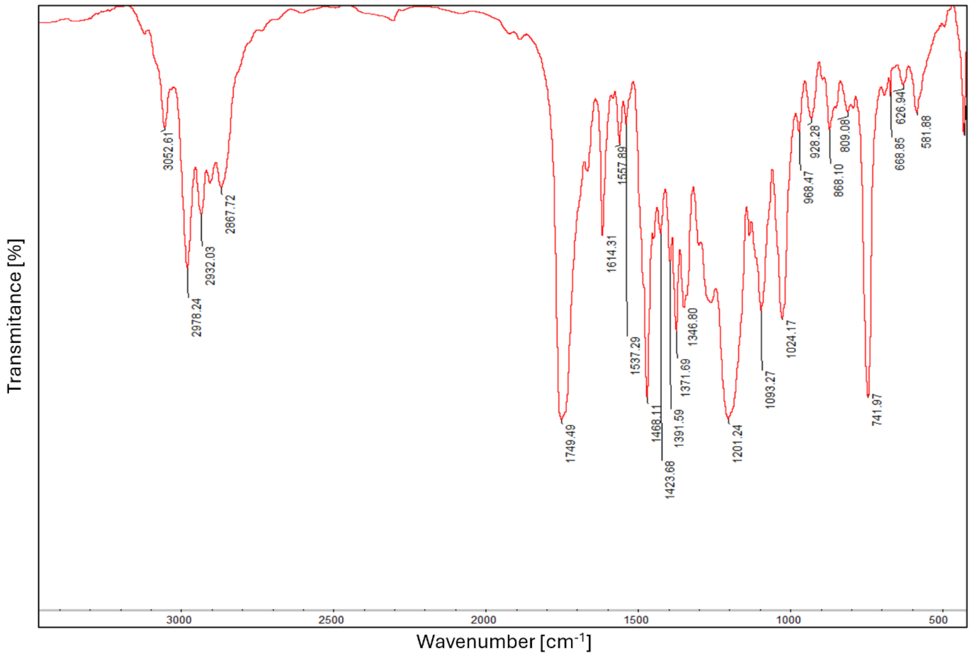

Fig.S16d. FT-IR spectrum of compound **19**

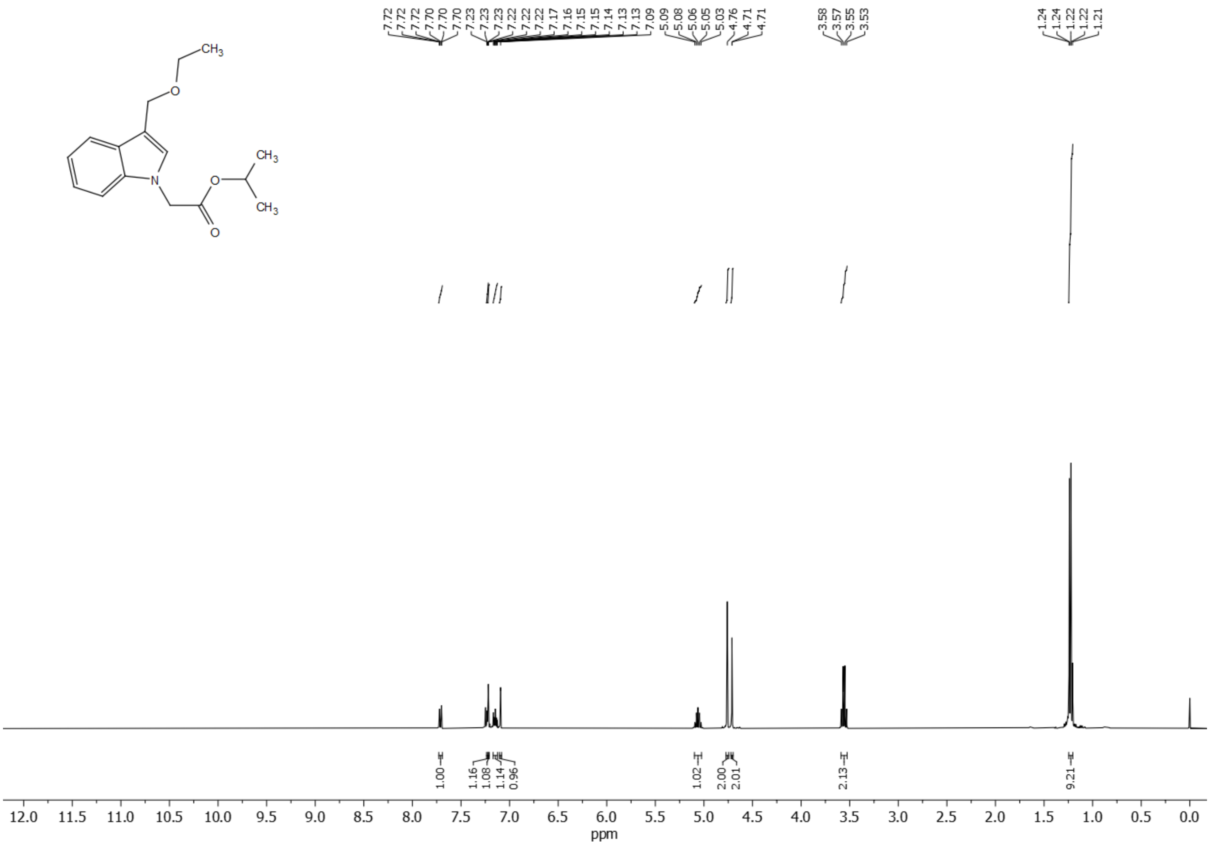

Fig.S17a. ^{1}H NMR spectrum of compound **20**

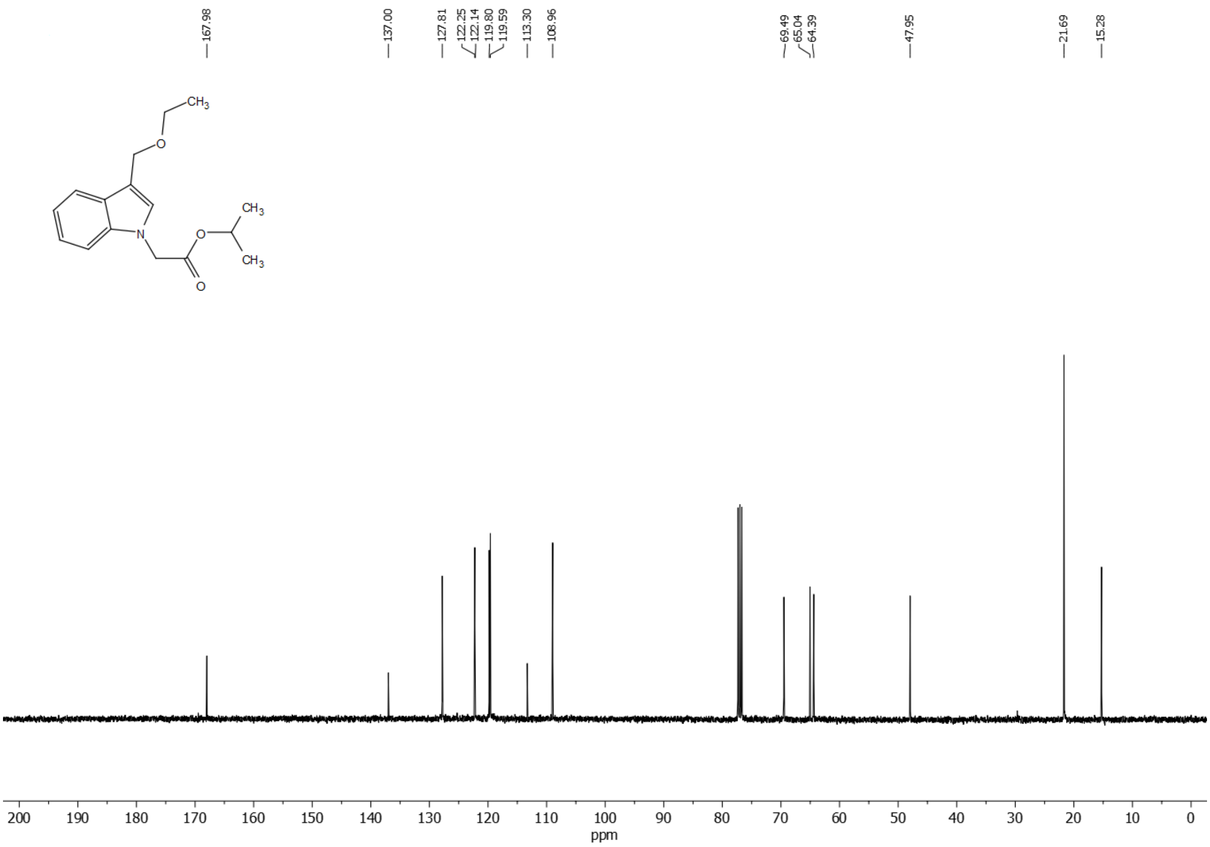

Fig.S17b. ^{13}C NMR spectrum of compound **20**

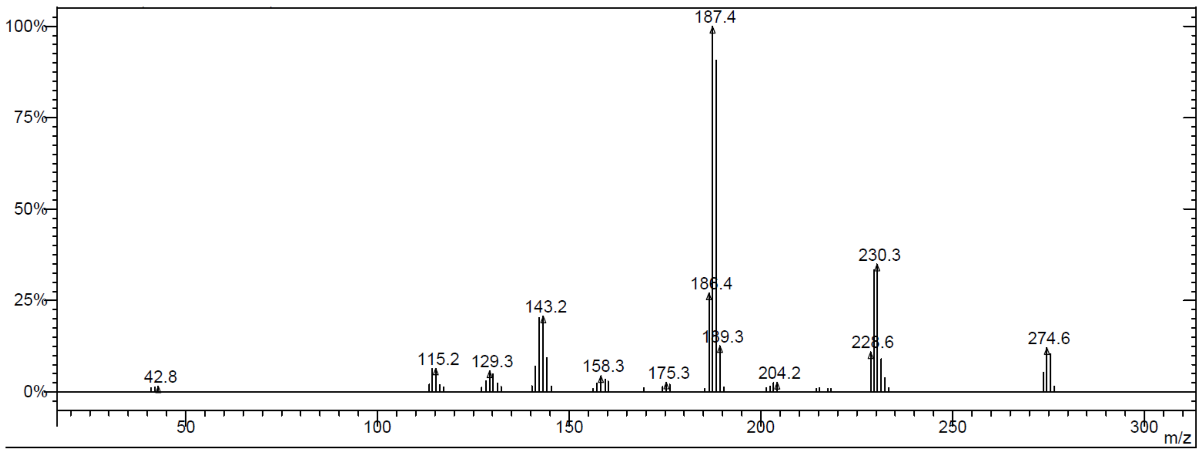

Fig.S17c. EI-MS spectrum of compound **20**

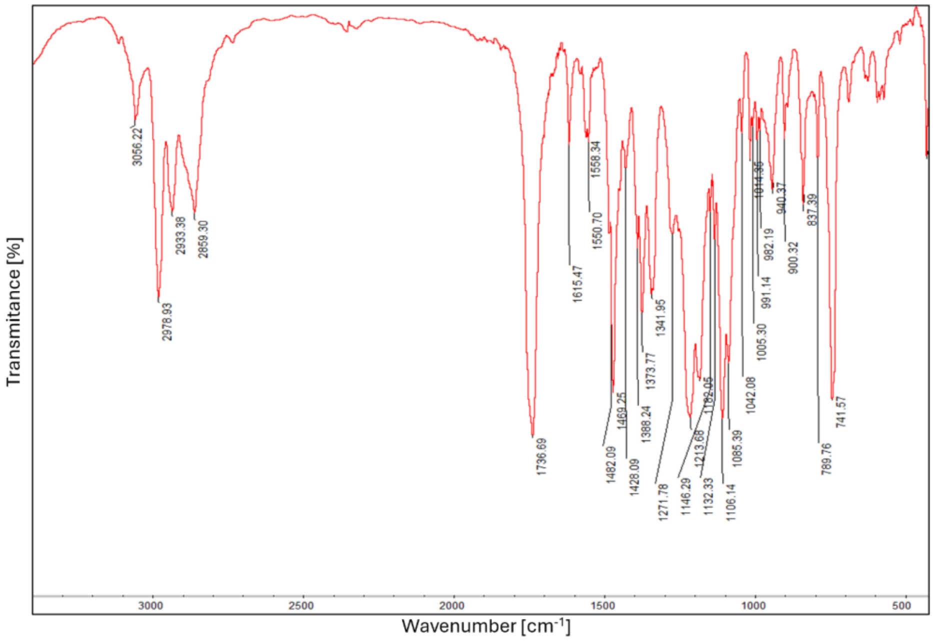

Fig.S17d. FT-IR spectrum of compound **20**

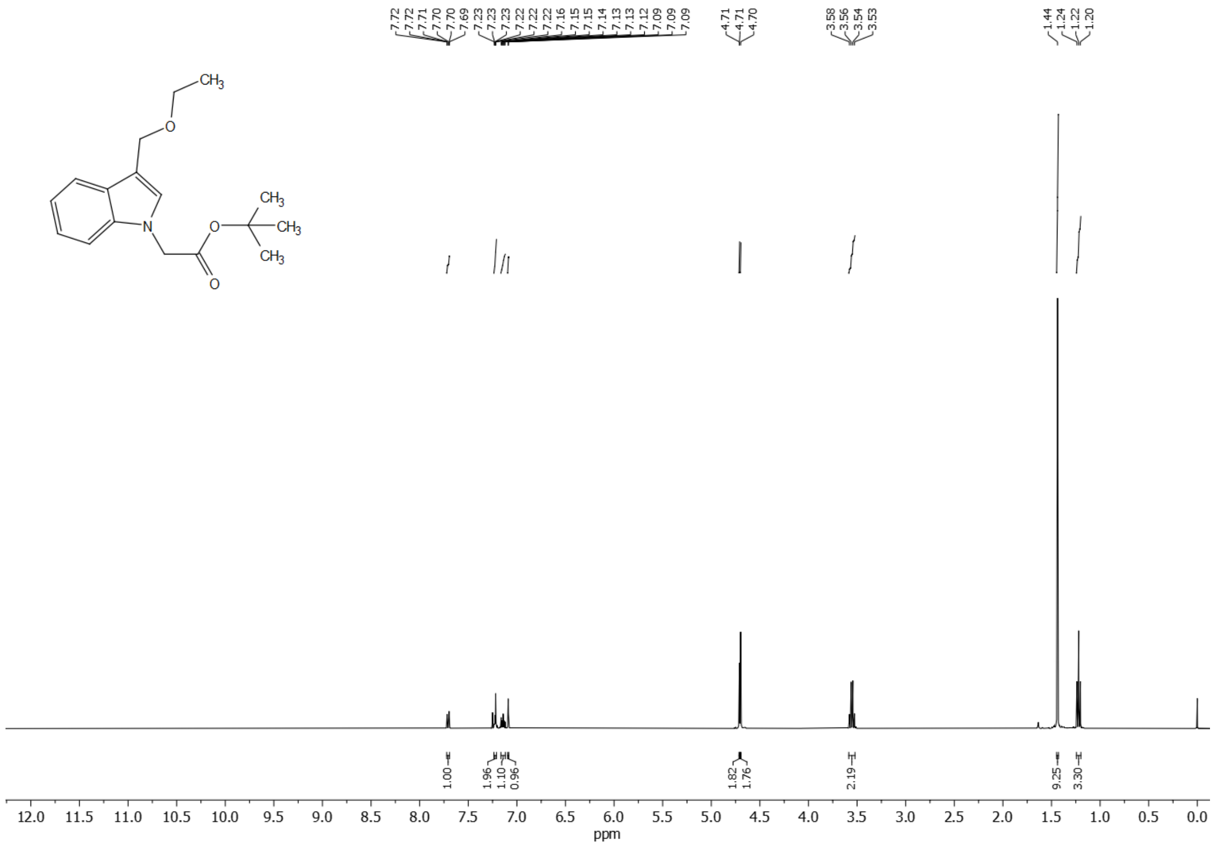

Fig.S18a. ^{1}H NMR spectrum of compound **21**

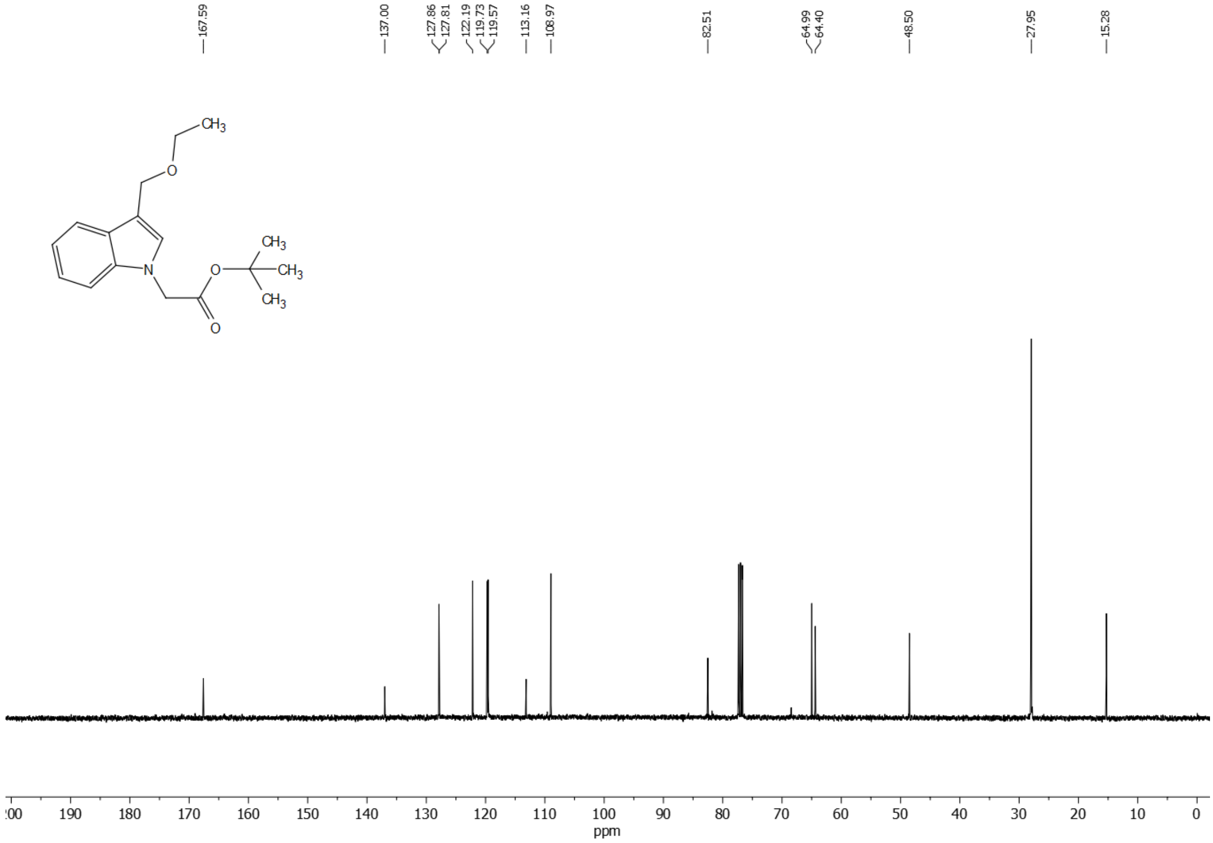

Fig.S18b. ^{13}C NMR spectrum of compound **21**

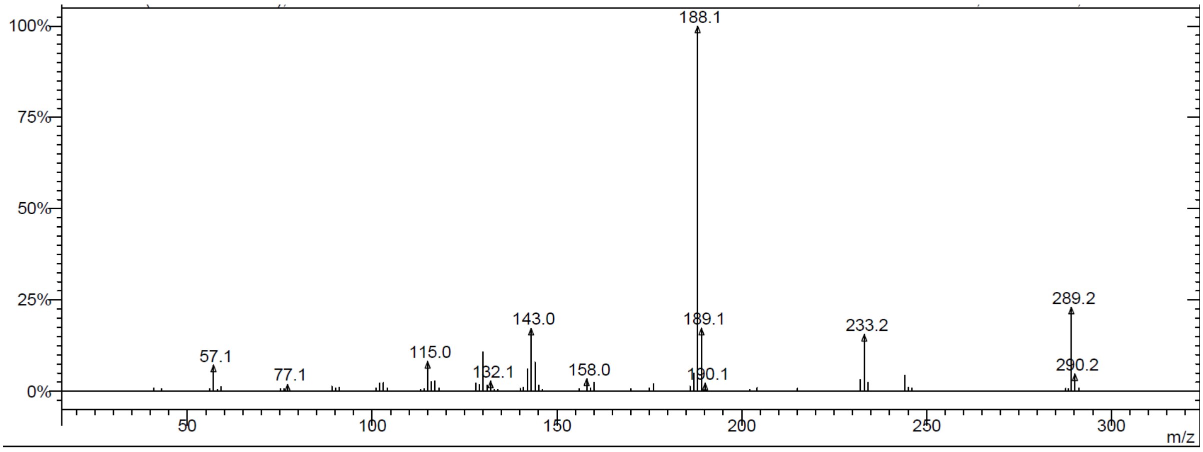

Fig.S18c. EI-MS spectrum of compound **21**

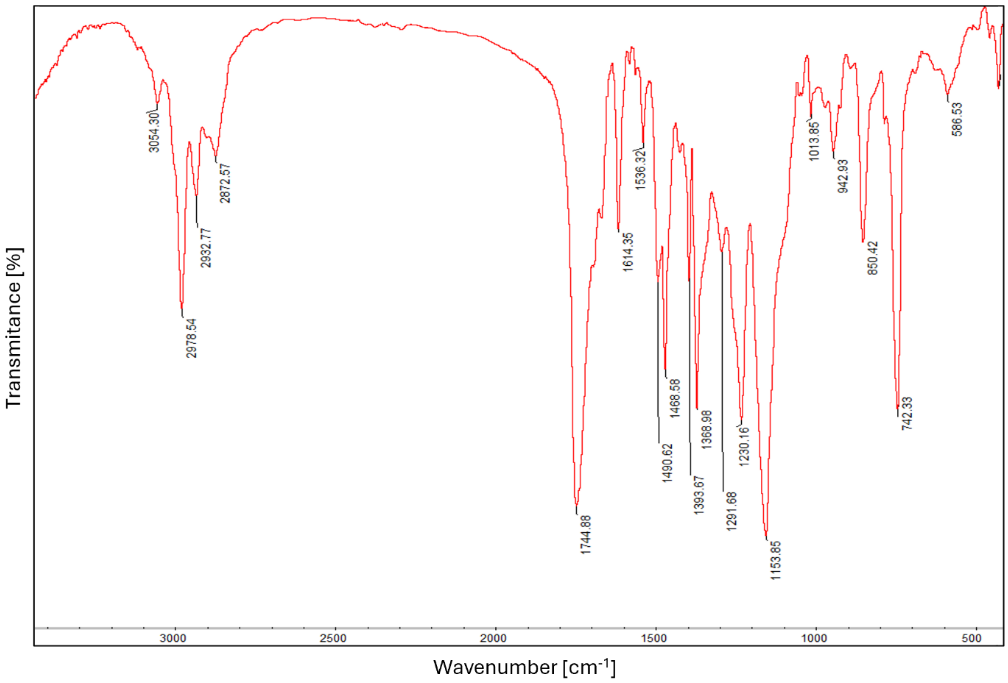

Fig.S18d. FT-IR spectrum of compound **21**

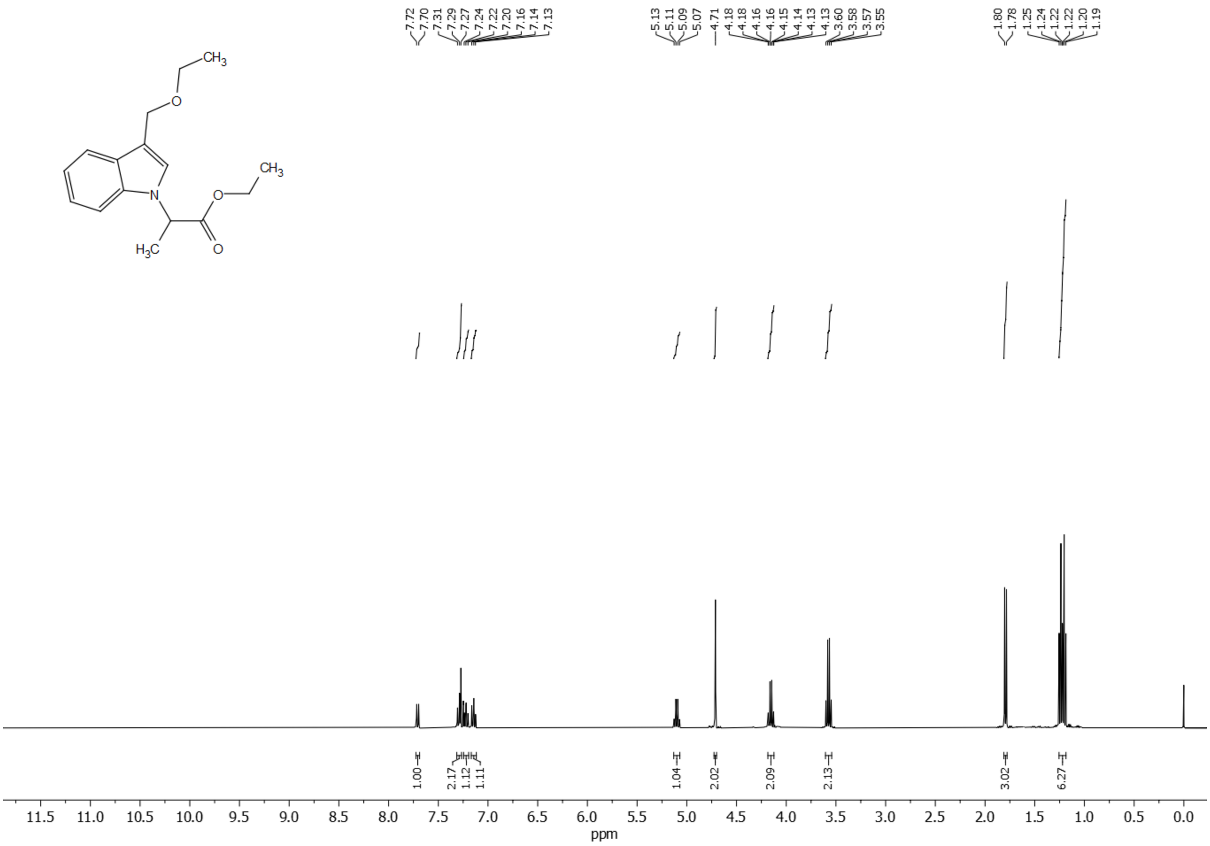

Fig.S19a. ^{1}H NMR spectrum of compound **22**

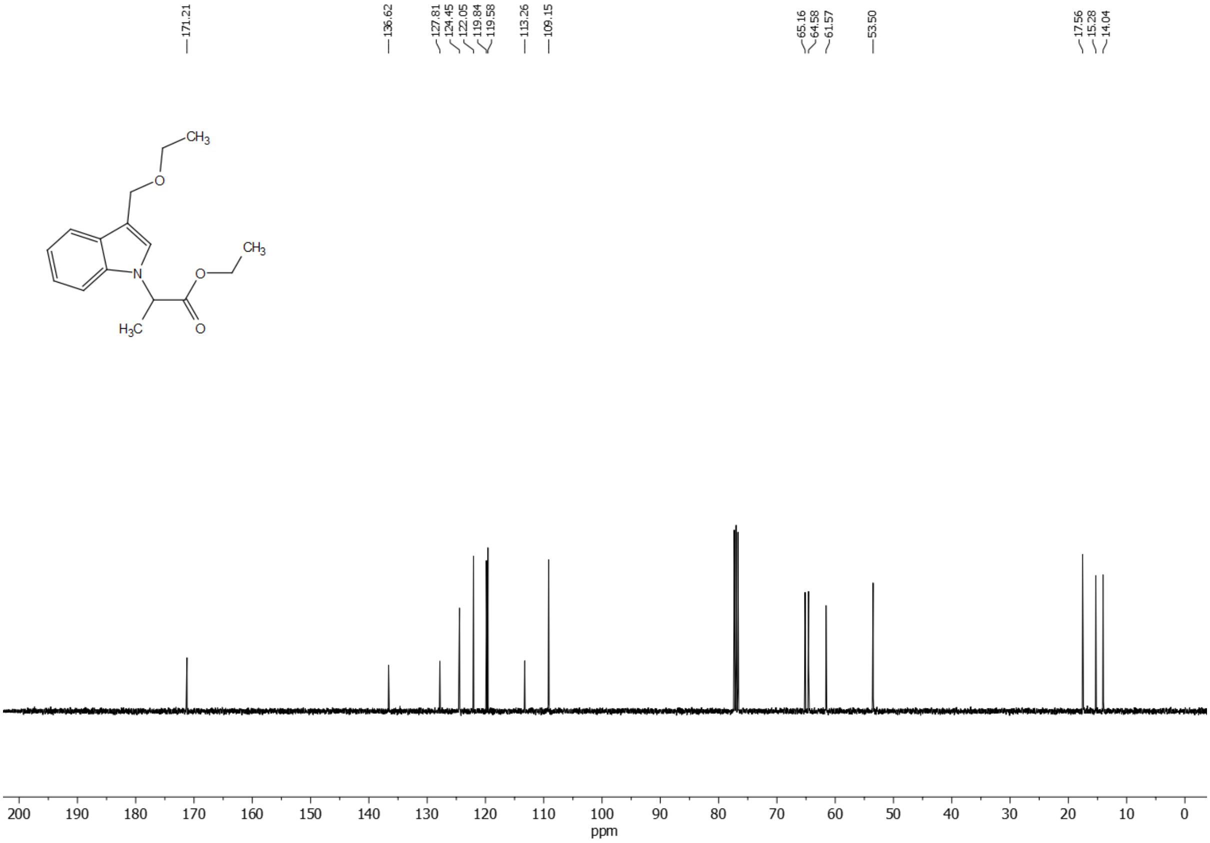

Fig.S19b. ^{13}C NMR spectrum of compound **22**

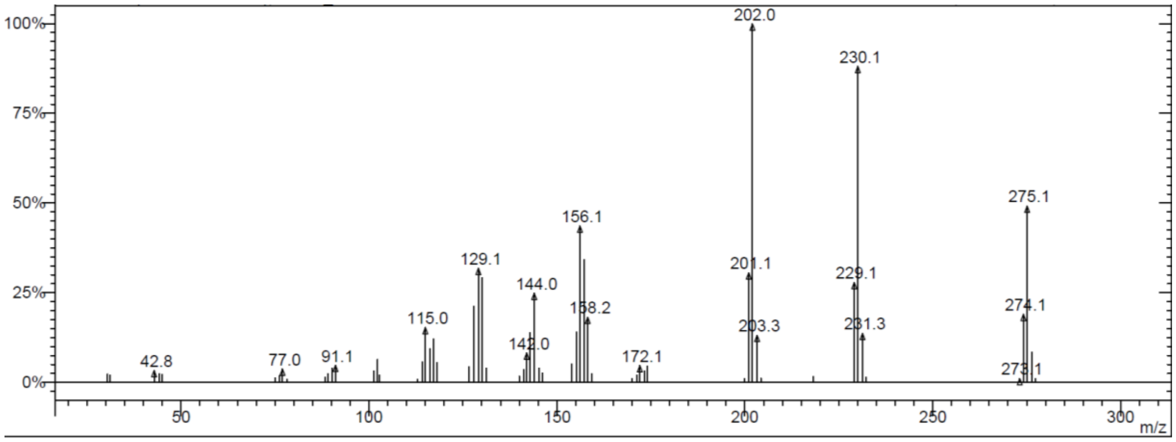

Fig.S19c. EI-MS spectrum of compound **22**

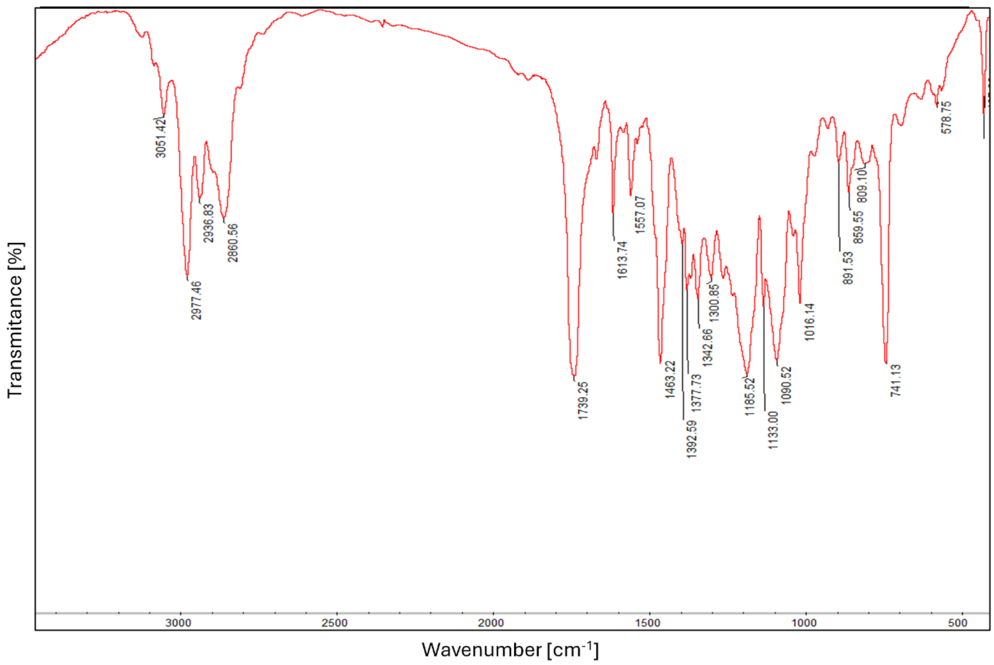

Fig.S19d. FT-IR spectrum of compound **22**

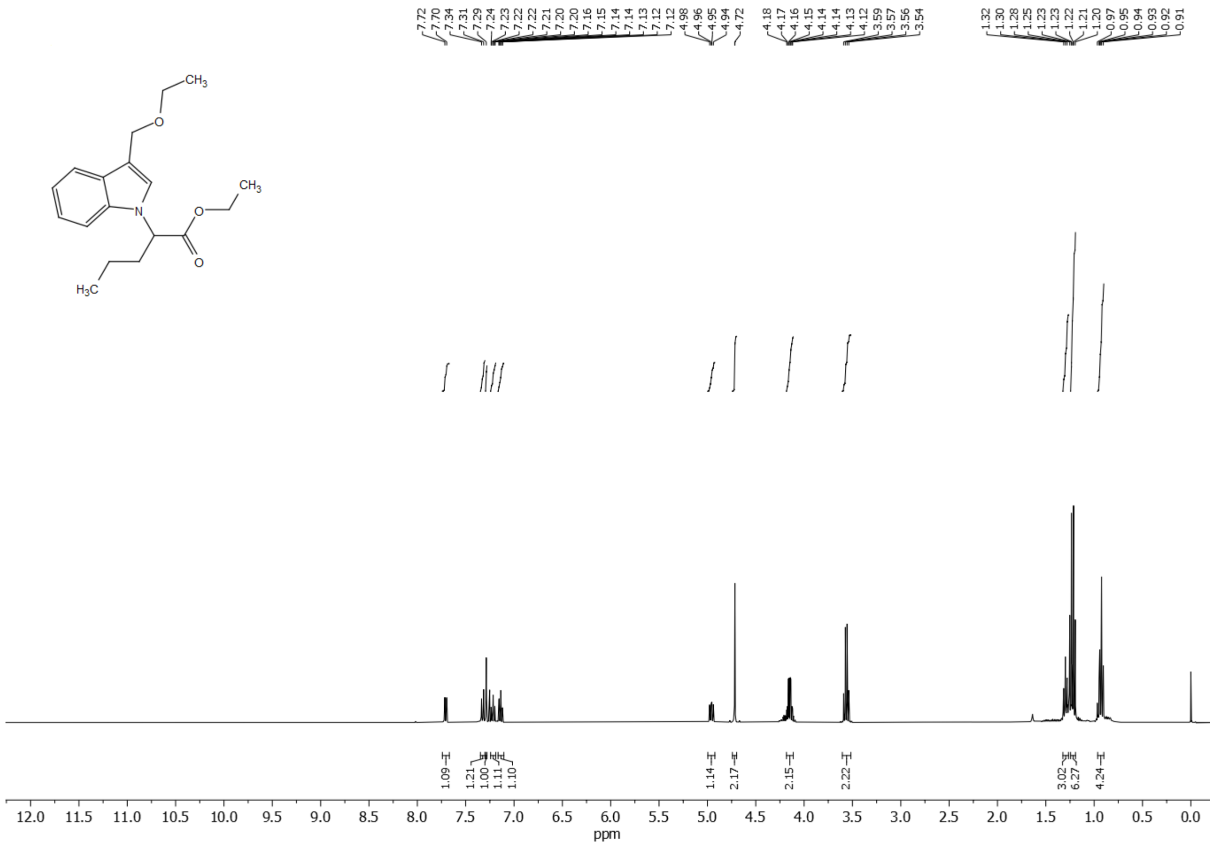

Fig.S20a. ^{1}H NMR spectrum of compound **23**

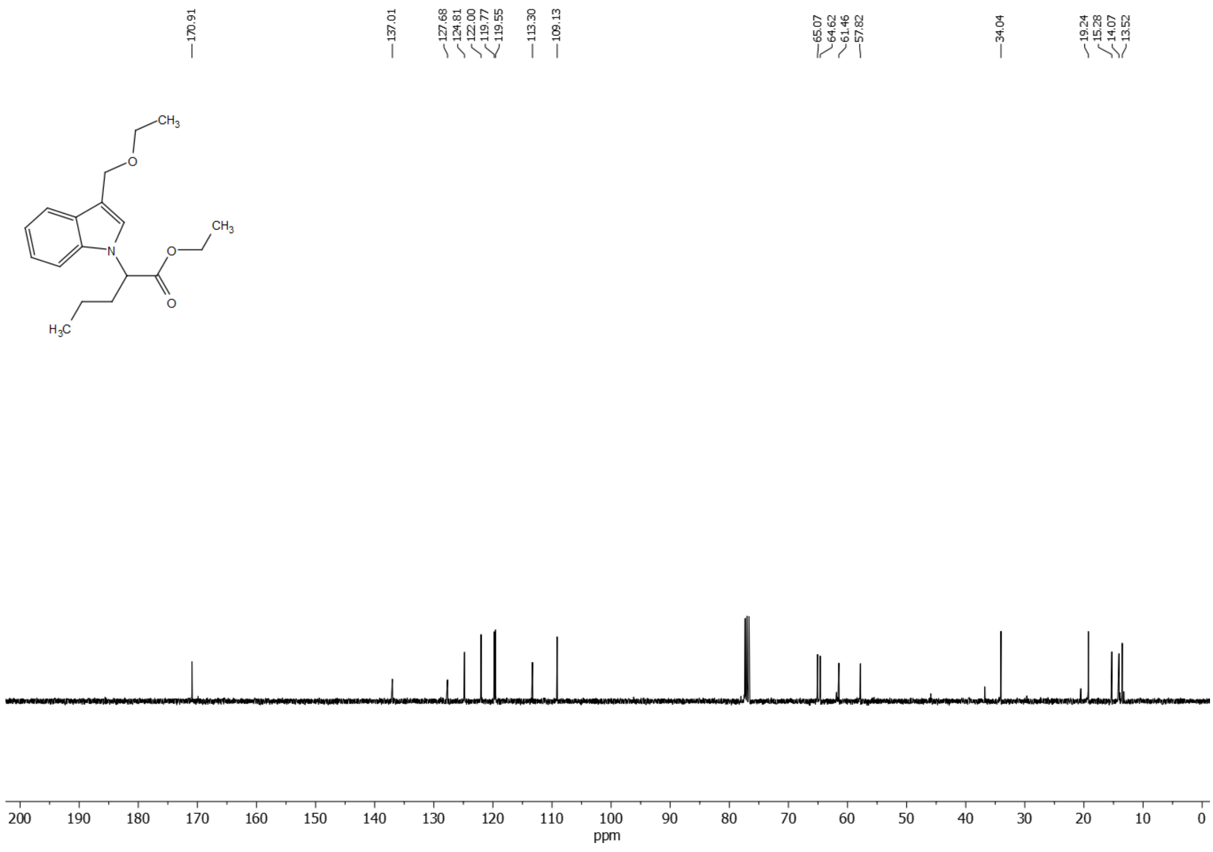

Fig.S20b. ^{13}C NMR spectrum of compound **23**

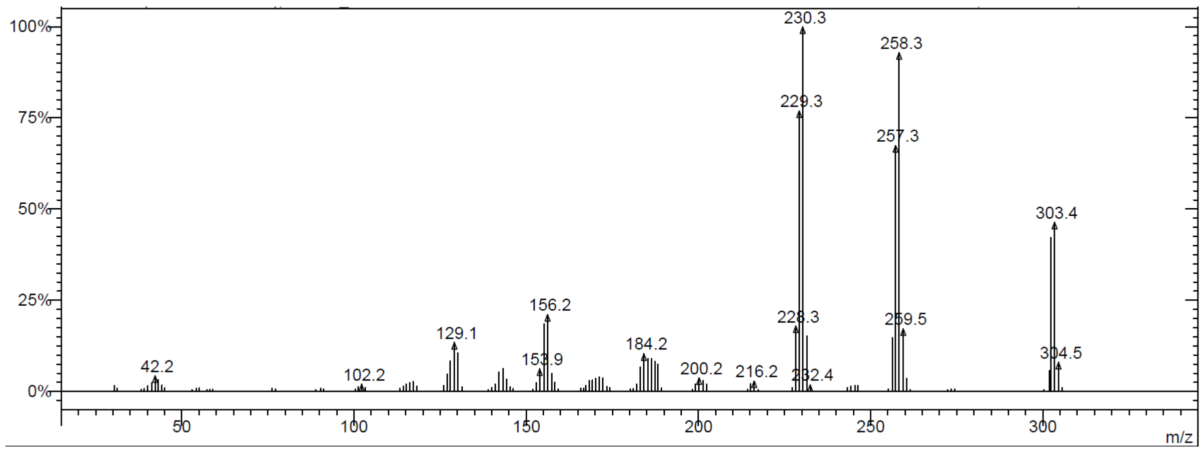

Fig.S20c. EI-MS spectrum of compound **23**

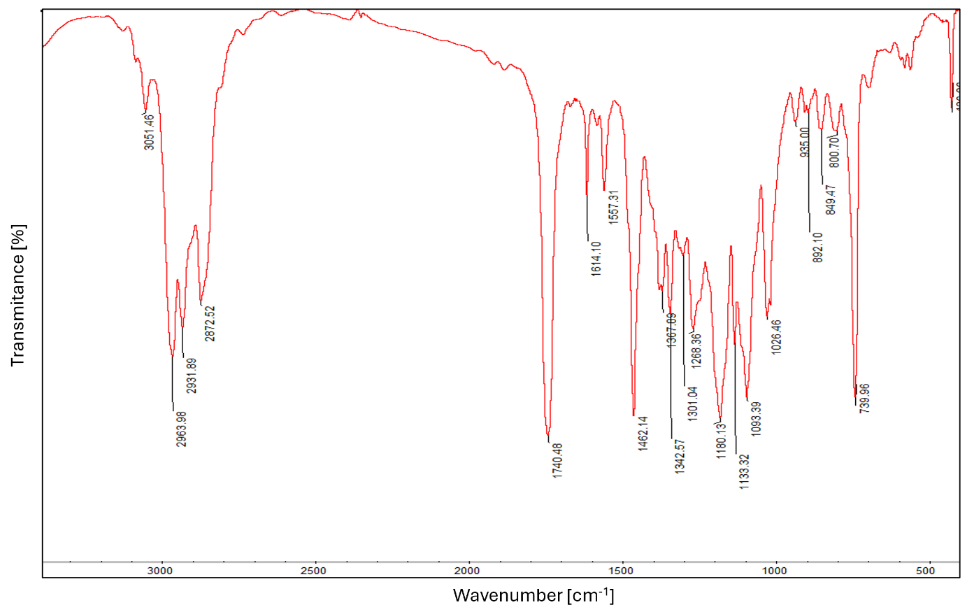

Fig.S20d. FT-IR spectrum of compound **23**

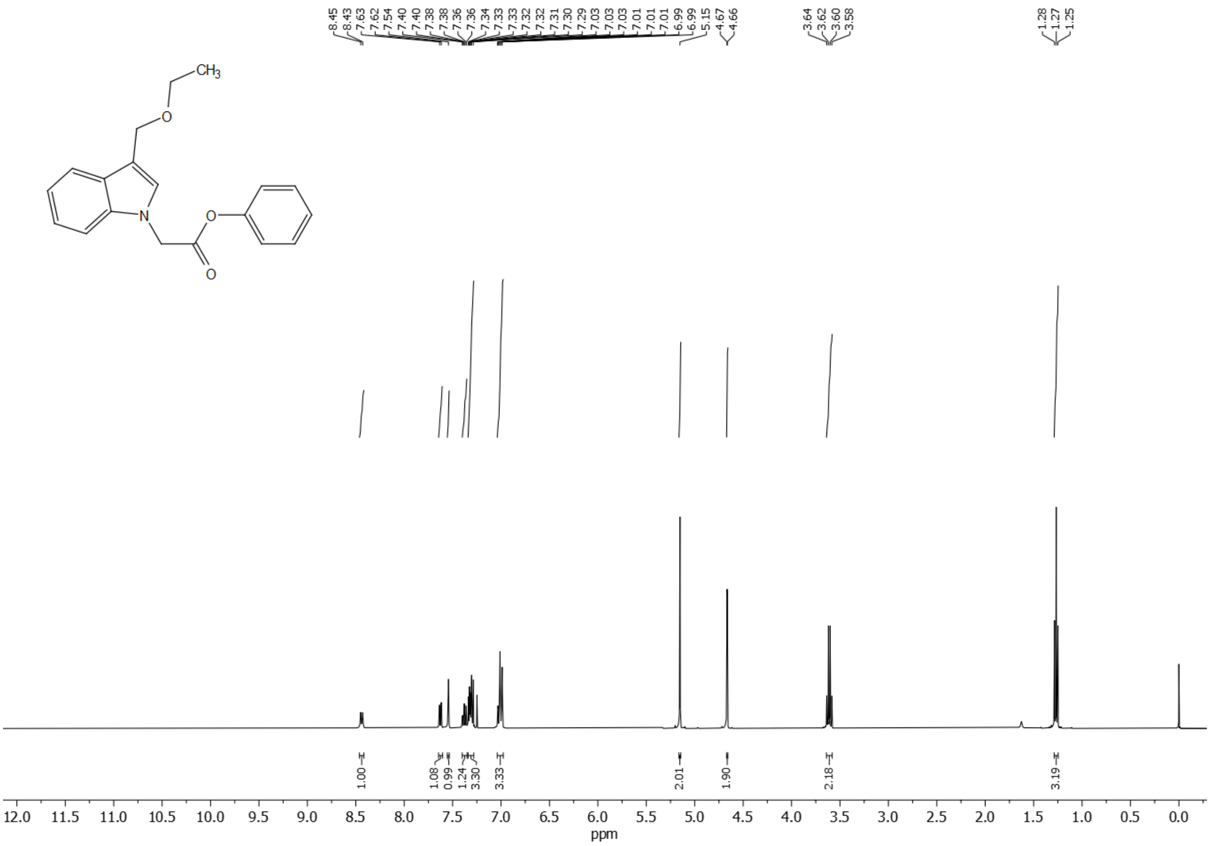

Fig.S21a. ^{1}H NMR spectrum of compound **24**

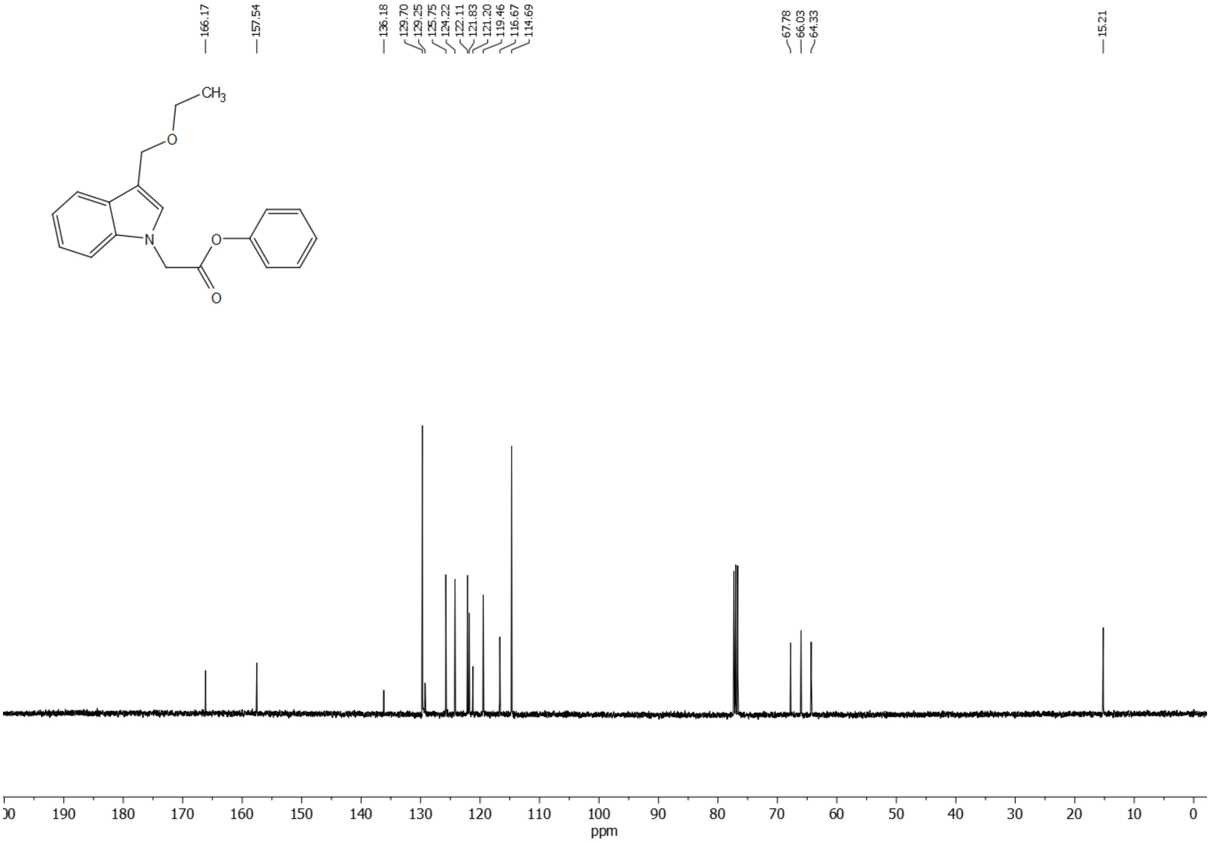

Fig.S21b. ^{13}C NMR spectrum of compound **24**

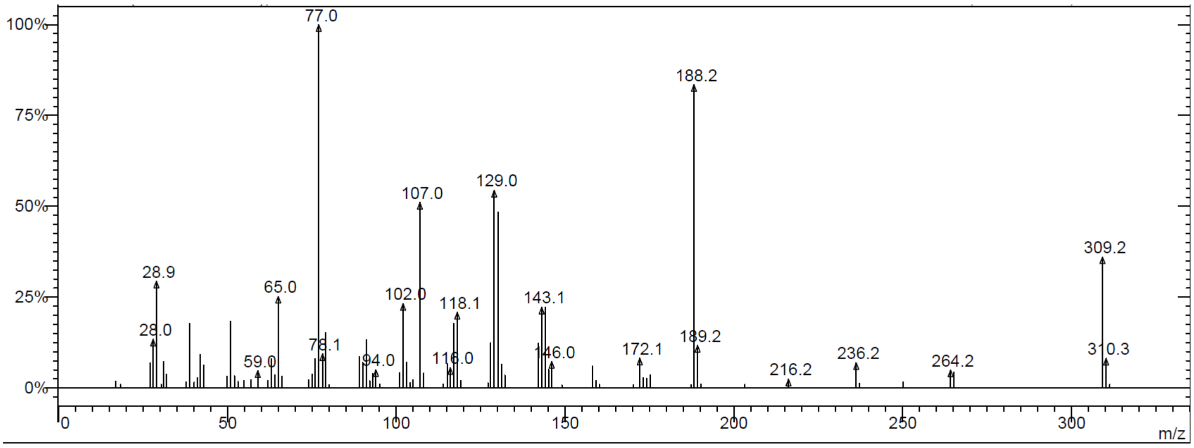

Fig.S21c. EI-MS spectrum of compound **24**

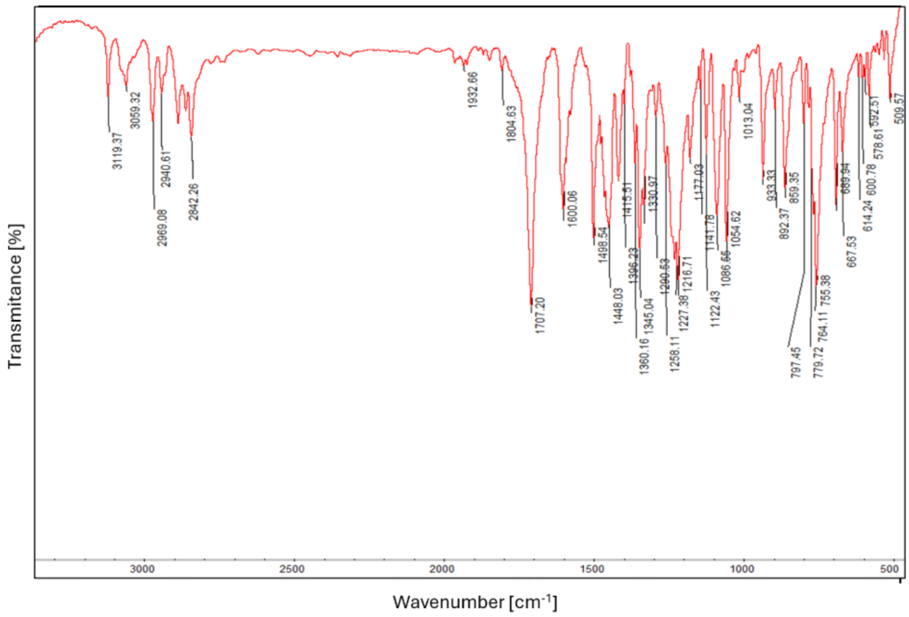

Fig.S21d. FT-IR spectrum of compound **24**

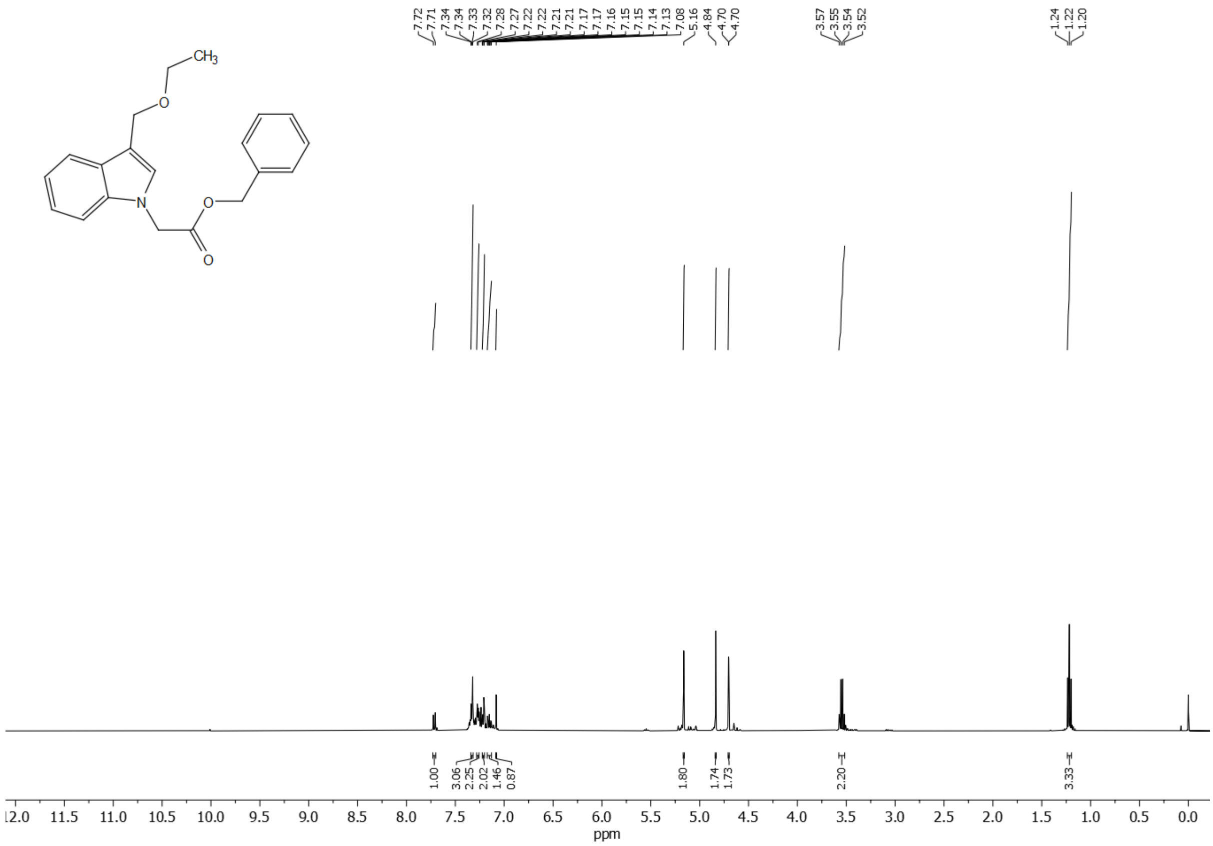

Fig.S22a. ^{1}H NMR spectrum of compound **25**

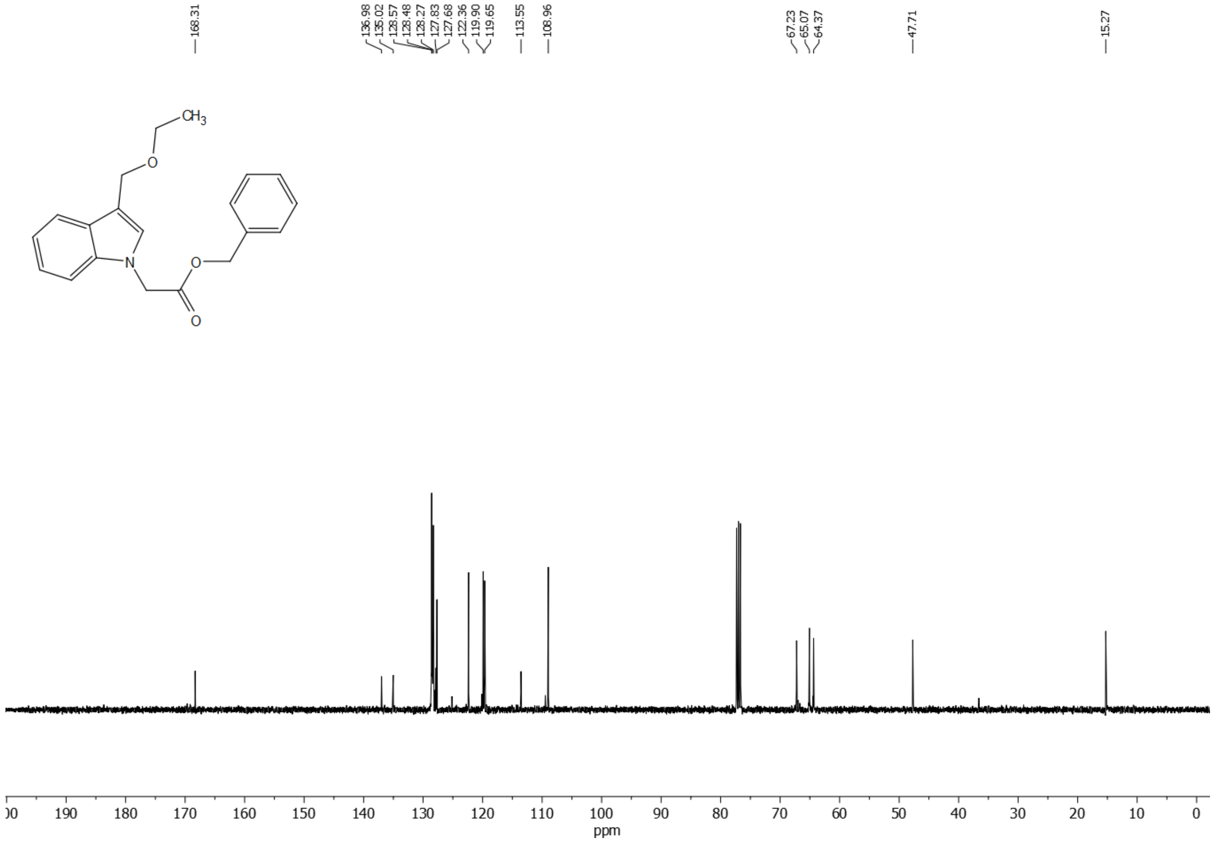

Fig.S22b. ^{13}C NMR spectrum of compound **25**

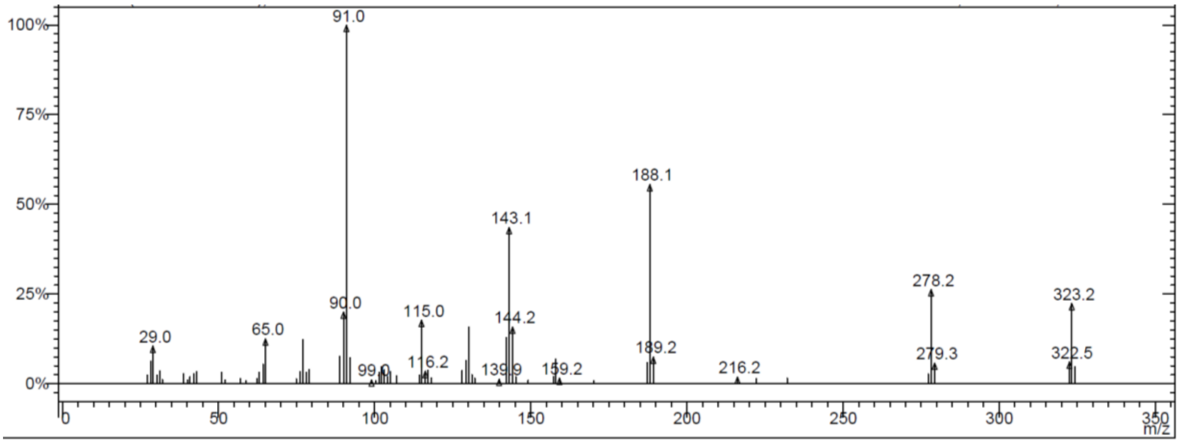

Fig.S22c. EI-MS spectrum of compound **25**

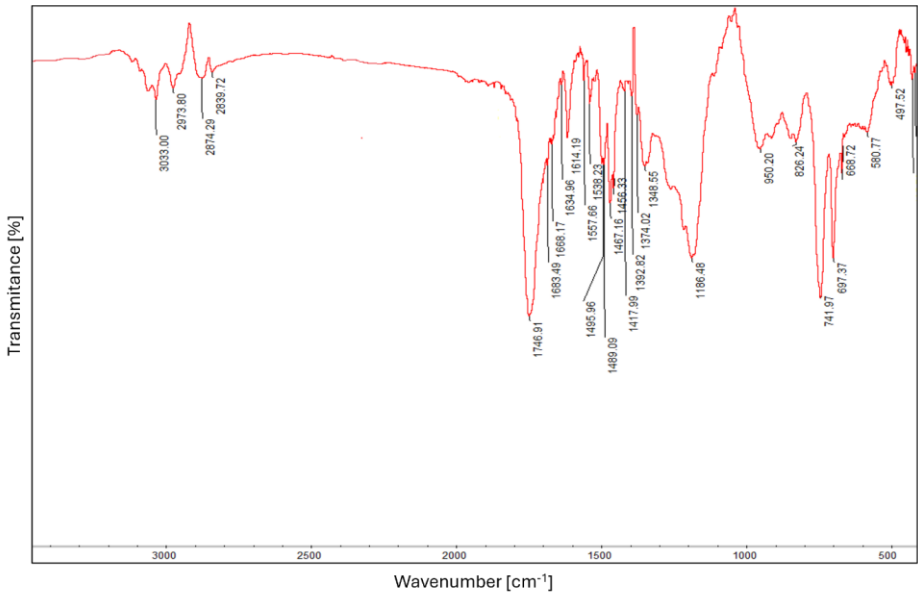

Fig.S22d. FT-IR spectrum of compound **25**

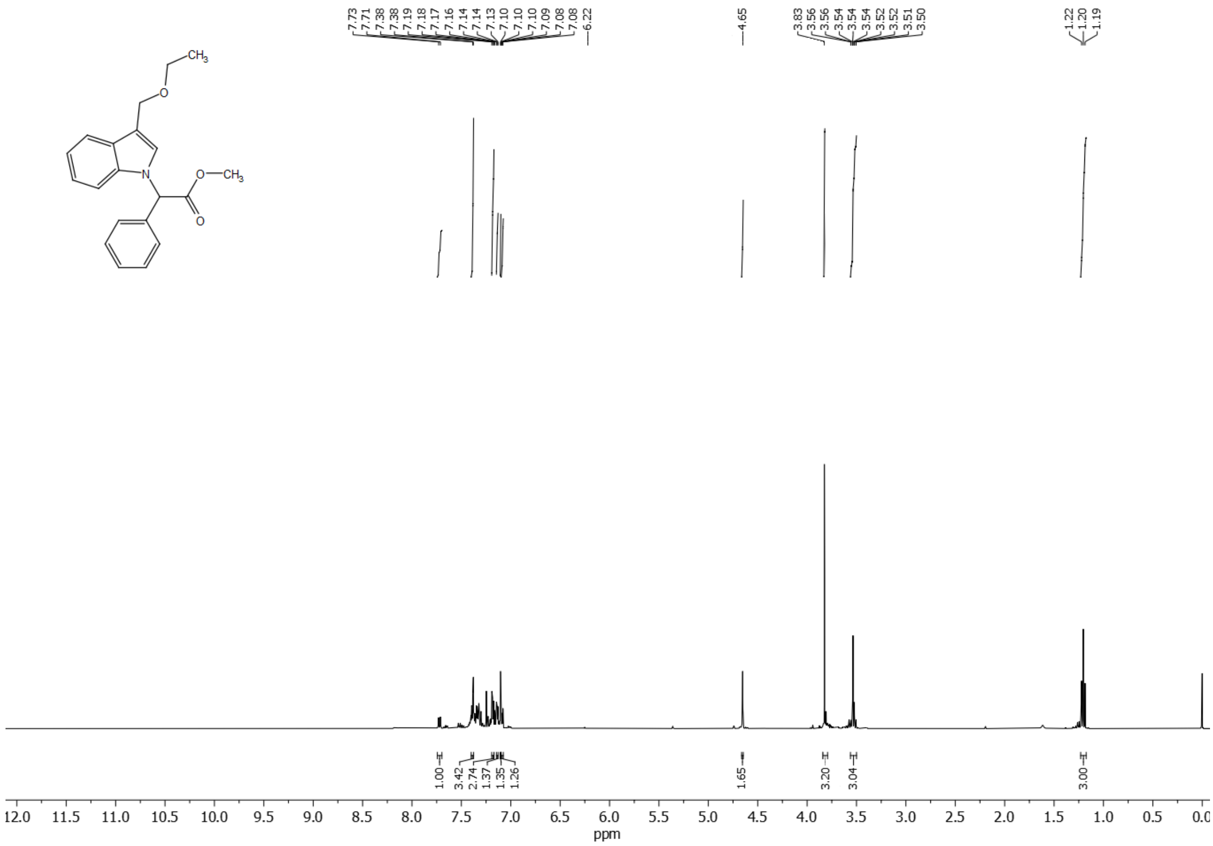

Fig.S23a. ^{1}H NMR spectrum of compound **26**

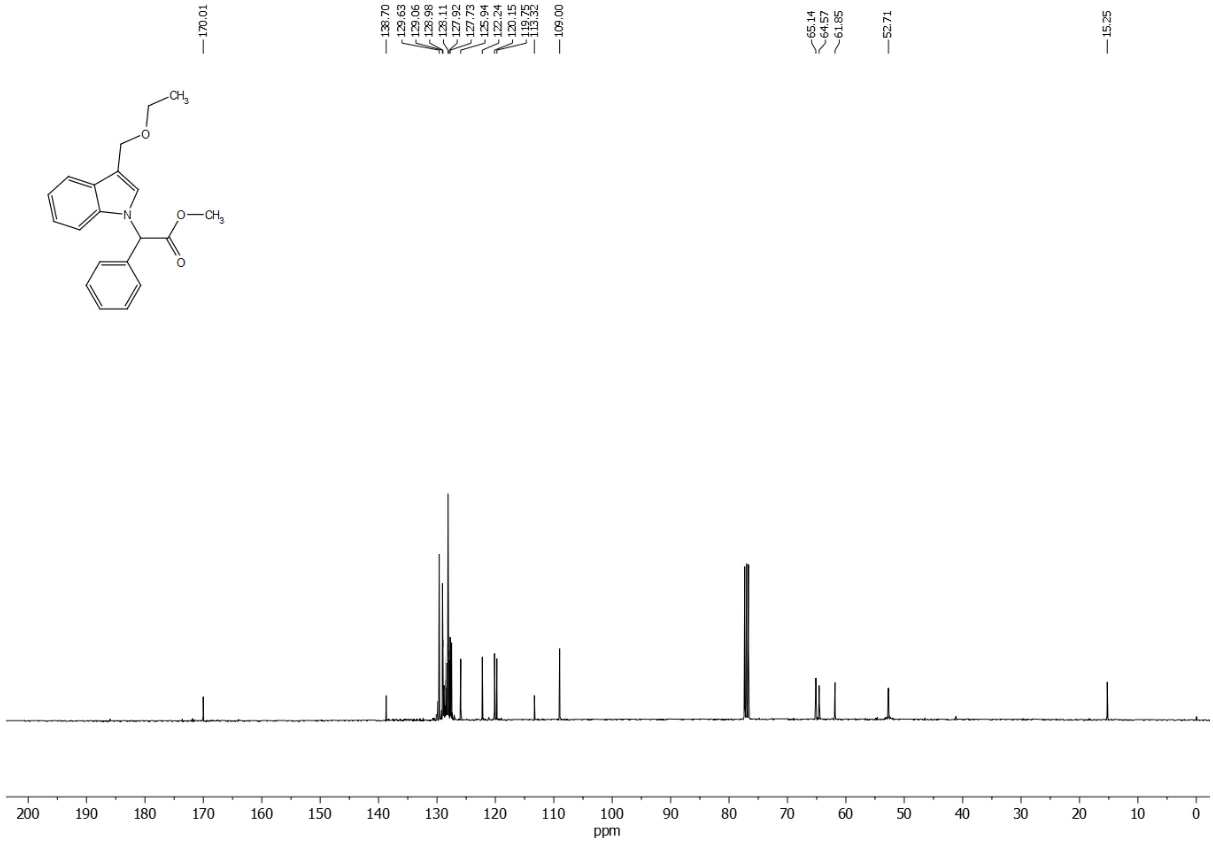

Fig.S23b. ^{13}C NMR spectrum of compound **26**

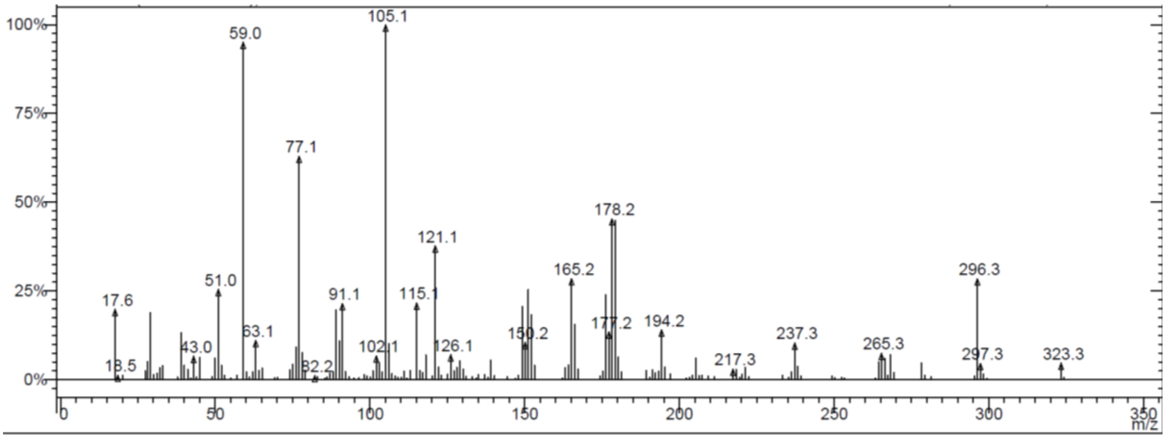

Fig.S23c. EI-MS spectrum of compound **26**

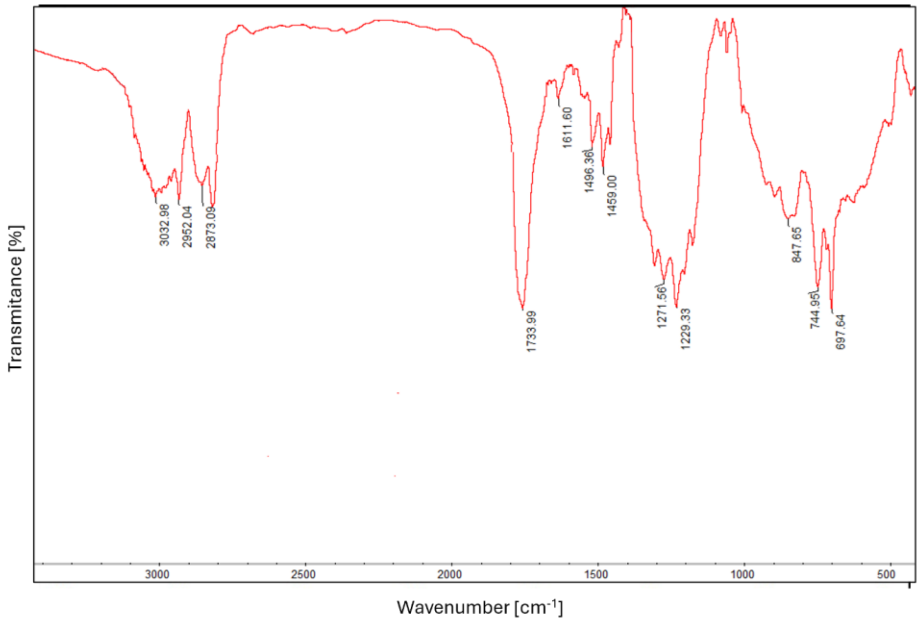

Fig.S23d. FT-IR spectrum of compound **26**

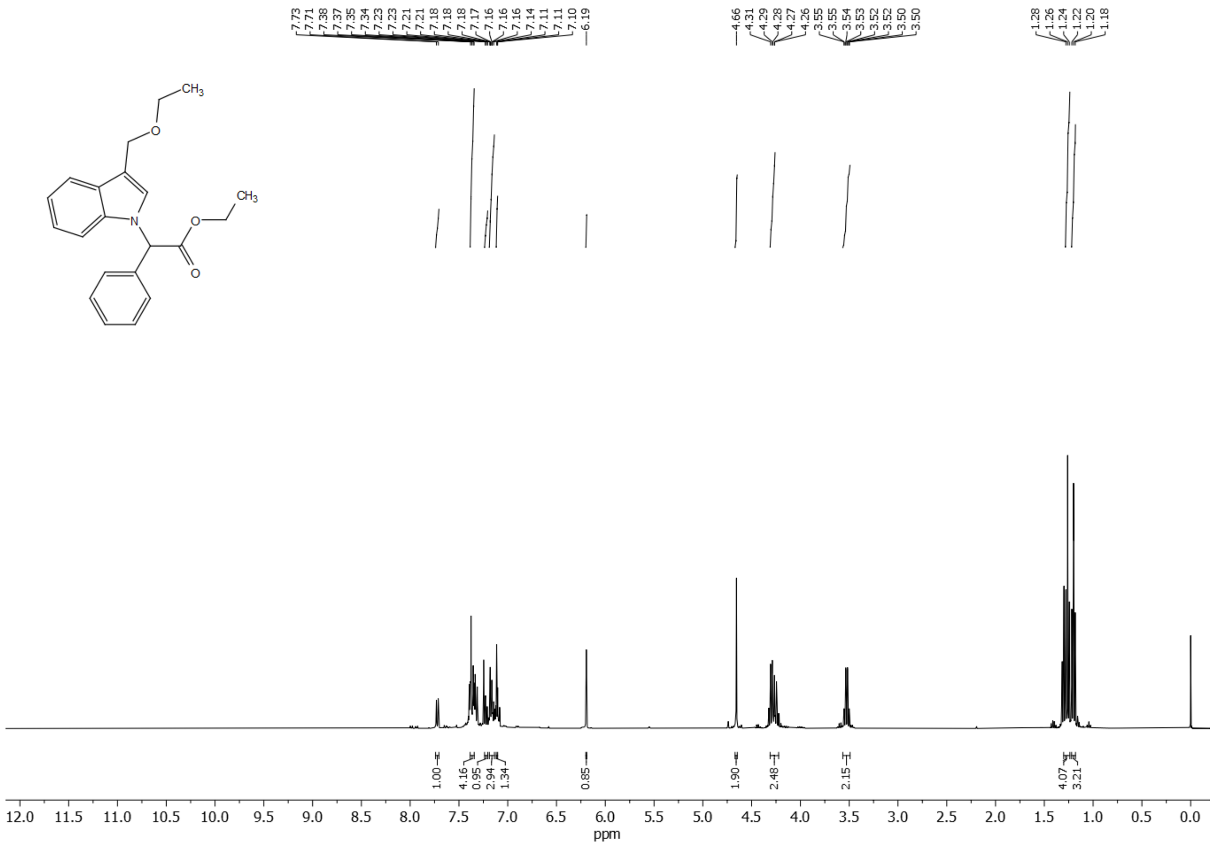

Fig.S24a. ^{1}H NMR spectrum of compound **27**

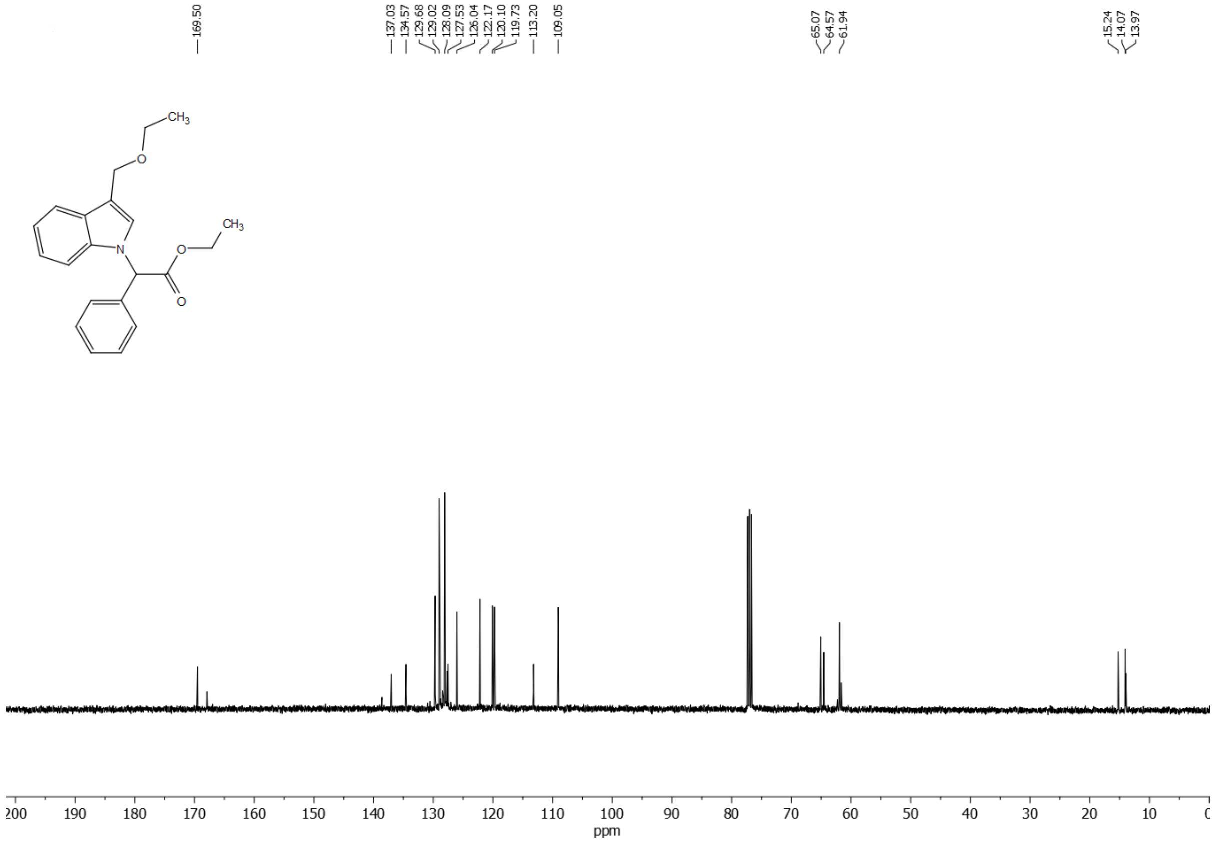

Fig.S24b. ^{13}C NMR spectrum of compound **27**

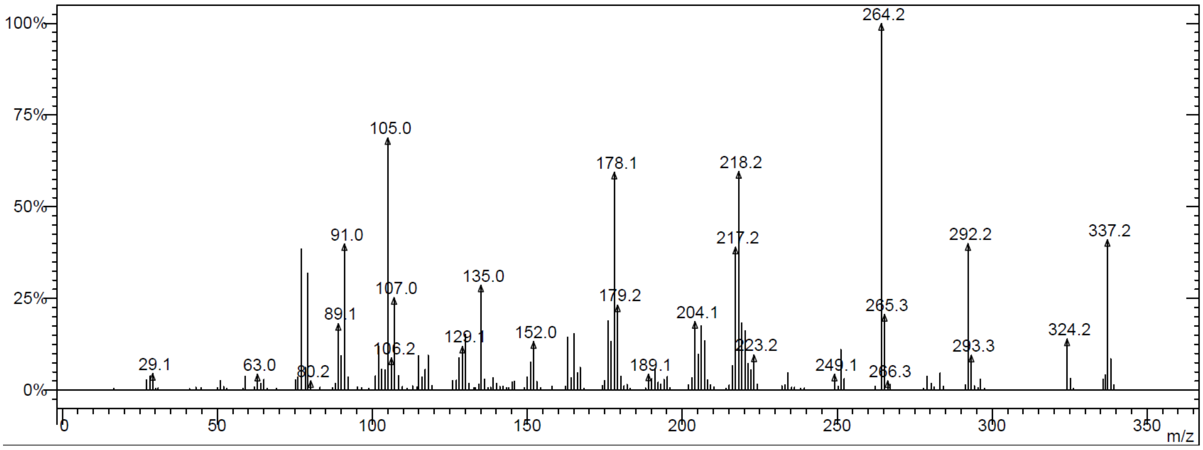

Fig.S24c. EI-MS spectrum of compound **27**

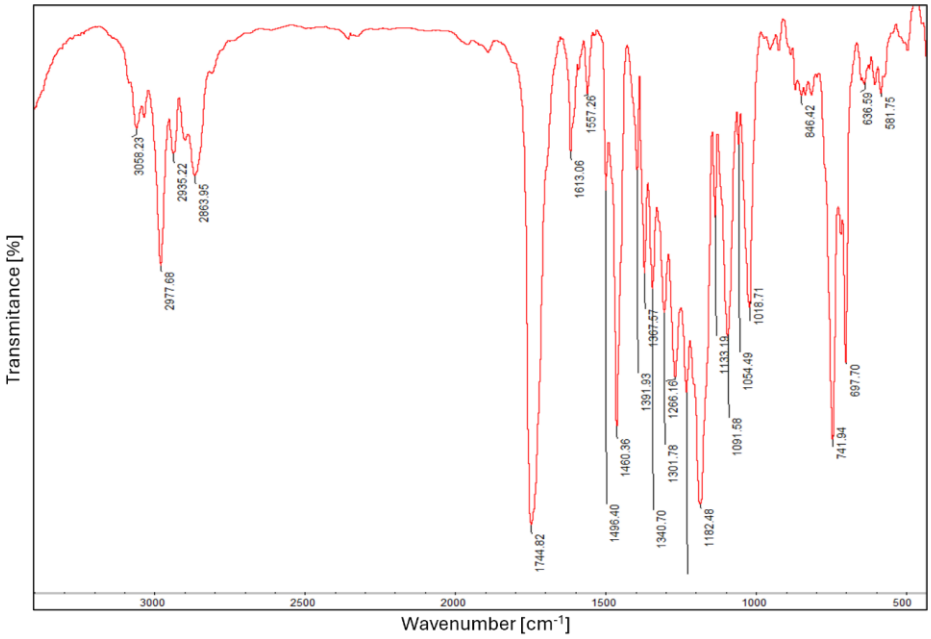

Fig.S24d. FT-IR spectrum of compound **27**

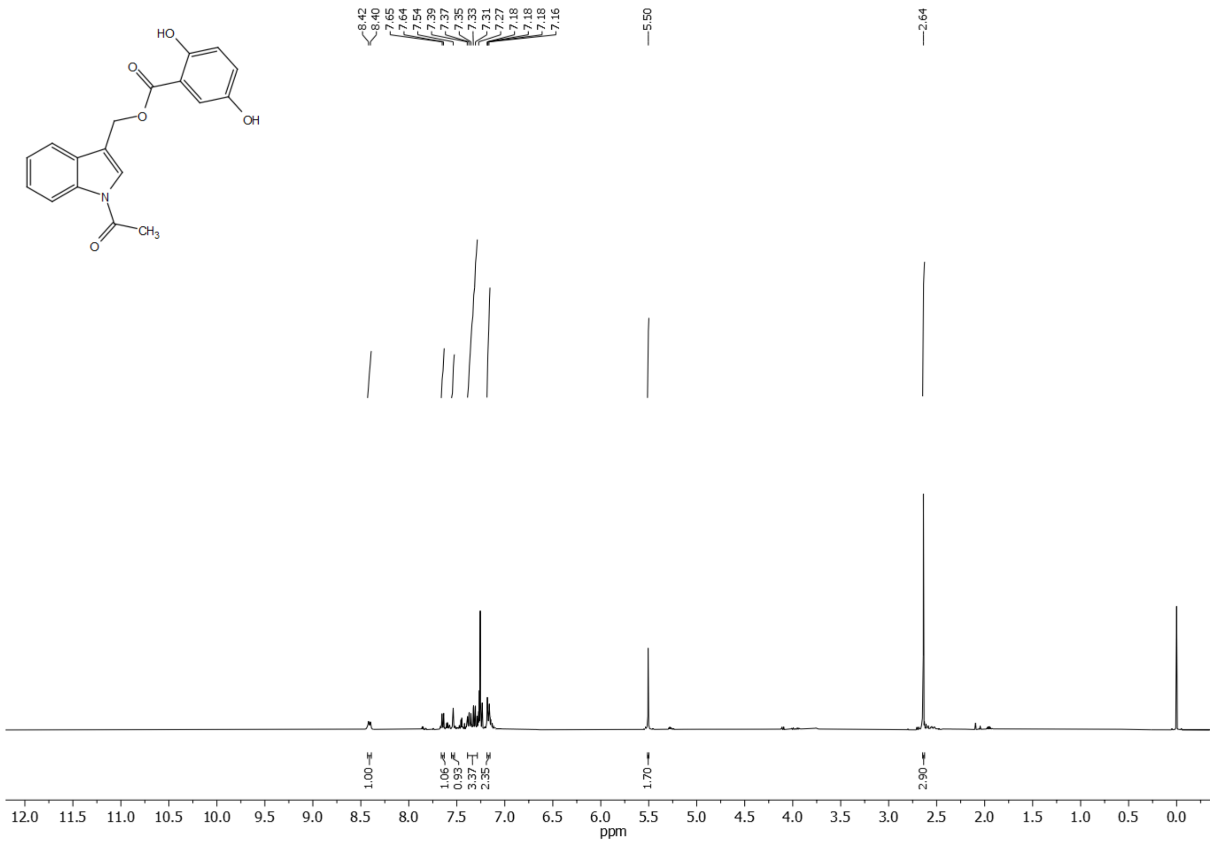

Fig.S25a. ^{1}H NMR spectrum of compound **29**

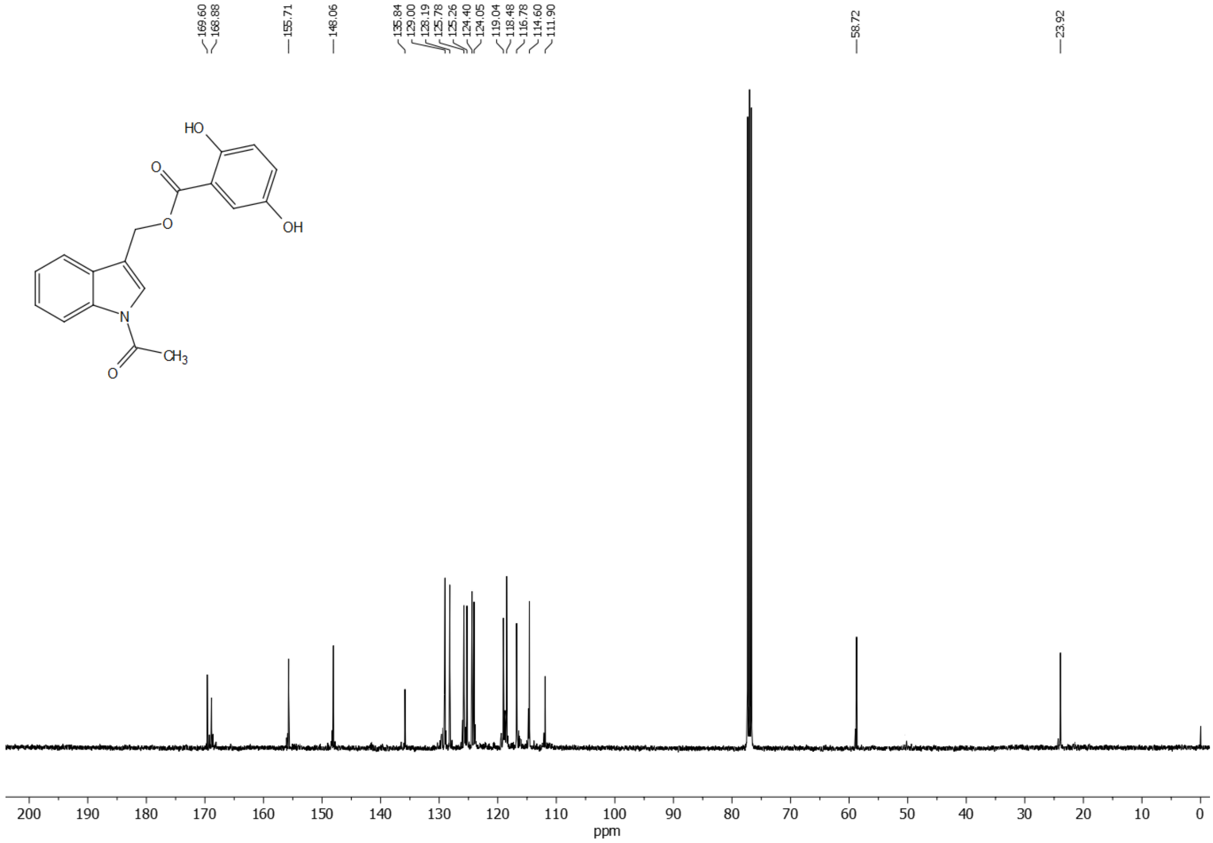

Fig.S25b. ^{13}C NMR spectrum of compound **29**

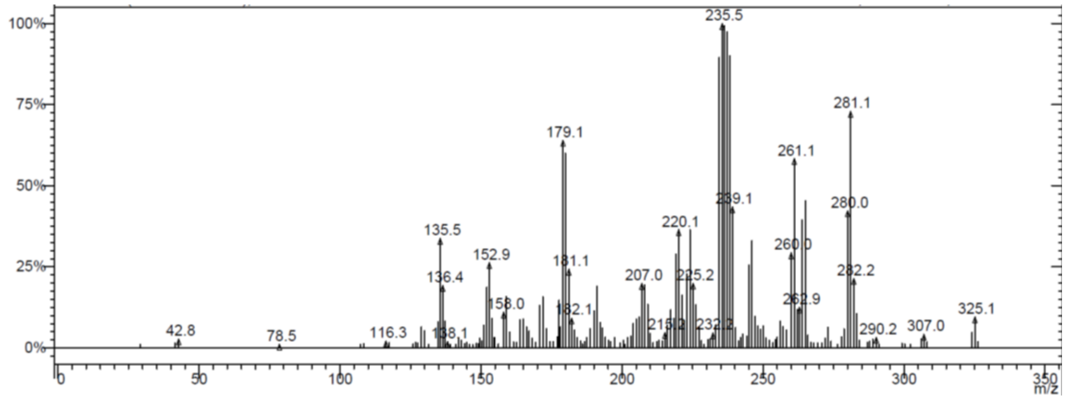

Fig.S25c. EI-MS spectrum of compound **29**

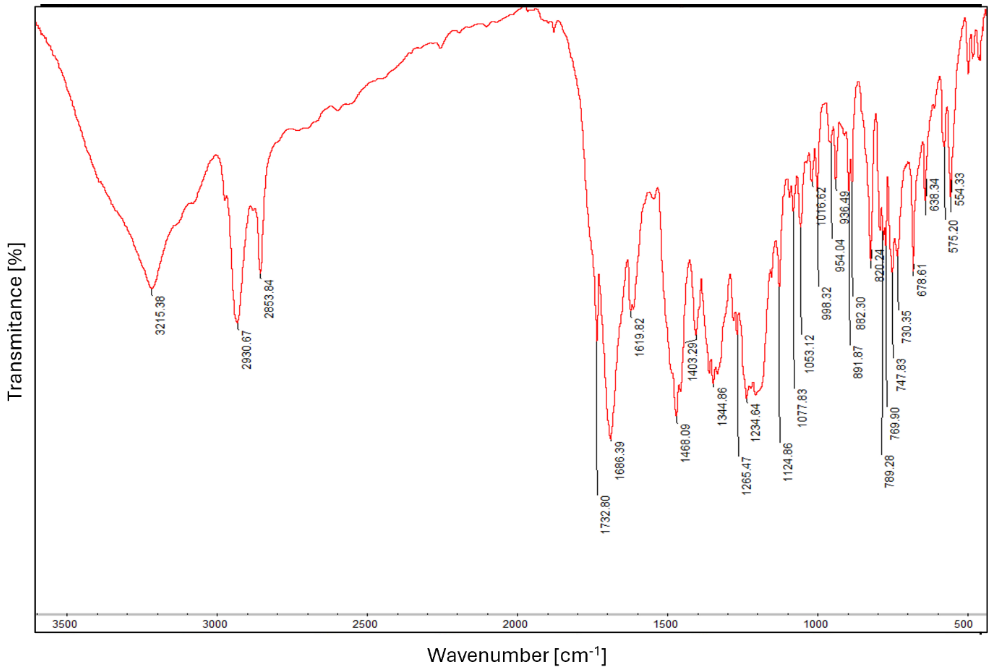

Fig.S25d. FT-IR spectrum of compound **29**

Crystal data Description of the crystallographic results

With only one real exception of molecule **10**, the molecules **2**, **4**, **5**, **7**, **8**, **9** and **11** display in crystals a similar conformation. Chemical alterations such as hydrogenation or annelation with benzene ring have more significant effect on the structure of thiazole than imidazole derivatives on both the molecular and supramolecular levels. Based on the collected structural data we may follow structural changes caused by i) hydrogenation, (ii) methyl substitution, (iii) annelation with benzene ring and (iv) isosteric substitution.

The effect of N-H/N-CH_3 substitution can be tackled by comparing the structures **4** and **5**. As can be seen from Table 1, the molecular conformations are nearly identical but, as could be expected, the number of intermolecular hydrogen bonds has been reduced upon *N*-methylation (Table S1). Analogously, a consequence of annelation with benzene ring can be chased by comparing molecules **5** and **8**. While the lengths of the bonds in the condensed imidazole ring are greater than in the free imidazole system, the two molecules adopt similar conformation in crystals. Both utilize N-H_{indole}···S hydrogen bond to form chains of molecules related either by a glide plane (**5**) or a two-fold screw axis (**8**). The arrangement of the latter molecules around a two-fold screw axis allows to overcome the steric hindrance by directing outwards the bulky methyl substituents and benzene rings (Figure 7). As a result, the hydrogen bond in **8** is the least linear of all the reported N-H···S hydrogen bonds (Table S1).

Although hydrogenation of imidazole (**4**) into imidazolidine (**2**) changes the bond lengths, diminishes the propeller feature of **4** (Table 1), and modifies the overall hydrogen-bonding pattern, in both crystals the molecules utilize the same set of two N-H and one C-H donor groups to the thione acceptor to connect molecules into three dimensions. The H-bond parameters that are listed in Table S1 are more adequate for **4** than **2**. Sulphur atom acts as a quadruple acceptor, again illustrating its capacity to simultaneously engage in a greater number of interactions than conventional acceptors. The C=S bond in molecules **2** and **4** amounts to 1.682(4) and 1.697(2) Å, respectively, which seems to indicate that the thione tautomer in **4** has more significant contribution of the zwitterionic structures that involve single C^+–S^- covalent bond (Figure 3B). While searching for possible explanation of this phenomenon we have noticed the existence of a short S···S contact (3.5694(8) Å) in **4** suggestive of the presence of S···S interactions. The two C=S···S angles are equal in value and amount to 171.56(5)° and the C=S···S=C torsion angle measures -142.0(7)°. Based on these geometrical parameters we were tempted to assume that the C=S bond in **4** elongates as

a result of the formation of the of chalcogen bond. However, the attractive character of such interaction has been questioned by Owczarzak *et al*. on the bases of experimental charge density studies and theoretical calculations carried out for selected thioamides [S1]. The authors postulate that the short S···S contacts in thioamides originate from the presence of the so called “staple molecules”. By forming strong hydrogen bonds, these molecules bring two sulphur atoms into close contact, which by nature is rather repulsive than attractive. In our case, the close S···S contact is reinforced by the imidazole H-N-C-H fragments, acting as “staples” (Figure S26). The stapling imidazole moieties can stabilize the zwitterionic mesomers in **4**, which in turn can account for the elongation of the C=S bond. The imidazolidine moiety in the crystals of **2** does not display such stapling capability.

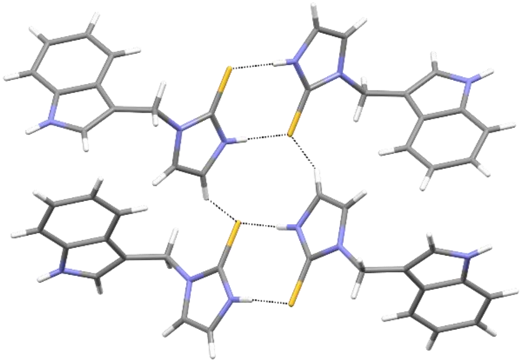

Fig.S26. Hydrogen-bonded tape motif present crystals of **4**. Resonance-assisted N-H···S hydrogen-bond dimers (formed around symmetry centres) are fused with a tetramer formed around two-fold symmetry axis, in which the sulphur atoms are stapled by two H-C-N-H imidazole fragments.

Like molecules **2** and **4**, the molecule **7** also contains two N-H hydrogen bond donor groups (although both come from the indole moieties) and a thione acceptor. However, most likely due to the steric hindrance, the molecules of **7** incorporate to their crystal lattice ethanol and water solvent molecules. The system becomes particularly rich in hydrogen bond functionalities thus leading to the formation of a whole palette of hydrogen bonds. All types of molecules utilize fully their potential in hydrogen bonding. Particular attention should be given to the formation of O-H(water)···π(pyrrole) hydrogen bonds (Table S1). The role of solvent molecules in stabilizing the crystal structure of **7** is shown in Figure 6, which also illustrates the involvement of molecules **7** in weak π···π interactions between centrosymmetrically related benzene rings from benzothiazole fragments. The distance between centroids of the two fragments in a stack amounts to 3.925 Å, while the distance between their planes is 3.409 Å (symmetry code –x, 1 – y, 1 –z). The planes are parallel and

the displacement of the rings in a stack is 1.945 Å. Due to the presence of supporting guest molecules, such extended hydrogen bond pattern does not significantly affect the molecular conformation, which retains a propeller shape.

Crystal data presented in Table S2 reveal that **5** and **9** are isostructural despite the fact that **N**-methyl imidazole has been substituted by thiazolidine. This could indicate that the replacement of the N-CH_3 group by a sulphur atom and hydrogenation of the five-membered ring are insignificant as far as molecular conformation and supramolecular assemblies are concerned, supposedly because neither of these two fragments is involved in hydrogen bonding. Changes in molecular conformation of **11** as compared with its parent compound (non-methylated at C5) [16] are not substantial but again might be ascribed to different interactions in crystals caused by steric hindrance. Methyl substitution in **11** prevents formation of columnar stacking interactions between benzoxazole fragments, operating in the parent compound. Instead, we observe strong competition between S and O atoms for indole N-H donor and the resulting formation of a three centre hydrogen bond to both acceptors (Table S1, Figure S27). The N···S(=C) distance is the longest in the reported structures. With respect to molecular conformation, the consequences of the S/O replacement are not straightforward. The oxazole derivative differs in conformation from both thiazole and benzothiazole derivatives but resembles the hydrogenated thiazole derivative **9** (Table 1).

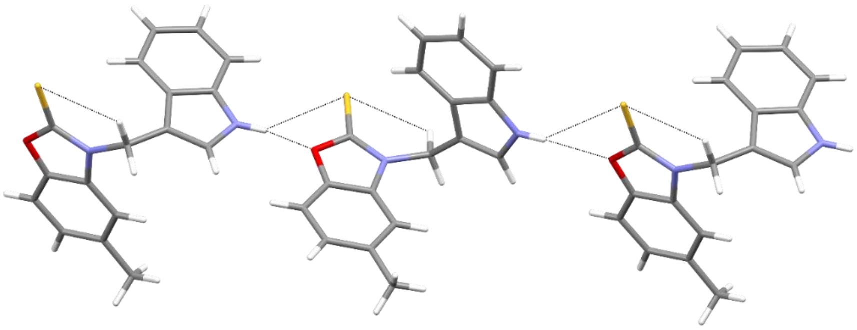

Fig.S27. Competition between S and O atoms for indole N-H donor and the resulting association of molecules 11 into chains by means of a three centre hydrogen bond to both acceptors.

References

[S1] Owczarzak, A.; Dutkiewicz, Z,; Kurczab, R.; Pietruś, W.; Kubicki, M.; Grześkiewicz, A.M. Role of Staple Molecules in the Formation of S···S Contact in Thioamides: Experimental Charge Density and Theoretical Studies. *Crystal Growth & Design* **2019,** *19*, 7324-7335, doi: 10.1021/acs.cgd.9b01204.

Table S1. Hydrogen bond geometrical parameters. Intramolecular interactions are written in italics.

D—H···*A*	*D*—H (Å)	H···*A* (Å)	*D*···*A* (Å)	*D*—H···*A* (°)
**2**				
N1—H1···S1^{i}	0.86	2.65	3.445 (4)	155.1
N3—H3···S1^{ii}	0.86	2.57	3.345 (3)	150.5
C13—H13A···S1^{iii}	0.97	2.87	3.596 (5)	132.3
C10—H10B···S1	*0.97*	*2.78*	*3.155 (4)*	*103.6*
**4**				
N1—H1···S1^{iv}	0.86	2.50	3.3549 (16)	170.4
N3—H3···S1^{v}	0.86	2.52	3.3613 (15)	164.6
C13—H13···S1^{vi}	0.93	2.81	3.562 (2)	138.3
C10—H10B···S1	*0.97*	*2.77*	*3.2500 (17)*	*111.2*
**5**				
N1—H1···S1^{vii}	0.86	2.58	3.390 (2)	157.6
C10—H10B···S1	*0.97*	*2.81*	*3.275 (3)*	*110.6*
**7**				
N1—H1···O1W^{v}	0.86	2.31	2.996 (3)	136.8
N11—H11···O1W	0.86	2.13	2.924 (3)	153.3
O1W—H1WA···O1ET	0.85	1.83	2.647 (3)	160.8
O1ET—H1ET···S1^{viii}	0.82	2.49	3.305 (3)	171.8
O1W—H1WB … $Cg_{(pyrrole)}$	0.85	2.48	3.280	157.9
C10—H10A···S1	*0.97*	*2.73*	*3.229 (2)*	*112.4*
C20—H20A···S1	*0.97*	*2.73*	*3.215 (2)*	*111.6*
**8**				
N1—H1···S1^{ix}	0.86	2.59	3.344 (3)	146.5
C10—H10A···S1	*0.97*	*2.77*	*3.236 (3)*	*110.4*
C4—H4···S1	*0.93*	*2.93*	*3.760 (3)*	*149.0*
C18—H18B···S1	*0.96*	*2.81*	*3.193 (3)*	*104.9*
**9**				

N1—H1···S1^{x}	0.86	2.58	3.4186 (16)	164.7
C10—H10A···S1	*0.97*	*2.72*	*3.2047 (18)*	*111.2*
**10**				
N1—H1···S1^{xi}	0.88	2.55	3.3896 (14)	160.5
C10—H10A···S1^{v}	0.99	2.96	3.6652 (16)	128.8
C12—H12···S1^{viii}	0.95	3.00	3.5090 (16)	114.8
C13—H13···S1^{viii}	0.95	2.95	3.4711 (17)	115.6
C10—H10B···S1	*0.99*	*2.73*	*3.1503 (16)*	*106.2*
**11**				
N1—H1···S1^{xii}	0.88	2.72	3.4702 (13)	143.5
N1—H1···O1^{xii}	0.88	2.61	3.1495 (17)	120.7
C10—H10A···S1	*0.99*	*2.78*	*3.2540 (17)*	*109.7*

Symmetry code(s): (i) -*x*+1, -*y*, -*z*+1; (ii) -*x*, -*y*, -*z*+1; (iii) *x*, -*y*+1/2, *z*+1/2; (iv) *x*+1/2, -*y*+1/2, -*z*+1; (v) -*x*+1, -*y*+1, -z+1; (vi) *x*, -*y*+1, *z*-1/2; (vii) *x*+1/2, -*y*+3/2, *z*+1/2; (viii) *x*, *y*+1, *z*; (ix) -*x*+1/2, -*y*+1, *z*+1/2; (x) *x*+1/2, -*y*+1/2, *z*+1/2; (xi) -*x*+1/2, *y*+1/2, -*z*+1/2; (xii) *x*+1, -*y*+3/2, *z*+1/2

Table S2. Crystal data and structure refinement parameters for selected gramine derivatives.

	**2**	**4**	**5**	**7**	**8**	**9**	**10**	**11**
**Crystal data**								
Chemical formula	$C_{12}H_{13}N_3S$	$C_{12}H_{11}N_3S$	$C_{13}H_{13}N_3S$	$C_{25}H_{20}N_4S\cdot C_2H_6O\cdot H_2O$	$C_{17}H_{15}N_3S$	$C_{12}H_{12}N_2S_2$	$C_{12}H_{10}N_2S_2$	$C_{17}H_{14}N_2OS$
M_r	231.31	229.30	243.32	472.59	293.38	248.36	246.34	294.36
Crystal system space group	Monoclinic $P2_1/c$	Orthorhombic *Pbcn*	Monoclinic $P2_1/n$	Triclinic $P\bar{1}$	Orthorhombic $P2_12_12_1$	Monoclinic $P2_1/n$	Monoclinic $P2_1/n$	Monoclinic $P2_1/c$
Temperature (K)	295	293	293	293	293	293	100	130
a (Å)<br>*b* (Å)<br>*c* (Å)	10.4517 (8)<br>8.4232 (6)<br>13.5631 (11)	18.4010 (3)<br>11.2626 (3)<br>11.2577 (2)	9.1819 (3)<br>14.2705 (3)<br>9.5585 (3)	7.4924 (3)<br>13.1185 (5)<br>13.4710 (6)	7.1911 (2)<br>13.9158 (4)<br>14.6650 (4)	9.2002 (1)<br>13.4917 (2)<br>9.6702 (1)	7.9077 (2)<br>7.2185 (2)<br>19.4666 (5)	7.42703 (16)<br>14.3167 (3)<br>13.8146 (3)
α (°)<br>β (°)<br>γ (°)	90<br>102.934 (8)<br>90	90<br>90<br>90	90<br>92.203 (3)<br>90	114.605 (4)<br>96.556 (4)<br>92.830 (3)	90<br>90<br>90	90<br>93.384 (1)<br>90	90<br>97.084 (3)<br>90	90<br>102.016 (2)<br>90
V ($Å^3$)	1163.75 (16)	2333.08 (8)	1251.53 (6)	1189.05 (9)	1467.53 (7)	1198.23 (3)	1102.70 (5)	1436.73 (5)
Z	4	8	4	2	4	4	4	4
D_x (Mg m^{-3})	1.320	1.306	1.291	1.320	1.328	1.377	1.484	1.361
Radiation type	Mo *K*α	Cu *K*α	Cu *K*α	Cu *K*α	Cu *K*α	Cu *K*α	Mo *K*α	Cu *K*α
μ (mm^{-1})	0.25	2.26	2.13	1.47	1.92	3.80	0.45	1.99
Crystal size (mm)	0.50 × 0.45 × 0.01	0.30 × 0.20 × 0.03	0.30 × 0.15 × 0.10	0.50 × 0.05 × 0.02	0.40 × 0.15 × 0.03	0.65 × 0.15 × 0.05	0.5 × 0.4 × 0.2	0.18 × 0.15 × 0.01
**Data collection**								
T_{min}, T_{max}	0.366, 1.000	0.595, 1.000	0.796, 1.000	0.783, 1.000	0.906, 1.000	0.335, 1.000	0.982, 1.000	0.789, 1.000
No. of measured, independent and observed [$I > 2\sigma(I)$] reflections	6864, 2008, 1047	19161, 2447, 2087	10993, 2598, 2281	19352, 4867, 4010	5861, 2993, 2724	49696, 2523, 2255	6089, 2302, 2099	5876, 2842, 2444
R_{int}	0.069	0.035	0.024	0.027	0.027	0.052	0.017	0.020
$(\sin \theta/\lambda)_{max}$ ($Å^{-1}$)	0.592	0.631	0.630	0.630	0.630	0.631	0.666	0.625
**Refinement**								
$R[F^2 > 2\sigma(F^2)]$<br>$wR(F^2)$<br>*S*	0.065<br>0.145<br>1.04	0.042<br>0.127<br>1.05	0.059<br>0.164<br>1.08	0.051<br>0.159<br>1.06	0.039<br>0.102<br>1.07	0.038<br>0.113<br>1.08	0.029<br>0.070<br>1.03	0.037<br>0.099<br>1.06

Table S3. Antibacterial activities of compounds **1-29**

Compound	Zone of growth inhibition [mm]			
	Micrococus luteus	*Bacillus subtilis*	*Escherichia coli*	*Pseudomonas fluorescens*
**Gramine (1)**	0	0	0	0
**2**	3.3	4.5	2	4
**3**	1	1	1	1
**4**	1	2	0	1.5
**5**	3.8	5	2	4
**6**	3.5	3.3	1	2
**7**	4	2.3	1	2
**8**	1.8	1	1	1
**9**	1.3	2	1	3
**10**	3	4.2	0	4
**11**	0	0	0	0
**12**	2	3	0	5.5
**13**	7.3	9.4	0	10.5
**15**	11	3.3	7.7	0
**17**	1	1	2	1
**18**	0	0	2.5	0
**19**	0	1	2	1
**20**	1	0	2	0
**21**	3.2	4.5	1	3
**22**	0	1	3	0
**23**	1	0	3	0
**24**	0	0	3.4	0
**25**	0	1	1	1
**26**	0	0	0	0
**27**	1	0	0	0
**29**	1.3	2	1	1

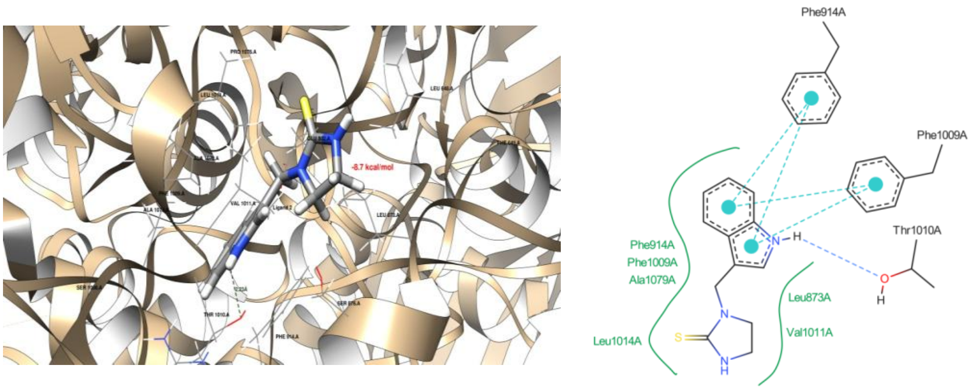

(**a**) (**b**)

Fig. S28. (**a**) The interactions between the derivative **2** and the 1N5X protein domain. The dark-green dashed line indicates potential H-bond formation between one of the domain residues (namely THR 1010 A–2.23 Å) and the hydrogen bonded to one of the nitrogen atoms of the ligand; (**b**) The proposed interactions between the 1N5X protein's domain binding site and derivative **2** suggest the formation of one hydrogen bond (blue dashed line). Green solid lines signify hydrophobic contacts, and aqua dashed lines indicate pi-pi interactions.

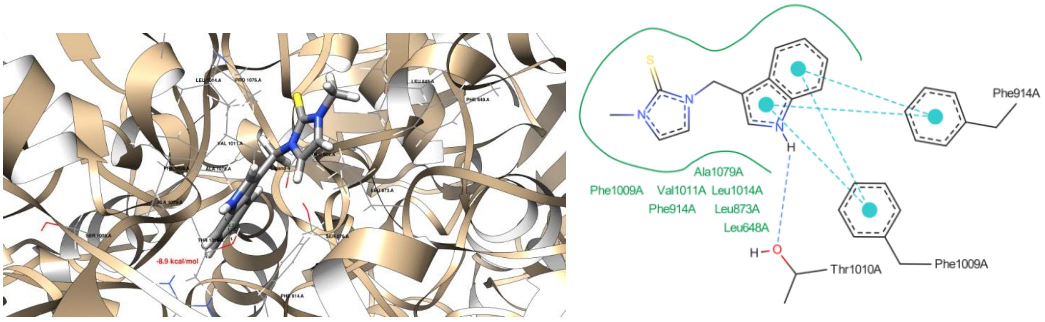

(**a**) (**b**)

Fig. **S29.** (**a**) The interactions between the derivative **5** and the 1N5X protein domain. A dark-green dashed line implies potential hydrogen bond formation between domain residue THR 1010 A, and the ligand’s pyrrolic-hydrogen atom (2.23 Å length); (**b**) The proposed interactions between the 1N5X protein's domain binding site and derivative **5** suggest the formation of one hydrogen bond (blue dashed line). Green solid lines signify hydrophobic contacts, and aqua dashed lines indicate pi-pi interactions.

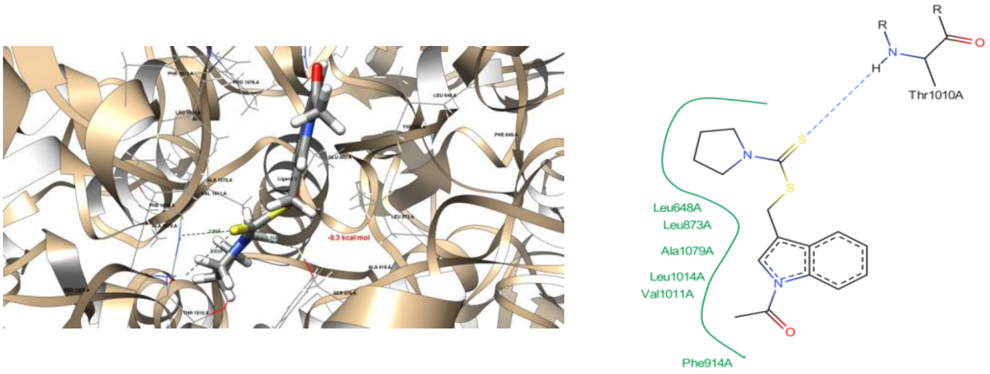

(**a**) (**b**)

Figure S30. (**a**) The interactions between the derivative **15** and the 1N5X protein domain. A dark-green dashed line implies potential hydrogen bond formations between domain residues THR 1010 A (2.83 Å length), VAL 1011 A (2.80 Å length), and SER 876 A residue and the ligand (3.35 Å length); (**b**) The interactions proposed by molecular docking between the 1N5X protein's domain binding site and derivative **15**. According to the ProteinsPlus algorithm, one hydrogen bond is expected, with the possibility of three hydrogen bonds according to the UCSF Chimera software, but this hydrogen bond with THR 1010 A protein residue has the highest probability of formation. Green solid lines indicate hydrophobic contacts.

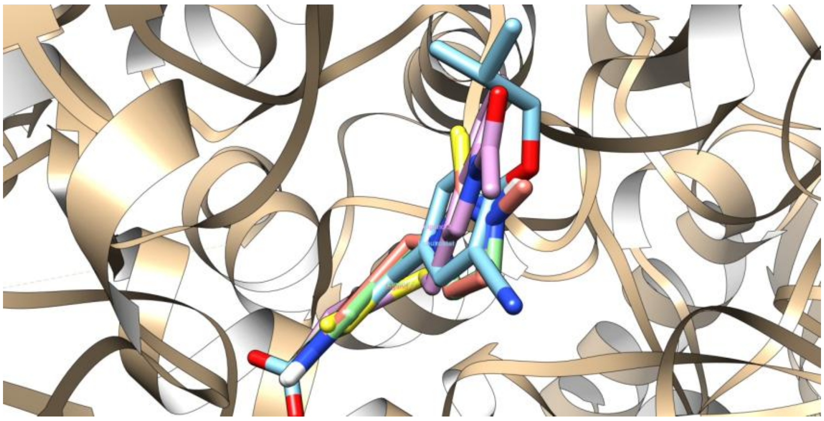

Fig. S31. The native ligand (febuxostat), redocked native ligand, and compounds **2**, **5**, and **15** in the binding site of the 1N5X protein domain.

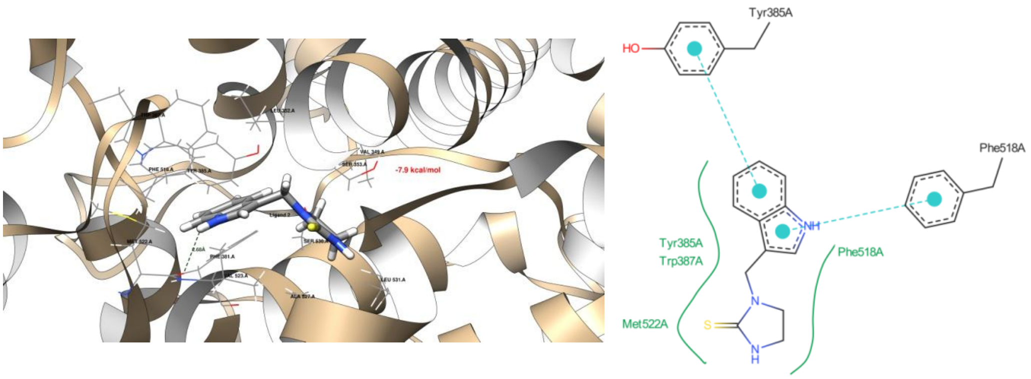

(**a**) (**b**)

Fig. S32. (**a**) The spatial interactions between the derivative **2** and the 4COX protein domain. A dark-green dashed line implies potential hydrogen bond formation between domain residue MET 522 A (2.68 Å length) and the hydrogen bonded to the nitrogen atom of the ligand; (**b**) A closer look at the proposed interactions between the 4COX protein's domain binding site and derivative **2**, with green solid lines indicating hydrophobic contacts and aqua dashed lines indicating cation-pi interaction.

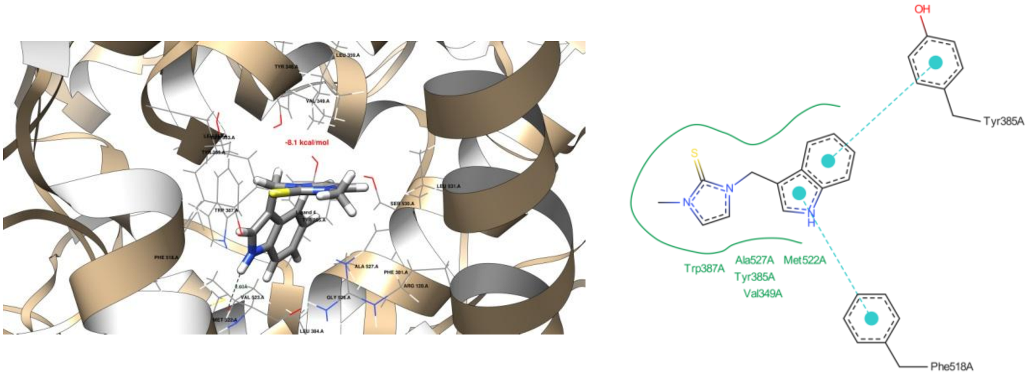

(**a**) (**b**)

Fig. S33. (**a**) The spatial interactions between the derivative **5** and the 4COX protein domain. A dark-green dashed line suggests a potential hydrogen bond formation between the domain residue MET 522 A (length 2.60 Å) and the ligand; (**b**) The proposed interactions between the 4COX protein's domain binding site and derivative **5** are indicated by green solid lines indicating hydrophobic contacts and aqua dashed lines indicating cation-pi interaction. This model is not indicating hydrogen bond formation between MET 522 A and the ligand.

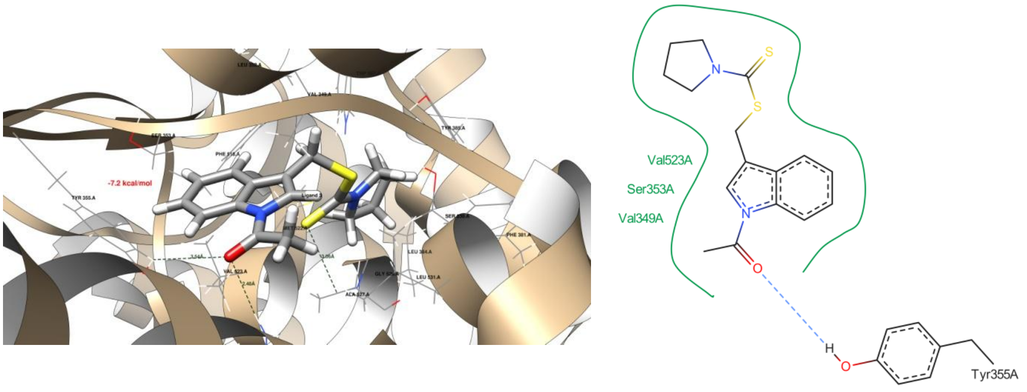

(**a**) (**b**)

Fig. S34. (**a**) The spatial interactions between the derivative **15** and the 4COX protein domain. A dark-green dashed line suggests a potential hydrogen bond formation between the domain residue TYR 355 A and the ligand (3.54 Å length). The other can be formed between the ARG 120 A protein's residue and the ligand with a length of 2.48 Å, indicating a higher probability of formation. The last one can be formed between the ALA 527 A residue and the Ligand 3 structure (3.06 Å length); (**b**) The proposed interactions between the 4COX protein's domain binding site and derivative **15**. Green solid lines indicate hydrophobic contacts, and a blue dashed line indicates a hydrogen bond. It is indicating a lower number of hydrogen bonds, probably due to the fact that hydrogen bond formation between ARG 120 A and ALA 527 A residues is more difficult due to the greater angle.

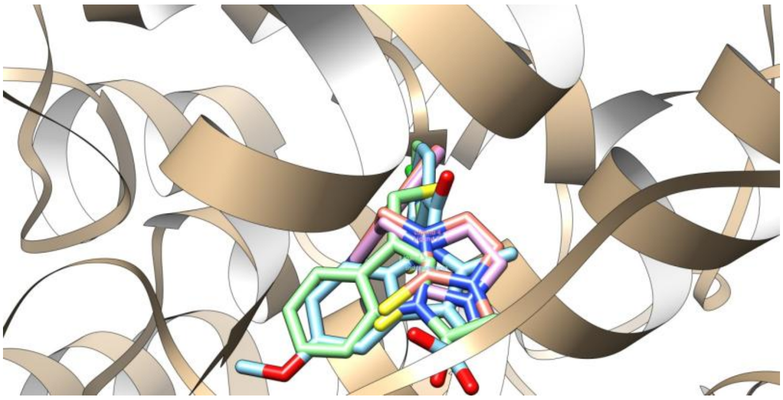

Fig. S35. The native ligand (indomethacin), redocked native ligand, and compounds **2**, **5**, and **15** in the binding site of the 4COX protein domain.
